# Host–Endosymbiont Genome Integration in a Deep-Sea Chemosymbiotic Clam

**DOI:** 10.1093/molbev/msaa241

**Published:** 2020-09-21

**Authors:** Jack Chi-Ho Ip, Ting Xu, Jin Sun, Runsheng Li, Chong Chen, Yi Lan, Zhuang Han, Haibin Zhang, Jiangong Wei, Hongbin Wang, Jun Tao, Zongwei Cai, Pei-Yuan Qian, Jian-Wen Qiu

**Affiliations:** 1 Department of Biology, Hong Kong Baptist University, Hong Kong, China; 2 HKBU Institute of Research and Continuing Education, Virtual University Park, Shenzhen, China; 3 Hong Kong Branch of the Southern Marine Science and Engineering Guangdong Laboratory (Guangzhou), The Hong Kong University of Science and Technology, Hong Kong, China; 4 Division of Life Science, Department of Ocean Science, The Hong Kong University of Science and Technology, Hong Kong, China; 5 Department of Infectious Diseases and Public Health, Jockey Club College of Veterinary Medicine and Life Sciences, City University of Hong Kong, Hong Kong, China; 6 X-STAR, Japan Agency for Marine-Earth Science and Technology (JAMSTEC), Yokosuka, Kanagawa Prefecture, Japan; 7 Sanya Institute of Deep-Sea Science and Engineering, Chinese Academy of Science, Sanya, Hainan, China; 8 MLR Key Laboratory of Marine Mineral Resources, Guangzhou Marine Geological Survey, China Geological Survey, Guangzhou, China; 9 State Key Laboratory of Environmental and Biological Analysis, Hong Kong Baptist University, Hong Kong, China

**Keywords:** cold seep, genome assembly, genome erosion, hydrothermal vent, Mollusca, symbiosis

## Abstract

Endosymbiosis with chemosynthetic bacteria has enabled many deep-sea invertebrates to thrive at hydrothermal vents and cold seeps, but most previous studies on this mutualism have focused on the bacteria only. Vesicomyid clams dominate global deep-sea chemosynthesis-based ecosystems. They differ from most deep-sea symbiotic animals in passing their symbionts from parent to offspring, enabling intricate coevolution between the host and the symbiont. Here, we sequenced the genomes of the clam *Archivesica marissinica* (Bivalvia: Vesicomyidae) and its bacterial symbiont to understand the genomic/metabolic integration behind this symbiosis. At 1.52 Gb, the clam genome encodes 28 genes horizontally transferred from bacteria, a large number of pseudogenes and transposable elements whose massive expansion corresponded to the timing of the rise and subsequent divergence of symbiont-bearing vesicomyids. The genome exhibits gene family expansion in cellular processes that likely facilitate chemoautotrophy, including gas delivery to support energy and carbon production, metabolite exchange with the symbiont, and regulation of the bacteriocyte population. Contraction in cellulase genes is likely adaptive to the shift from phytoplankton-derived to bacteria-based food. It also shows contraction in bacterial recognition gene families, indicative of suppressed immune response to the endosymbiont. The gammaproteobacterium endosymbiont has a reduced genome of 1.03 Mb but retains complete pathways for sulfur oxidation, carbon fixation, and biosynthesis of 20 common amino acids, indicating the host’s high dependence on the symbiont for nutrition. Overall, the host–symbiont genomes show not only tight metabolic complementarity but also distinct signatures of coevolution allowing the vesicomyids to thrive in chemosynthesis-based ecosystems.

## Introduction

Symbiosis with chemoautotrophic bacteria, first discovered ∼40 years ago in the gutless deep-sea giant tubeworm *Riftia pachyptila* (Cavanaugh et al. 1981), is now widely recognized as an important force shaping the diversity and evolution of many groups of invertebrates such as sponges, nematodes, crustaceans, annelids, and molluscs (Dubilier et al. 2008). Among them, the molluscan class Bivalvia has been proposed as a model system for studying the evolution of eukaryote-chemoautotrophic bacteria symbiosis due to the independent acquisition of chemosymbiosis in multiple lineages, various levels of intimacy with the symbiont, and wide distribution. Members from nine distinct families (Basterotiidae, Lasaeidae, Lucinellidae, Lucinidae, Mytilidae, Solemyidae, Teredinidae, Thyasiridae, and Vesicomyidae) have been reported to host chemosynthetic symbionts (Distel 1998; Roeselers and Newton 2012; Oliver 2013; Distel et al. 2017). These families represent a broad spectrum of phylogenetic groups within Bivalvia, which span from the early-diverging protobranchs (Solemyidae) to the more recently derived imparidentians (Vesicomyidae) (Bieler et al. 2014; Lemer et al. 2019). These symbionts exhibit different levels of association with their bivalve hosts, from attaching themselves to the ciliated zone of gill epithelial cells, to being trapped among the protruded gill cell membranes, and even inside specialized bacteriocyte cells in the gill (Dubilier et al. 2008). In addition to being dominant in deep-sea hydrothermal vents, cold seeps, and organic falls worldwide, bivalves hosting chemosynthetic symbionts are common in shallow-water environments with high organic contents such as seagrass beds and mangrove swamps, playing important roles in the energy flow and element cycling in these ecosystems (van der Heide et al. 2012; Petersen et al. 2017).

Vesicomyidae is a diverse family with >110 species (Krylova and Sahling 2010; Johnson et al. 2017) divided into two subfamilies, Vesicomyinae and Pliocardiinae. The subfamily Vesicomyinae, represented by only one genus (*Vesicomya*) of mostly small-sized clams (usually <1 cm in shell length), has been reported from both reducing and nonreducing habitats, and is not known to host chemoautotrophic symbionts. The subfamily Pliocardiinae, in contrast, comprises 14 genera of medium- to large-sized clams (some >30 cm) that inhabit various deep-sea chemosynthesis-based ecosystems; all of them harbor chemoautotrophic bacteria inside bacteriocytes in their gill epithelium. Pliocardiines typically live half-buried in reducing sediments or inhabit crevices of bare rocks, with their gills obtaining oxygen from the surrounding water, and their foot taking up hydrogen sulfide from the substrate (Cavanaugh et al. 2006; Childress and Girguis 2011; Hongo et al. 2016). Most of our knowledge about vesicomyid physiology as a response to the metabolic demand of endosymbiotic chemoautotrophs has come from studies of several species of pliocardiines, especially *Turneroconchamagnifica* (originally described as *Calyptogena magnifica*) and *Phreagena okutanii* (originally described as *Calyptogena okutanii*) (Johnson et al. 2017).

Endosymbionts of pliocardiine clams reside not only in their gills but also in their ovaries (Cary and Giovannoni 1993) and eggs (Kojima and Ohta 1997), indicating maternal transmission of symbionts to offspring. Due to the small number of symbiont DNA copies in each egg (Ikuta et al. 2016), this strategy of symbiont transmission creates a genetic bottleneck, promoting genetic drift and accumulation of deleterious mutations (Roeselers and Newton 2012). In line with evidences for their intimate relationship, the phylogenetic trees of pliocardiines and their symbionts exhibit coupling (Distel et al. 1994; Peek et al. 1998). This indicates coevolution between the two parties, although occasional lateral symbiont transmissions between hosts have been reported (Stewart and Cavanaugh 2009; Ozawa et al. 2017). Maternal symbiont transmission in pliocardiines is unique among chemosynthetic endosymbionts of invertebrates, such as other bivalve and gastropod molluscs as well as siboglinid tubeworms and gutless oligochaetes, which all laterally obtain their symbionts anew from the environment in each generation (Dubilier et al. 2008; Petersen et al. 2017).

Previous analyses of 16S ribosomal RNA (rRNA) sequences showed that the pliocardiine symbionts are sulfur-oxidizing gammaproteobacteria, and closely related to the thioautotrophic endosymbionts of bathymodiolin mussels and a thyasirid clam (Roeselers and Newton 2012; Duperron et al. 2013). To date, only two pliocardiine symbiont genomes have been published—*Candidatus* Ruthia magnifica associated with the hydrothermal vent clam *T.magnifica* (Newton et al. 2007), and *Candidatus* Vesicomyosocius okutanii associated with the seep- and vent-dwelling clam *P. okutanii* (Kuwahara et al. 2007; Watanabe et al. 2013). The two pliocardiine symbionts are very similar not only in devoting a large proportion of the genomic machinery to energy production and carbon fixation but also in retaining most metabolic pathways for amino acid and cofactor/vitamin biosynthesis. This is consistent with the expectation that the hosts, with a vestigial digestive system, are highly reliant on the symbionts for nutrition (Cavanaugh 1983; Krylova and Sahling 2010). Nevertheless, these symbionts have highly reduced genome sizes, having lost ∼60% protein-coding genes (PCGs) that are typically found in free-living thioautotrophs, as well as the endosymbiotic thioautotrophs of sibognilid tubeworms (Li, Liles, et al. 2018; Yang, Sun, et al. 2020), bathymodiolin mussels (Ponnudurai et al. 2017), and scaly-foot snails (Nakagawa et al. 2014). Notably missing in the pliocardiine symbionts are genes that encode DNA recombination and repair, which are critical for the bacteria to repair deleterious DNA mutations. This, together with the genetic bottleneck imposed by the small number of symbiont DNA copies transmitted between generations, is believed to have accelerated their genome reduction (Kuwahara et al. 2007; Newton et al. 2008).

These previous molecular studies have led to a deep understanding of the provision of energy and nutrients from chemosynthetic symbionts to their hosts, and the adaptions of the symbionts to living inside cells of the hosts. However, as pointed out by Li, Liles, et al. (2018) in a study of deep-sea tubeworms, sequencing of the host genome is necessary to understand how the host supports and regulates the symbionts. Given their maternal transmission mode and reduced symbiont genome size, one would expect a tighter coupling of the host/symbiont genomes in the pliocardiines than in other bivalves, snails, or tubeworms which adopt the horizontal symbiont transmission strategy and have genome sizes more similar to free-living thioautotrophs. In addition to complementing the nutrition of the symbionts and providing an efficient system to support gas exchange and transport for symbiosis, the hosts may have evolved special mechanisms such as constrained immune functions to accommodate the symbionts and horizontal gene transfer (HGT) from bacterial donors, as demonstrated in nonchemosynthesis-based systems (Moran and Bennett 2014).

In this study, we sequenced the genomes of *Archivesica marissinica* (Bivalvia: Vesicomyidae) and its bacterial endosymbiont (here named *Candidatus* Vesicomyosocius marissinica) by combining second- and third-generation sequencing technologies. *Archivesica marissinica* (originally described as “*Calyptogena*” *marissinica* by Chen et al. [2018] and transferred to *Archivesica* in Feng et al. [2018]) is a dominant species at the Haima cold seep, situated on the northwest continental slope of the South China Sea. Through analyzing the host genome, we provide the first evidence of HGT from bacteria to a bivalve mollusk. We also find several lines of evidences for early genome erosion in the clam as an adaptation to the symbiotic lifestyle, although genome erosion has been mainly used to describe the changes in bacterial genome after forming symbiosis with eukaryotes (Toft and Andersson 2010; Shigenobu and Wilson 2011). We also show the expansion/contraction in several gene families, which likely facilitated the establishment of successful chemoendosymbiosis. Through analyzing the symbiont genome, we reveal its nutrient provision to the host, as well as the evolutionary trajectory of genome size reduction. Furthermore, the resources generated in this study are valuable for comparative genomic and evolutionary studies of Mollusca, for which only a few high-quality genome assemblies are available (Halanych and Kocot 2017; Voolstra et al. 2017; Gomes-dos-Santos et al. 2020; Sun et al. 2020), despite the extremely high species diversity (>85,000 extant species) and morphological disparity (Kocot et al. 2011, 2020; Sigwart and Lindberg 2015; Aguilera et al. 2017) of this animal phylum.

## Results and Discussion

### Host Genome Assembly

**Fig. 1.**
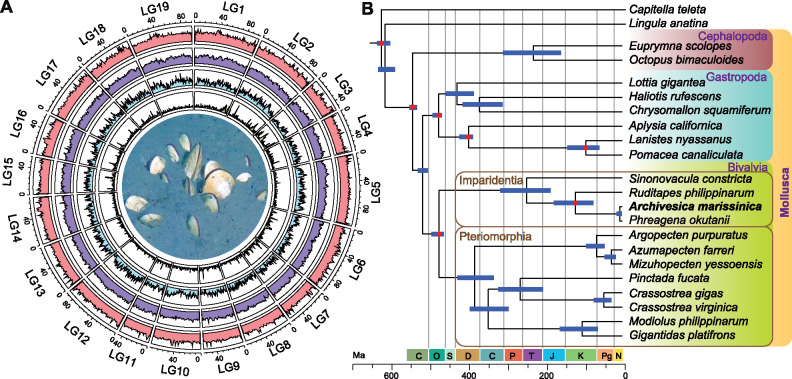
Genome landscape and phylogenetic position of *Archivesica marissinica*. (*A*) Circus plot of 19 linkage groups (corresponding to “chromosomes”) showing marker distributions at 1-Mb sliding windows from outer to inner circle: Illumina sequencing depth, repeat density, gene density, and pseudogene density. The center shows live *A. marissinica* clams half-buried in their natural habitat of cold seep sediment (also see supplementary fig. S1, Supplementary Material online). (*B*) Maximum-likelihood phylogenetic relationships among 20 molluscs with an annelid and a brachiopod as outgroups. The tree was calibrated at seven nodes (indicated by red dots) using fossils and geological events to reveal divergence times (more details including node values in supplementary fig. S3, Supplementary Material online). The data included 246 single-copy orthologs with a total of 62,499 amino acid positions and 3.6–32.6% gaps. The data mainly came from genome assemblies, except those of *Phreagena okutanii* which were transcriptome sequences (GIAT00000000; Lan et al. 2019). The tree, constructed using the LG+F+R8 substitution model, has a bootstrap support of 100 at all nodes. Blue lines indicate 95% confidence interval for divergence times.

Assembly of the sequences produced by second- and third-generation platforms (supplementary table S1, Supplementary Material online) resulted in a draft *A. marissinica* genome containing 19,871 scaffolds with an N50 of 162 kb (supplementary table S2, Supplementary Material online). Incorporating the Hi-C data resulted in a 1.52-Gb final assembly in which 78.8% of the scaffolds (=94.3% of genome size) were successfully anchored to 19 linkage groups, resulting in one of the few existing chromosome-level molluscan genome assemblies (fig. 1*A*). Analysis of the *A. marissinica* assembly using BUSCO (Simão et al. 2015) showed that it comprised 93.5% (91.8% complete and 1.7% fragmented) of the 978 universal single-copy orthologous metazoan genes (supplementary table S3, Supplementary Material online). The integrity of the assembly is supported by the successful mapping of >99% Illumina paired-end reads (supplementary table S4, Supplementary Material online). The *A. marissinica* genome assembly contained 28,949 PCGs with 87.0% complete and 5.0% fragmented BUSCOs (supplementary table S3, Supplementary Material online), of which 25,462 (88.0%) were successfully annotated using several public protein databases (supplementary table S5, Supplementary Material online).

### Phylogenetic Relationships and Divergence Times

A phylogenetic tree constructed using a total of 62,499 amino acids shows a topology of bivalve families congruent with the results of previous studies (Bieler et al. 2014; Lemer et al. 2019), except that all nodes had full bootstrap support (100) (fig. 1*B* andsupplementary fig. S3, Supplementary Material online). Calibration of the tree using fossils and geological events shows that the *Phreagena* and *Archivesica* lineages are young, diverging roughly 14.5 Ma in mid-Miocene. Vesicomyidae (in Glossoidea) split with Veneridae (in Veneroidea, represented by *Ruditapes philippinarum*) roughly 128.0 Ma (82.4–182.9 Ma) in the Early Cretaceous. Together with the divergence in Late Cretaceous (66–100 Ma) of nonsymbiotrophic vesicomyids and symbiotrophic pliocardiines estimated based on six genes (Johnson et al. 2017), our results support the hypotheses that vesicomyids had invaded the deep sea long before the rise of pliocardiines. The immediate ancestors of pliocardiines were likely asymbiotrophic but had already preadapted to the deep-sea environment, allowing their further divergence to become one of the extremely successful and diverse groups of bivalves inhabiting various chemosynthesis-based ecosystems.

### Structural Characteristics of the Host Genome

As with several well-assembled lophotrochozoan genomes, the *A. marissinica* assembly has a complete 11-gene *Hox* cluster and a complete 3-gene *ParaHox* cluster (supplementary fig. S4, Supplementary Material online). The order of these genes is identical to those of the inferred ancestral lophotrochozoan state (Wang et al. 2017), indicating that these genes responsible for modulating the patterning of the anterior–posterior body axis in Bilateria are conserved in this deep-sea vesicomyid clam. A chromosome-based macrosynteny analysis indicates a lack of major chromosomal rearrangements in the clade Imparidentia as shown by their moderate level of conservation index (CI=0.44–0.50) with the 17 presumed bilateral ancestral proto-chromosomes or ancestral linkage groups (ALGs) (Putnam et al. 2008), except for the fusion of ALG6 and ALG15, translocation of ALG2 and ALG4, and fragmentation of ALG16 (supplementary fig. S5, Supplementary Material online). The fact that multiple members of two major clades of Bivalvia, the clams (*A. marissinica*, *Ru. philippinarum*, and *Sinonovacula constricta*) in the clade Imparidentia and the scallop *Mizuhopecten yessoensis* in the clade Pteriomorphia, have 19 chromosomes is indicative that their common ancestor might have also possessed the same number of chromosomes. The high CI values (0.65–0.75; supplementary fig. S5, Supplementary Material online) between the imparidentian clams and the scallop also suggest that the *A. marissinica* genome has not undergone major karyotypic changes when compared with that of their inferred most recent common ancestor.

**Fig. 2.**
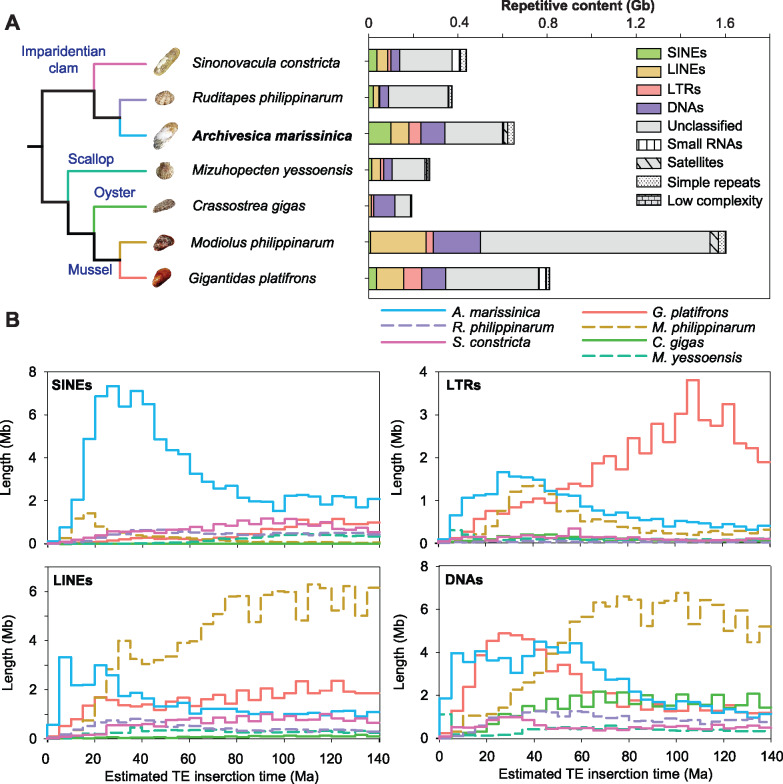
Composition of repetitive sequences including transposable elements (TEs) in bivalve genomes. (*A*) Composition of repetitive elements showing expansion of four TE classes (i.e., SINEs, LINEs, LTRs, and DNAs) in the *Archivesica marissinica* genome when compared with other imparidentian clam genomes. (*B*) Comparison of estimated insertion times of four TE classes among bivalves, showing that the *A. marissinica* genome is distinct in that the major bursts of TE insertion corresponded to the timing of the rise of chemoautotrophic symbiont-hosting pliocardiines ∼65 Ma and their subsequent diversification.

A comparison of the genomic structures among three imparidentian clams showed that the *A. marissinica* genome contains a larger amount of repetitive elements (641 Mb, 42.2% of the 1.52-Gb assembled genome size) than the genomes of *R. philippinarum* (371 Mb, 32.9% of the 1.12-Gb assembled genome size) and *S. constricta* (412 Mb, 33.7% of the 1.34-Gb assembled genome size). As the nonrepetitive genome region in the three clams are similar in total length (749–928 Mb), the expansion of these repetitive sequences has been largely responsible for the larger genome size in *A. marissinica* (fig. 2*A* andsupplementary table S6, Supplementary Material online). Among the repetitive sequences, four major groups of transposable elements (TEs)—DNA segments with a defined structure allowing them to change their locations in the genome—have expanded in the *A. marissinica* genome. Specifically, compared with the *R. philippinarum* genome, the *A. marissinica* genome includes more DNA transposons (109.2 vs. 38.8 Mb), long-terminal repeats (LTRs) (52.9 vs. 4.3 Mb), long-interspersed nuclear elements (LINEs) (81.6 vs. 24.9 Mb), and short-interspersed nuclear elements (SINEs) (99.3 vs. 20.6 Mb). To understand the temporal dynamics of TE activities in these bivalve genomes, we estimated the insertion times of TEs in these species based on comparative analysis of nucleotide substitution rates (Kimura 1980). Remarkably, there have been bursts of TE insertion activities in the pliocardiine clam lineage since 70 Ma (10–70 Ma for SINEs, 10–35 Ma for LINEs, and 10–50 Ma for LTRs and DNA transposons; fig. 2*B*), which corresponded to the timing of the rise of chemoautotrophic symbiont-hosting pliocardiines and their subsequent diversification (Distel 1998; Vrijenhoek 2013; Johnson et al. 2017).

**Fig. 3.**
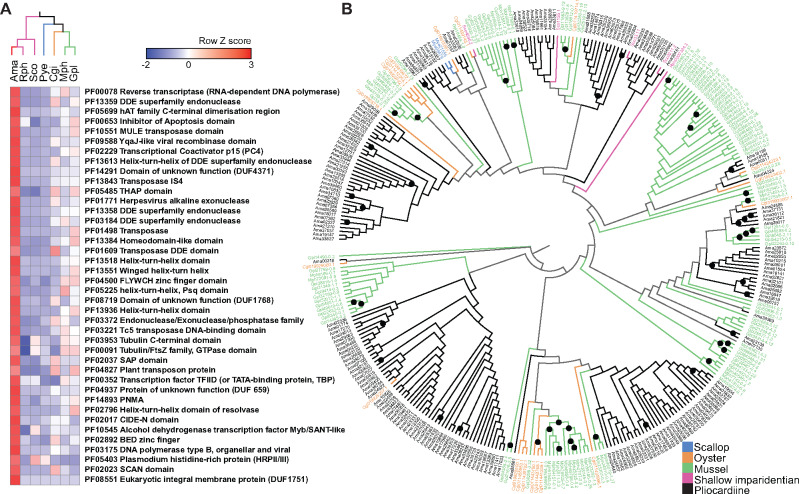
Comparison of gene families among selected bivalves. (*A*) Heat map of major annotated Pfam domains that are expanded in *Archivesica marissinica*, with multiple domains in a given gene being counted as one. (*B*) Maximum-likelihood tree constructed based on the VT+G4 substitution model showing lineage-specific expansion of the MULE transposase protein domain in the *A. marissinica* genome. Among the 335 sequences found in the seven bivalve genomes, 193 sequences came from *A. marissinica*, and 142 sequences from the other six species (Gpl: 83, Mph: 33, Cgi: 19, Sco: 4, Mye: 2, Rph: 1). Each sequence prefix represents the first letter of the genus name and the first two letters of the species name [Mye, *Mizuhopecten yessoensis* (scallop); Cgi, *Crassostrea gigas* (oyster); Gpl, *Gigantidas platifrons* (mussel); Mph, *Modiolus philippinarum* (shallow imparidentian); Rph, *Ruditapes philippinarum* (shallow imparidentian); Sco, *Sinonovacula constricta* (shallow imparidentian); Ama, *Archivesica marissinica* (pliocardiine)]. Nodes with 100 bootstrap support are labeled with a black dot. The full-size figure is deposited in Figshare.

Genomic comparisons indicated that gene family expansion may have driven the proliferation of TEs in the *A. marissinica* genome (fig. 3*A*), with the most highly expanded Pfam protein domains being retrotransposons (i.e., reverse transcriptase, RNA-dependent DNA polymerase), DNA transposases (i.e., DDE superfamily endonuclease, hAT family C-terminal dimerization region, MULE transposase domain), and endogenous viral elements (i.e., YqaJ-like viral recombinase domain, herpesvirus alkaline exonuclease) that can fuse with genes including transposases to generate genetic novelties (Inoue et al. 2018). For instance, the MULE transposase domain is significantly expanded in *A. marissinica*, with 193 copies, compared with only 1–83 copies in other bivalves (fig. 3*B*). Similarly, the reverse transcriptase family is also highly expanded in *A. marissinica* with 408 copies, compared with 1–192 copies in other bivalves (supplementary fig. S6, Supplementary Material online). In addition, the *A. marissinica* genome encodes a biogenesis machinery of Piwi-interacting RNA (piRNA) to inhibit TE transcription and invasion (supplementary fig. S7*A*, Supplementary Material online). An Argonaute family of Piwi genes (AUB/PIWI) silence TE activities by directing mature piRNAs to TE mRNA (Levin and Moran 2011), and therefore the expansion of PIWI proteins in *A. marissinica* after the bursts of TEs may have reduced their further invasion (supplementary fig. S7*B*, Supplementary Material online).

**Fig. 4.**
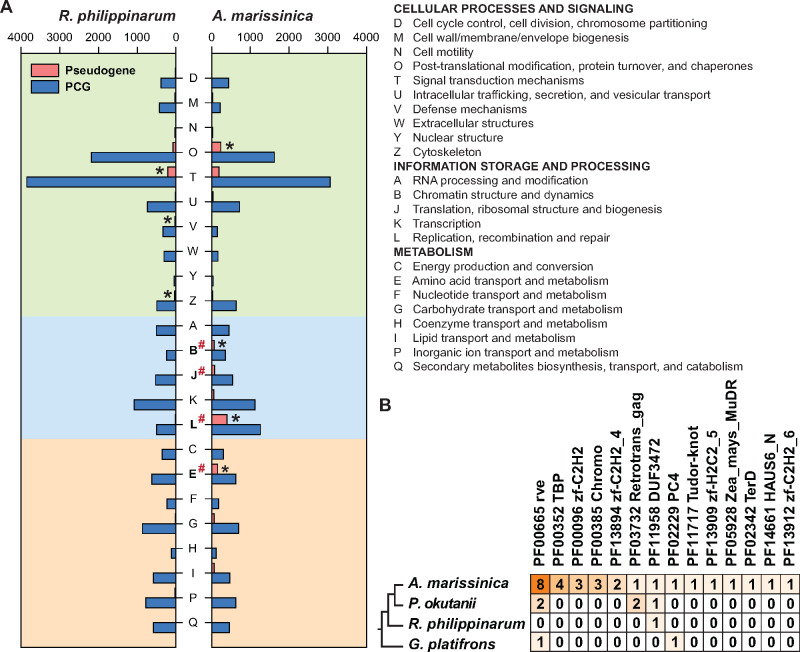
Pseudogenes and horizontally transferred genes. (*A*) Comparison of functional distribution of protein-coding genes (PCGs) and pseudogenes in the genomes of *Archivesica marissinica* and *Ruditapes philippinarum*. χ^2^ tests were used to determine the enriched clusters of orthologous groups (COGs) within and between the two imparidentian clams (*P *<* *0.05), with significantly different categories indicated by an asterisk and pound sign, respectively. (*B*) The numbers of horizontally transferred genes (HTGs) with bacterial origin (only those with Pfam annotation) in the *A. marissinica* genome, compared with the numbers of their orthologs in the *Phreagena okutanii* transcriptome and the *R. philippinarum* and *Gigantidas platifrons* genomes.

As TEs may undergo retrotransposition or insertion in the coding region and result in the formation of pseudogenes (nonfunctional DNA sequences that resemble functional genes) in eukaryotes, we compared the contents of genes and pseudogenes between the *R. philippinarum* and *A. marissinica* genomes that have substantially different amounts of TEs. Although the *R. philippinarum* genome contains a slightly higher number of PCGs (27,652), the *A. marissinica* genome possesses remarkably more pseudogenes (10,211 vs. 2,015) (fig. 4*A*), with more parent genes of the pseudogenes (2,528 vs. 1,078) and also a higher maximum number of pseudogenes per gene (190 vs. 38). This larger number of pseudogenes is related to the expansion of TEs, as TE-associated retrotransposition occurred in a much higher proportion (73.5%) of pseudogenes in *A. marissinica* than in *R. philippinarum* (37.9%). Furthermore, the insertion of SINE/tRNA-Deu-L2 (∼50 Ma) and LTR/Ngaro (∼65 Ma) into the processed pseudogenes seen in a large proportion of pseudogenes (59.4%) corresponded to the timing of the rise of chemosynthetic vesicomyids in the deep sea (supplementary table S5, Supplementary Material online; Distel 1998; Vrijenhoek 2013; Johnson et al. 2017). A functional classification shows that the distribution of genes and pseudogenes in the two bivalves is uneven among the clusters of orthologous group (COG) categories, with four (O, B, L, and E) categories overrepresented in *A. marissinica* (fig. 4*A*). Moreover, comparison of COG categories between the two species shows that the *A. marissinica* pseudogenes are significantly more enriched in chromatin structure and dynamics (B), translation, ribosomal structure, and biogenesis (J), DNA replication (L), and amino acid (E). The substantially higher number of pseudogenes and their enrichment in these critical functional categories may have profound effects on the host’s genomic DNA integrity, nutrient production, transport, and metabolism. Analysis of the transcriptome data showed that 4,616 of the *A. marissinica* pseudogenes were expressed (supplementary table S5, Supplementary Material online). Among the 23 COG functional categories of pseudogenes, 16 exhibited higher expression in the gill than in other tissues (supplementary fig. S8, Supplementary Material online), indicating their potential involvement in regulating gene functions (Cheetham et al. 2020) in this endosymbiont-hosting organ.

Due to the differences in transcriptional and translational control mechanisms between bacterial and eukaryotic genes, it is generally considered difficult to incorporate bacterial DNA into the eukaryotic genome (Danchin 2016). HGT (or lateral gene transfer) from bacteria, however, has been reported in a few groups of invertebrates such as insects and rotifers, and recognized as an important force shaping the evolution of these invertebrate genomes (Husnik and McCutcheon 2018). Nevertheless, no HGT event has been reported in Mollusca, one of the most diverse phyla of invertebrates (Boto 2014). Since the obligate symbiotic association in *A. marissinica* might have promoted gene transfer from the bacteria to the host, we searched its predicted gene models against the NCBI NR database and identified candidates of HGT based on the calculation of the index of HGT for each gene (full list of 173 initial HGT candidates in supplementary table S7, Supplementary Material online). Filtering the results using several stringent criteria to eliminate analytical artifact and contamination (details in Materials and Methods and supplementary fig. S9, Supplementary Material online) resulted in the discovery of 28 putative HTGs with bacterial origins in the *A. marissinica* genome (fig. 4*B* andsupplementary table S7, Supplementary Material online).

Remarkably, none of the putative *A. marissinica* HTGs shows sequence homology with its contemporary symbiotic gammaproteobacteria, or the symbiont genomes of other pliocardiine clams. Instead, they show sequence homology with other symbiotic gammaproteobacteria of distantly related bivalve lineages (i.e., the awning-clam *Solemya* and the deep-sea mussel *Bathymodiolus*). This result, together with the finding of nine homologs of these HTGs in the transcriptome of another pliocardiine, *P. okutanii*, indicates that the HGT events likely took place in ancestral chemoautotrophic symbionts that were once associated with several deep-sea bivalve lineages. Nevertheless, roughly two-thirds of the HGTs in these two species are not homologous, which indicates their acquisition after the divergence of these two genera in mid-Miocene (∼14.5 Ma). The majority of HTGs in *A. marissinica* contain the integrase core domain (PF00665) or the retrotransposon gag protein domain (PF03732) (fig. 4*B*), which are often associated with TEs and are known to affect genome evolution (Peccoud et al. 2017). Specifically, integrases are viral enzymes that mediate the integration of a viral/bacteriophage DNA into the host genome, and allow the host to produce viral proteins. Gag proteins form virus-like structures in the cytoplasm for reverse-transcription and retrotransposon insertion (Liu et al. 2004). In addition, three of the HTGs contain a C_2_H_2_-type zinc finger domain, which is a transcription factor that modulates the functions of many groups of metazoan genes (Seetharam and Stuart 2013). Moreover, 13 of the HTGs possessing unknown Pfam domains are transcriptionally active (supplementary table S7, Supplementary Material online), which should be future targets of functional characterization.

### Host Gaseous Transport System That Likely Facilitates Symbiosis

To support the active metabolism of the holobiont, the bivalve host must be able to meet its own demand for oxygen, as well as the symbiont’s demand for hydrogen sulfide, oxygen, and inorganic carbon. Previous studies have found that a ∼15,000-kDa lipoprotein called sulfide-binding component (SBC) with high sulfide-binding affinity in the serum of several pliocardiines, including *Turneroconchamagnifica* and *Ectenagena elongata* (Arp et al. 1984; Zal et al. 2000; Childress and Girguis 2011). However, solemyids and lucinids do not seem to possess SBC; they rely on hemoglobins (Hbs) for both oxygen and hydrogen sulfide transport (Doeller et al. 1988; Kraus and Wittenberg 1990; Yuen et al. 2019). Since neither DNA nor protein sequences of the giant SBC protein have been characterized, it is unknown whether its homolog is present in the *A. marissinica* genome. Below, we focus on the description of the inorganic carbon uptake and transport system as well as Hbs in *A. marissinica.*

Inorganic carbon, in the form of CO_2_, ${\text{CO}}_{3}^{2-}$, or ${\text{HCO}}_{3}^{-}$, is required for carbon fixation in the symbionts. Previous studies of *P. okutanii* have indicated that its inorganic carbon transport system comprised two membrane-associated carbonic anhydrases (MCAs), two cytoplasmic carbonic anhydrases (CCAs), and four solute carrier (SLC) family 4 bicarbonate transporters (SLC4COs) (Hongo et al. 2013, 2016). The MCAs in both symbiotic and asymbiotic cells catalyze the conversion of the membrane-impermeable ${\text{HCO}}_{3}^{-}$ to permeable CO_2_ uptake from seawater and hemolymph, and SLC4COs on the inner cellular membrane transport ${\text{HCO}}_{3}^{-}$ to the hemolymph. Inside the bacteriocyte, CCAs catalyze the conversion of ${\text{HCO}}_{3}^{-}$ to CO_2_ (Hongo et al. 2016).

**Fig. 5.**
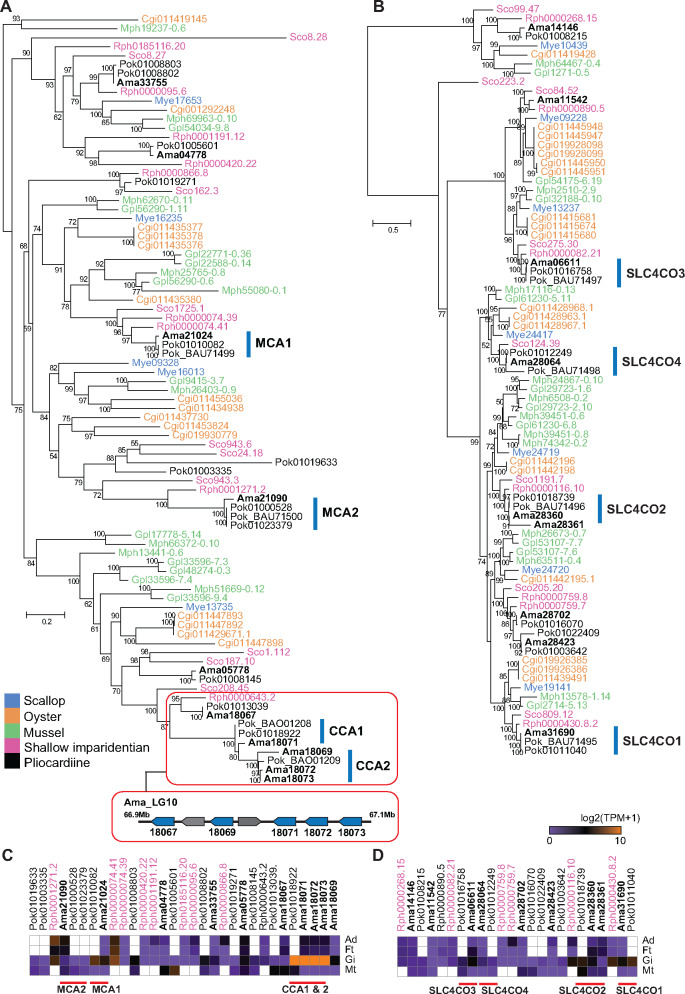
Genes involved in the uptake and transport of inorganic carbon in bivalves. (*A*) Maximum-likelihood tree constructed based on the WAG+R4 substitution model of membrane-associated carbonic anhydrases (MCAs) and cytoplasmic carbonic anhydrases (CCAs). CCAs inside a box are those that have undergone tandem duplications. The arrangements of five CCAs on LG10 of *Archivesica marissinica* are indicated in a bar, with two non-CCA genes also located on this scaffold. (*B*) Maximum-likelihood tree constructed based on the LG+R4 substitution model of solute carrier family 4 bicarbonate transporters (SLC4COs). Numbers on nodes are bootstrap values (>50). *Archivesica marissinica* sequences are highlighted in bold type. (*C*) and (*D*) shows the tissue-specific expression (i.e., Ad, adductor muscle; Ft, foot; Gi, gills; Mt, mantle) of CCAs and SLC4COs in three clams (Ama, Pok, Rph), respectively. Mye, *Mizuhopecten yessoensis* (scallop); Cgi, *Crassostrea gigas* (oyster); Gpl, *Gigantidas platifrons* (mussel); Mph, *Modiolus philippinarum* (shallow imparidentian); Rph, *Ruditapes philippinarum* (shallow imparidentian); Sco, *Sinonovacula constricta* (shallow imparidentian); Ama, *Archivesica marissinica* (pliocardiine).

Our phylogenetic analysis of bivalve CAs revealed the homologs of all these *P. okutanii* carbonic anhydrases and bicarbonate transporters in the *A. marissinica* genome (fig. 5). Among them, Ama21024 and Ama21090 each carry a signal peptide and are closely related to *P. okutanii* MCAs (fig. 5*A*). Remarkably, there is an expansion of CCAs in the two deep-sea pliocardiine clams, with three *P. okutanii* CCAs and four *A. marissinica* CCAs being nested in a terminal clade of the tree. This phylogenetic pattern, the proximity of these genes in the same chromosomal scaffold, and the high expression of three of them (Ama18071–Ama18073) indicate that the more ancestral CCA gene of *A. marissinica* (Ama18067) has undergone tandem duplications and co-opted to facilitate the conversion of inorganic carbon inside the vesicomyid bacteriocytes after the divergence of pliocardiine clams from the ancestor of *Ru. philippinarum* (fig. 5*A* and *C*). Interestingly, CCA genes in the deep-sea mussel *Gigantidas platifrons* (previously *Bathymodiolus platifrons*), which harbors endosymbiotic bacteria in its gill, have also undergone duplication, and two of them (Gpl33596-7.3 and Gpl48274-0.3) are highly expressed in the gill (Sun et al. 2017). This indicates the expansion and co-option of CCAs into bacteriocytes as a convergent evolutionary mechanism in deep-sea bivalves hosting chemoautotrophic symbionts. In contrast to CCAs, SLC4COs have not undergone expansion in the pliocardiine clams (fig. 5*B*), suggesting these ${\text{HCO}}_{3}^{-}$ transporters are evolutionarily conserved. Although the MCAs and SCL4COs are not highly expressed, they are transcriptional active, which supports the hypotheses that they are involved in inorganic carbon uptake and transportation in the gill (Hongo et al. 2016).

**Fig. 6.**
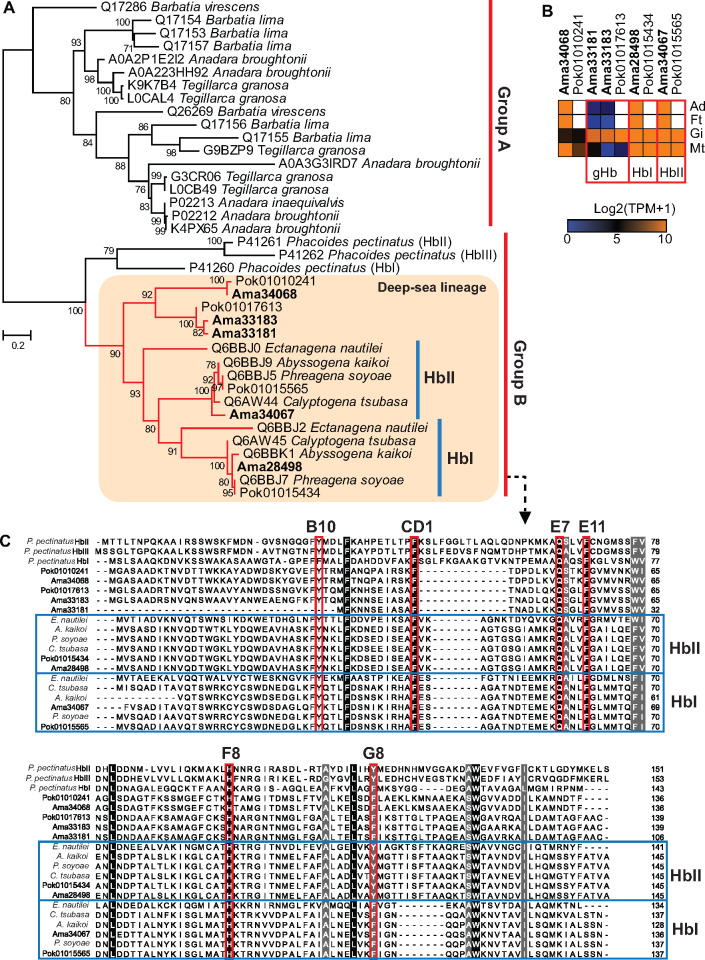
Hemoglobins (Hbs) in selected deep-sea and shallow-water bivalves. The shallow-water bivalve sequences were downloaded from UniPort. (*A*) Maximum-likelihood tree constructed based on the WAG+G4 substitution model. Numbers on nodes denoting bootstrap supports (>50). (*B*) Expression of Hbs in different tissues (Ad, adductor muscle; Ft, foot; Gi, gill; Mt, mantle) of *Archivesica marissinica* (Ama) and *Phreagena okutanii* (Pok) with unavailable data shown in white color. Only Ama33181, Ama33183, and Pok01017613 are specifically expressed in the gill, therefore are called gill hemoglobin (gHb). (*C*) Alignment of Hbs from *Phacoides pectinatus* and deep-sea pliocardiine clams with the heme contact and ligand-binding residues highlighted in boxes, and their positions indicated by numbers (B10, CD1, E7, E11, F8, G8).

Although Hbs are not as commonly used as oxygen-transport proteins in molluscs as in vertebrates (hemocyanin being the predominant oxygen-transport protein in molluscs), they have been reported from several distantly related bivalve families including Solemyidae (clade Protobranchia), Arcidae, and Lucinidae (clade Pteriomorphia), and Vesicomyidae (clade Imparidentia) (Doeller et al. 1988; Kraus and Wittenberg 1990; Suzuki et al. 2000; Childress and Girguis 2011). A phylogenetic analysis of the five Hbs we found in the *A. marissinica* genome and other bivalve Hb sequences showed that they can be divided into two main groups—group A containing the sequences from asymbiotic shallow water bivalves, and group B including three sequences from the symbiotic mangrove clam *Phacoides pectinatus* (Lucinidae), and all vesicomyid sequences (fig. 6*A*). Within group B, the three *Pha. pectinatus* Hbs (HbI, HbII, and HbIII) form a clade sister to a terminal clade containing all 17 vesicomyid Hbs, indicating lineage-specific expansion of Hbs in deep-sea vesicomyid clams. Consistent with the long divergence time between these two families (∼460 Ma, Bieler et al. 2014), all vesicomyid Hbs showed low homology with the *Pha. pectinatus* Hbs (<30% identity; supplementary fig. S10, Supplementary Material online). Nevertheless, all group B sequences possess conserved heme contact residues (Phe CD1 and proximal His F8) and ligand-binding residues (distal Gln E7, Tyr B10, and Phe E11) (fig. 6*C*) that have been shown to exhibit strong oxygen-binding capability in *Pha. pectinatus* Hbs (Gavira et al. 2008), suggesting that all vesicomyid Hbs possess oxygen-binding capacity. Among the five *A. marissinica* Hbs, two (Ama34067 and Ama28498) are clustered with other vesicomyid Hbs (HbIs and HbIIs) previously characterized as having oxygen-binding capacity (Suzuki et al. 2000) in a terminal clade. These two *A. marissinica* genes are highly expressed in all four tissues (fig. 6*B*), further supporting their active roles in oxygen binding and transport.

Furthermore, three *A. marissinica* Hbs (Ama34068, Ama33181, and Ama33183) exhibit tissue-specific expression patterns (fig. 6*B*), indicating they may function in oxygen binding, transport, or storage in different tissues. These three *A. marissinica* sequences differ from the vesicomyid HbIs and HbIIs in having a more reduced N-terminal (fig. 6*C*), a feature that has been associated with the change from oxygen binding to sulfide binding in some organisms (Rizzi et al. 1996; Nicoletti et al. 2010). As such, these three Hbs might also be involved in sulfide binding and transport. Overall, our analysis reveals three novel vesicomyid Hbs that are different from the well-characterized HbI and HbII, providing candidates for future experimental characterization of their functions.

### Structural Characteristics of the Symbiont Genome

Metagenomic analysis of the 86,641 bacterial 16S rRNA sequences revealed a single phylotype of gammaproteobacteria in the gill tissue of *A. marissinica* (supplementary fig. S11, Supplementary Material online). Hybrid assembly recovered the symbiont genome of *A. marissinica* as a single 1.03-Mb scaffold (GenBank accession number CP054877; supplementary table S8 and fig. S12, Supplementary Material online). CheckM analysis showed that the symbiont genome has a completeness of 94.84% and free from contamination. The symbiont genome, which we name as *Candidatus* Vesicomyosocius marissinica, encodes 1,021 genes [935 PCGs, 47 pseudogenes, a single operon of three rRNAs, and 36 transfer RNAs (tRNA)]. Compared with the free-living relative *Thiomicrospira crunogena*, the *Ca.* V. marissinica genome is greatly reduced (1.03 vs. 2.43 Mb) with erosion of genes for cellular envelope, signal transport, environmental sensing, motility, cell cycle control, and recombination (supplementary table S9, Supplementary Material online). However, the *Ca.* V. marissinica genome retains genes involved in sulfur oxidation (Sox), including dissimilatory sulfite reductase (*dsr*), adenylylsulfate reductase (*apr*), cytochrome *c* oxidase, and iron–sulfur clustering proteins.

Whole-genome alignment indicated that the pliocardiine symbiont genomes are similar, but the *Ca.* V. marissinica genome has a higher similarity with the *Ca.* V. okutanii genome (96.5%) than the *Ca.* R. magnifica genome (82.2%) (supplementary table S8 and fig. S13, Supplementary Material online). A genomic region of *Ca*. R. magnifica that encodes 20 genes/pseudogenes for synthesis of the cell envelope components polysaccharides and peptidoglycan is missing in both *Ca.* V. okutanii and *Ca.* V. marissinica, indicating their loss occurred after the split between the *Archivesica*+*Phreagena* clade and the *Turneroconcha *clade ∼40 Ma in mid-Eocene (Johnson et al. 2017) (supplementary figs. S14 and S15, Supplementary Material online). On the other hand, Newton et al. (2008) found that the *Ca.* V. okutanii genome encodes four dissimilatory nitrate reductase subunits (*narGHIJ*) that are missing the *Ca.* R. magnifica genome, and suggested that retaining the ability of nitrate uptake for use as a terminal electron acceptor is an adaptation of *Ca.* V. okutanii to the hypoxic cold seep environment. We also found all four nitrate reductase genes in the *Ca.* V. marissinica genome (supplementary table S10, Supplementary Material online). However, since *P. okutanii* inhabits both vents and seeps (Watanabe et al. 2013) and the *Archivesica*+*Phreagena* clade includes both vent and seep pliocardiines (e.g., *Ectenagena laubieri, A. kawamurai*) with closely related symbionts (supplementary fig. S16, Supplementary Material online), it is more likely that these nitrate metabolism genes are present in all species of this clade, rather than being a seep-specific adaptation. Further testing these alternative hypotheses requires the sequencing of the symbiont genome of at least one more vent-dwelling pliocardiines in the (*Archivesica*+*Phreagena*) clade in the future.

### Host–Symbiont Interdependence in Metabolism

Given the host’s high reliance on *Ca.* V. marissinica, below, we characterize the symbionts’ major metabolic pathways and their interactions with the host. Integrating the host/symbiont genome and transcriptome data provided a holistic view of the symbiosis, including potential host control mechanisms and the relative importance of alternative metabolic pathways.

Sulfur oxidation, which produces energy to drive carbon fixation and other metabolic processes, has been suggested to proceed through either the incomplete Sox pathway (with SoxXYZA and SoxB but without SoxCD) or the reverse dissimilatory sulfite reductase (rDsr) pathway in both *Ca.* R. magnifica and *Ca.* V. okutanii (Kuwahara et al. 2007; Newton et al. 2007; Harada et al. 2009), indicating their metabolic flexibility in response to the availability of different sulfur sources. We found 29 sulfur metabolic genes in the *Ca.* V. marissinica genome, including those involved in the Sox and rDsr pathways, indicating these sulfur metabolic pathways are conserved in pliocardiines (supplementary table S11, Supplementary Material online). Analysis of the symbiont transcriptome showed that Sox genes represented the highest number of bacterial transcripts (10.9%), highlighting the physiological importance of Sox in its metabolism (supplementary table S12, Supplementary Material online). Furthermore, both rDsr and Sox pathways were transcriptionally active in *Ca.* V. marissinica, consistent with the results from *Ca.* V. okutanii (Ohishi et al. 2016). Nevertheless, we found that the transcripts of *dsrAB* (catalyzing sulfide to sulfite) were 18.8 times as abundant as the *soxYZ* transcripts (catalyzing thiosulfate to sulfate). In addition, *aprAB* (catalyzing sulfite to adenosine-5′-phosphosulfate) and sulfate adenylyltransferase (*sat*) (catalyzing adenosine-5′-phosphosulfate to sulfate) were both highly expressed. These results indicate that the rDsr pathway is more prominent than the Sox pathway in *Ca.* V. marissinica, in agreement with the result of a previous physiological study showing quicker depletion of hydrogen sulfide than thiosulfate in the blood of *Turneroconcha magnifica* (Childress et al. 1991). Similarly, the thioautotrophic symbionts of the awning-clam *Solemya velum* (Stewart et al. 2011) and the tubeworms *Riftia pachyptila* (Gardebrecht et al. 2012) and *Paraescarpia echinospica* (Yang et al. 2020) expressed much higher levels of *dsrAB* than *soxYZ*. In contrast, the thioautotrophic symbionts of the deep-sea mussel *Bathymodiolus azoricus* expressed SoxYZ proteins more than DsrAB proteins (Ponnudurai et al. 2017). The differences in the use of alternative Sox pathways in these animals may be related to their physiological and behavioral adaptations. Pliocardiines and tubeworms are able to extend their foot or posterior end deep into rock crevices and sediments, and *So. velum* lives inside sediment burrows and accesses hydrogen sulfur from below the seafloor. On the other hand, *Bathymodiolus* mussels such as *B. azoricus* typically inhabit the surface of deep-sea authigenic carbonate rocks or sediments, indicating that they primarily take up hydrogen sulfide from seawater, usually at lower levels than in the reducing sediment.

Previous studies discovered a modified version of the Calvin–Benson–Bassham cycle for carbon fixation and storage in two pliocardiine symbiont genomes (Kuwahara et al. 2007; Newton et al. 2007). In the *Ca.* V. marissinica genome, we found the homologs of key enzymes in this pathway, including ribulose 1,5-bisphosphate carboxylase–oxygenase form II (RuBisCO) (*cbbM*), phosphoglycerate kinase (*pgk*), glyceraldehyde 3-phosphate dehydrogenase A (*gapA*), triosephosphate isomerase (*tpi*), fructose-bisphosphate aldolase (*fba*), transketolase (*tkt*), phosphoribulokinase (*prk*), indicating that this carbon fixation and storage pathway is conserved in pliocardiines. Using the *Ca.* V. marissinica metatranscriptome, we provided evidence that this pathway is transcriptionally active, with the RuBisCo *cbbM*, the *fba*, and the *gapA* genes being among the most highly expressed genes, accounting for 0.8%, 1.3%, and 0.8% of the total bacterial transcripts, respectively (supplementary table S13, Supplementary Material online).

Like most other sequenced chemosynthetic symbiont genomes (except those of the awning-clam *Solemya* and the scaly-foot snail *Chrysomallon squamiferum*), *Ca.* V. marissinica possesses a tricarboxylic acid cycle that lacks 2-oxoglutarate dehydrogenase (*odh*) and malate dehydrogenase (*mdh*) genes (supplementary table S14, Supplementary Material online). The absence of *odh* has been suggested to prevent oxidation of organic carbon for energy production, therefore is characteristic of obligate autotrophy (Wood et al. 2004; Dmytrenko et al. 2014; Ponnudurai et al. 2017). Since the *A. marissinica* genome encodes a phosphoenolpyruvate carboxykinase (*PCKA*) and *MDH* that catalyze the conversion of phosphoenolpyruvate to oxaloacetate and malate, and citrate transporters (Marty-Teysset et al. 1995) can move these products to the bacteriocyte’s vacuole, the missing of *mdh* in the symbiont should not prevent the production of its downstream intermediates for biosynthesis of amino acids and cofactors. Importantly, these results mean that the host can exert control on these biosynthesis processes and regulate the symbiont population.

Despite their reduced genome size, *Ca.* V. marissinica encodes complete gene sets for the biosynthesis of 19 common amino acids, and lacks only the thyroid hormone receptor beta (*thrB*) gene for synthesizing threonine, similar to *Ca.* R. magnifica and *Ca.* V. okutanii (Kuwahara et al. 2007; Newton et al. 2007). Nevertheless, our transcriptome data provided new evidence that these deep-sea pliocardiine symbionts likely possess the capability to biosynthesize all 20 common amino acids. Despite the lack of *thrB* to catalyze the reaction from homoserine to homoserine-P, threonine synthase (*thrC*) that catalyzes the conversion from homoserine-P to threonine is highly transcribed, indicating the substrate supporting this reaction is available (supplementary table S14, Supplementary Material online). The active expression of threonine dehydratase that catalyzes the conversion of threonine into alpha-ketobutyrate and ammonia further indicates threonine is present in the symbiont. Although the host genome encodes a threonine aldolase (*ItaE*) that catalyzes the interconversion between glycine and threonine, the transcription of this gene in the gill is very low, indicating the host is unlikely to be the source of threonine for the symbiont. Overall, these lines of evidence suggest that the symbiont encodes an uncharacterized gene that functions in the same way as *thrB*.

Although the three available deep-sea pliocardiine endosymbiont genomes are versatile in amino acid biosynthesis, they have lost most of the regulators for these biosynthesis pathways (supplementary table S15, Supplementary Material online). Of the 16 regulatory genes for amino acid biosynthesis that are present in *Escherichia coli*, we found only one intact gene (*himA*) and one pseudogene (*lysR*) in all three symbionts, and one more intact gene (*metR*) in *Ca.* R. magnifica, compared with eight in their free-living relative *T. crunogena*. This suggests that during the evolution of endosymbiosis, the loss of these regulators has been selected in favor of overproduction of amino acids by the pliocardiine symbionts. Like in the well-studied *Buchnera*-aphid symbiosis, *Ca.* V. marissinica may rely on the host’s transcription factors, miRNAs, and signal pathways to control the supply of substrates and regulate the biosynthesis of amino acids by the symbiont (Moran et al. 2005; Shigenobu and Wilson 2011).

### The Source and Acquisition of Nutrients in the Host

Due to their supposedly total reliance on endosymbiosis for nutrition, pliocardiines only possess a vestigial digestive system (Le Pennec et al. 1990). Here, we provide evidence that these deep-sea bivalves may not be able to digest phytoplankton-derived organic particles, further supporting their reliance on their endosymbionts for nutrition. Genomes of bivalves that filter-feed phytoplankton encode multiple enzymes with the glycosyl hydrolase family (GHF) domains that can catalyze the hydrolysis of complex polysaccharides especially cellulose (Sun et al. 2017), and many of these enzymes are highly expressed in their digestive tract (Gerdol et al. 2017). Our genomic comparison showed that several GHF families are contracted or completely missing in the *A. marissinica* genome. Specifically, the *A. marissinica* genome does not encode any GHF1, GHF5, and GHF10, compared with on an average 5.8, 4.7, and 5.2 genes in these three families, respectively, in other bivalve genomes (supplementary table S16, Supplementary Material online). In addition, this species encodes only three GHF9 genes, in contrast to on an average 8.2 GHF9 genes in other bivalves. Given the high quality of the *A. marissinica* genome assembly and the scattered distribution of many GHFs in the genomes of other species compared, the lack of these GHFs in the assembled genome is unlikely to be the result of sequencing bias or assembly error. None of these GHF families is contracted in the deep-sea mussel *Gigantidas platifrons* which, although rely largely on its endosymbionts for nutrition, retains the ability to digest organic particles originally produced in the surface water (Sun et al. 2017). This indicates that the contraction of GHF families in the *A. marissinica* genome is an adaptation linked to a higher reliance on its symbionts for nutrition.

Despite its diverse metabolic capabilities, *Ca.* V. marissinica, like *Ca.* R. magnifica and *Ca.* V. okutanii (Kuwahara et al. 2007; Newton et al. 2007), has lost Type I and Type II secretion systems and porins for transporting nutrients (i.e., amino acids and sugars) to the bacteriocyte (supplementary table S17, Supplementary Material online). Based on the higher lysozyme activities in the gill of symbiont-bearing pliocardiines than in asymbiotic bivalves, it has been suggested that the hosts mainly obtain nutrients from their symbionts by direct digestion (Fiala-Medioni et al. 1994; Kuwahara et al. 2007). However, since these symbionts do not make cell wall due to the reduced biosynthesis pathways for peptidoglycans, Newton et al. (2008) suggested the lysozymes in these clams may be used for defense against pathogens, rather than for symbiont digestion, and the host may use proteases or reactive oxygen mechanisms to digest the symbionts. Our transcriptome analyses showed that cathepsins that degrade proteins are highly expressed in the host gill (supplementary table S18, Supplementary Material online), suggesting the host may indeed digest the symbionts using proteases. Moreover, we found that “milking,” considered as an important nutrient acquisition strategy for the shallow water lucinid *Loripes orbiculatus* (Yuen et al. 2019), could be an overlooked strategy for pliocardiines to acquire nutrients from their symbionts. *Ca.* V. marissinica retained 62 substrate transporters, among them 16 were highly transcribed (>300 transcripts per kilobase million), indicating the symbiont is able to exchange molecules with the host (supplementary table S17, Supplementary Material online). Among the substrate transporters, YajR in the major facilitator superfamily and several genes in the ATP-binding cassette superfamily are known to export amino acids and organic acids in the bacterial symbiont of an aphid (Charles et al. 2011). Furthermore, the *A. marissinica* genome encodes 180 transporter genes belonging to 30 SLC families that rely on ion gradients to drive solutes across the cell membrane (supplementary table S19, Supplementary Material online). Among them, the SLC21 family of organic transporters is expanded compared with other bivalves (25 copies vs. 13–20 copies) (supplementary table S20, Supplementary Material online). Significantly, 55 of the host SLC transporters are clearly expressed in the gill, with diverse substrate specificities such as amino acids, carbohydrates, ascorbic acid, monocarboxylates, Na^+^-sulfate/carboxylate, and organic anions and cations. This result is consistent with previous studies showing that expressional enrichment of several SLC families (5, 6, 13, 16) in the symbiont-bearing root of the bone-eating zombie worm *Osedax japonicus* (Miyamoto et al. 2017), and in the gill of the lucinid bivalve *L. orbiculatus* (Yuen et al. 2019). Phylogenetic analyses of the enriched lutamate/neutral amino acid transporter family (SLC1; Kanai and Hediger 2004) and the organic anion transporting polypeptide transporter family (SLC21; Hagenbuch and Meier 2004) showed that they have undergone duplication in the deep-sea pliocardiine lineage compared with shallow-water imparidentian clams (supplementary fig. S17, Supplementary Material online). The expansion of these gene families and their active transcription in the *A. marissinica* gill thus likely indicate their involvement in “milking” nutrients from the symbionts (supplementary table S21, Supplementary Material online).

### Influence of Symbiosis on the Host’s Immune System

Examination of the *A. marissinica* genome assembly indicates its immune system may have been remodeled to accommodate its obligate endosymbiont. The immune-related functional categories “response to bacterium,” “apoptosis,” and “Toll and Imd signaling pathway” are all contracted in the *A. marissinica* genome (supplementary table S22, Supplementary Material online). Furthermore, among the 59 Pfam domains associated with innate immunity, eight domains related to either immune recognition or generation of immune effectors are contracted (supplementary table S23, Supplementary Material online). These results parallel the simplification in the immune system of insects hosting obligate symbiotic bacteria, which has been suggested to facilitate the maintenance of the symbionts by preventing the initiation of the host’s immune responses (Shigenobu and Wilson 2011; Masson et al. 2015). For instance, peptidoglycan recognition proteins (PGRPs) are a group of pattern recognition proteins that can recognize the peptidoglycan cell wall of bacteria. Their activation can initiate a series of immune reactions resulting in the death of bacteria. There are only three PGRPs genes in the *A. marissinica* genome, compared with six to 20 such genes in other bivalve genomes (supplementary table S23, Supplementary Material online). Similarly, the C-type lectin superfamily that functions in pattern recognition as the first line of defense against pathogens is significantly contracted, with only 36 genes in the *A. marissinica* genome compared with 121–438 in other bivalves. This contraction of the PGRP gene family reduction in *A. marissinica* (hosting *Ca.* V. marissinica without a cell wall) is in contrast to the expansion of the same gene family in the deep-sea mussel *G. platifrons* (hosting symbiotic bacteria with an intact cell wall) (Sun et al. 2017). Such reshaping of the deep-sea pliocardiine’s immune system may be an adaptation to the more intimate association between the host and the symbiont.

**Fig. 7.**
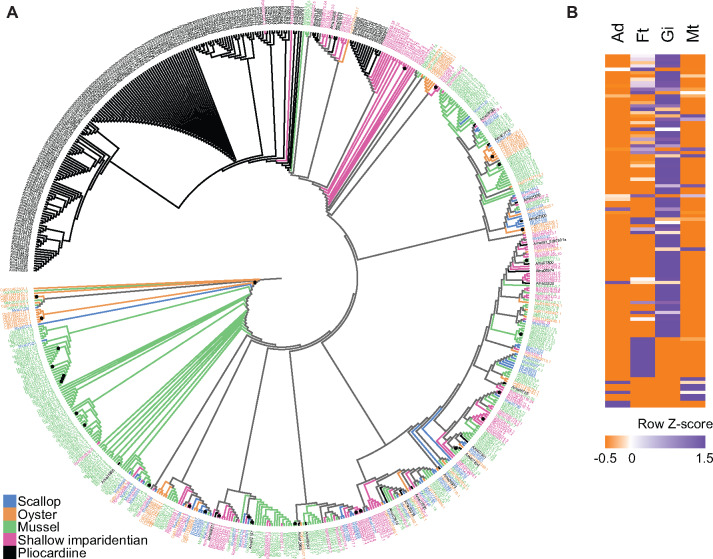
Expansion of inhibitor of apoptosis proteins (IAPs). (*A*) Maximum-likelihood tree constructed with the substitution model of VT+G4. Among the 650 IAPs found in the seven representative bivalve genomes, 204 sequences came from the pliocardiine *Archivesica marissinica* (Ama), 126 from the mussel *Gigantidas platifrons* (Gpl), 86 from the mussel *Modiolus philippinarum* (Mph); 54 from the oyster *Crassostrea gigas* (Cgi); 57 from the scallop *Mizuhopecten yessoensis* (Mye); 37 from the shallow imparidentian *Sinonovacula constricta* (Sco), and 86 from the shallow imparidentian *Ruditapes philippinarum* (Rph). (*B*) In *A. marissinica*, among the 106 IAPs (supplementary table S24, Supplementary Material online) that were transcriptionally active in four sequenced tissues (i.e., Ad, adductor muscle; Ft, foot; Gi, gill; and Mt, mantle), 85 had higher transcription in the gill. Nodes with 100 bootstrap support are labeled with a black dot. The full-size figure is deposited in Figshare.

The only significantly expanded Pfam domain in *A. marissinica* is the inhibitor of apoptosis proteins (IAPs) domain, with 204 IAP genes compared with an average of only 74 genes in other bivalves (fig. 7*A*). Since IAPs are known to suppress apoptosis and promote cell cycle progression, their expansion and high transcription in the gill tissue (fig. 7*B* andsupplementary table S24, Supplementary Material online) could indicate improved host cell survival in response to the presence of symbionts, as has been demonstrated in another symbiosis system (Kremer et al. 2012). Remarkably, this gene family is also expanded in *G. platifrons* with 126 genes, suggesting that the expansion of IAPs is a common convergent feature in deep-sea bivalves hosting chemoautotrophic symbionts.

### miRNA Prediction and miRNA–mRNA Interaction

To reveal the potential roles of miRNAs in regulating the host–symbiont relationship in *A. marissinica*, we carried out miRNA sequencing from the gill (i.e., the symbiont-harboring tissue) and the foot (i.e., nonsymbiotic tissue). In total, 86 miRNAs ranging from 19 to 24 nt in length were detected (supplementary fig. S18*A*, Supplementary Material online). This number is similar with the numbers of miRNAs reported from other molluscan genomes (Adema et al. 2017; Sun et al. 2017). Among them, eight out of nine miRNAs highly expressed in the gill (supplementary table S24, Supplementary Material online) were found to interact with 41 differentially expressed mRNAs between the gill and the foot (supplementary fig. S18*B* and table S25, Supplementary Material online). Characterization of the target mRNAs of these eight miRNAs showed that they might be involved in regulating the transcription of genes with a variety of molecular functions (supplementary fig. S18*C*, Supplementary Material online). Among these functional categories are immune defense, oxygen and inorganic carbon transportation, as well as signal transduction, which likely play a role in the interaction between *A. marissinica* and its endosymbiont (supplementary table S26, Supplementary Material online).

## Conclusions

The genome assemblies of the deep-sea vesicomyid clam *Archivesica marissinica* and its endosymbiont have provided us an opportunity to reveal key features of the symbiosis that allow vesicomyid clams to thrive in deep-sea chemosynthesis-based ecosystems. The *A. marissinica* host genome shows signatures of early genome erosion that is usually found in bacterial symbionts that recently became associated with a host, including the possession of 28 HTGs from bacterial origins, and a large number of TEs and pseudogenes whose massive expansion corresponded to the timing of the rise and subsequent divergence of symbiont-bearing vesicomyids. Analyses of the host genome have revealed its genetic integration with the bacterial symbiont genome in several aspects: 1) The expansion of gene families involved in gaseous transport from host to symbiont that support energy production and carbon fixation; 2) the contraction of gene families required for the recognition of bacterial surface features as an adaptation to the endosymbiotic relationship; 3) the expansion of apoptosis inhibitor proteins indicative of improved survival of bacteriocytes; 4) the complete loss or contraction of GHF families that process phytoplankton-derived nutrients due to heavy reliance on endosymbionts for nutrition; 5) a large number of active SLCs allowing the host–symbiont exchange of nutrients and metabolic intermediates; and 6) an active miRNA system in the host that regulates several functional categories of host genes involved in symbiosis. The endosymbiont *Candidatus* Vesicomyosocius marissinica is a thioautotrophic gammaproteobacterium. Although the symbiont genome is much reduced compared with free-living sulfur-oxidizing bacteria, it encodes complete pathways necessary for chemoautotrophic metabolism, i.e., Sox and Dsr systems, carbon fixation, and biosynthesis for 20 common amino acids, providing strong support of the host’s nutrition. Nevertheless, the symbiont has lost most regulatory genes for amino acid biosynthesis, indicating its reliance on the host for transcriptional control. Overall, analyzing the host–symbiont genomes together revealed not only their metabolic complementarity but also specific adaptations shaped through their coevolution. This first coupled clam–symbiont genome assemblies will facilitate comparative studies aiming to elucidate the diversity and evolutionary mechanisms of symbiosis allowing many invertebrates to thrive in “extreme” deep-sea chemosynthesis-based environments. Furthermore, the genomic resources generated will facilitate comparative studies aiming to understand the evolution of Mollusca, a highly diverse phylum currently underrepresented in genomic studies.

## Materials and Methods

Supplementary Material online contains details of Materials and Methods. In brief, individuals of *Archivesica marissinica* were collected from the Haima cold seep (1,361 m water depth) in the South China Sea. Genomic DNA was extracted from the foot and gill tissues of a clam, DNA libraries were constructed, sequenced on Illumina, PacBio, and ONT platforms, and assembled using several software pipelines. To annotate the genome and to examine tissue-specific gene expression, three individuals of *A. marissinica* were used for RNA extraction. For each individual, total RNA was extracted from different tissues and sequenced using the Illumina platform to obtain mRNA and miRNA data. Host genome analyses involved comparison of assembly statistics with other lophotrochozoan genomes and genomic features that may be related to symbiosis, phylogenetic relationship, and tissue-specific mRNA and miRNA expression. Symbiont genome analyses involved bench-marking with two other assembled vesicomyid symbiont genomes and determination of the major metabolic pathways. Integrated analyses of the symbiosis focused on the complementarity in host–symbiont metabolism, as well as the host’s support of the symbiosis and control of the symbiont population. 

## Supplementary Material

Supplementary data are available at *Molecular Biology and Evolution* online.

## Supplementary Material

msaa241_Supplementary_Data
