# Supplementary material for: Host–Endosymbiont Genome Integration in a Deep-Sea Chemosymbiotic Clam
Source: Mol Biol Evol. 2020 Sep 21;38(2):502–18. doi: 10.1093/molbev/msaa241 (PMC7826175; doi:10.1093/molbev/msaa241)
Supplement: msaa241_Supplementary_Data [file msaa241_supplementary_data.zip › msaa241-supplementary-data/msaa241_Supplementary_Data.docx]

**Supplementary Material**

**Host-Endosymbiont Genome Integration in a Deep-Sea Chemosymbiotic Clam**

Jack Chi-Ho Ip, Ting Xu, Jin Sun, Runsheng Li, Chong Chen, Yi Lan, Zhuang Han, Haibin Zhang, Jiangong Wei, Hongbin Wang, Jun Tao, Zongwei Cai, Pei-Yuan Qian, Jian-Wen Qiu

**This Supplementary Material includes:**

1. Materials and Methods

2. Supplementary Figs. S1 to S18 (full size PDF of Fig. 3, Fig. 7 and Fig. S6 are deposited in Figshare DOI:10.6084/m9.figshare.12198987)

3. Supplementary Tables S1, S2, S4, S5, S8, S9, S11, S13, S15, S25 and S26. Other tables (i.e., Table S3, S6, S7, S10, S12, S14, S16-S24, S27) are included in a separate Excel file

4. References

**1.** **Materials and Methods**

***Sampling***

Three specimens of *Archivesica marissinica* collected from the Haima cold seep (16°41.6561’N, 110°23.8165’E, 1361 m water depth; Yang et al. 2019) were used in this study (Supplementary Fig. S1). Specimen Ama_SCS001 was collected on 13 May 2018 by the manned submersible vehicle (MSV) *Shenhai Yongshi*, and specimens Ama_SCS002 and Ama_SCS003 were collected by the remotely operated vehicle (ROV) *Haima* on 4 May 2019. The clams were sampled using a handnet, kept in a Biobox during the dives, and immediately placed into a freezer at -80°C after the MSV or ROV arrived at the main deck of the research vessel.

***Host genome sequencing, assembly and analysis***

**Host DNA library construction and sequencing.** Genomic DNA was extracted from the foot tissue of a specimen (Ama_SCS001) using the CTAB method (Porebski et al. 1997), and purified using the Genomic DNA Clean & Concentrator kit (ZYMO Research, CA, USA). DNA quality was evaluated and quantity measured using agarose gel electrophoresis and a Qubit fluorometer (Thermo Fisher Scientific, MA, USA), respectively. High-quality DNA (OD 260/280 = 1.8-2.0, OD 260/230 = 2.0-2.2) was used for library preparation and whole genome sequencing. In brief, 1 µg DNA was used to construct a library with a 350-bp insert size using the NEBNext

DNA Library Prep Kit (New England Biolabs, MA, USA), and sequenced on an Illumina NovaSeq sequencer to generate ~ 220 Gb paired-end reads with a read length of 150 bp. In addition, 20 µg DNA was used to construct a 20-kb SMRTbell library using the SMRTbell^TM^ Template Prep Kit 1.0, and sequenced on a PacBio Sequel sequencer to generate ~ 88.2 Gb data using seven SMRT cells.

**Host genome assembly and annotation.** Adaptors and low-quality reads (quality score < 20, length < 40 bp) of the Illumina data were removed using Trimmomatic v0.38 (Bolger et al. 2014). The clean Illumina data were used to characterize the genome, which predicted the genome size to be 1.6 Gb and heterozygosity to be 0.41% at a *k*-mer size of 17 (Supplementary Fig. S2). The PacBio reads were corrected and trimmed using Canu v1.8 (Koren et al. 2017) under default settings. After filtering, a total of 1504 million Illumina clean reads (~130X genome size) and 5.0 million PacBio reads (N_50_ = 10.6 kb, ~38X genome size) were retained (Supplementary Table S1). Three bioinformatic pipelines (Supplementary material Table S2), including one using PacBio data only (wtdbg2 v2.5 + Pilon v1.22, Ruan and Li 2020) and two using both Illumina and PacBio data [i.e., Platanus v2.0.2, Kajitani et al. 2019; MaSuRCA v3.3.3 (with Flye assembler), Zimin et al. 2013], were applied to assemble the genome under default settings. A comparison of the assembly statistics (Supplementary Table S2) showed that the MaSuRCA pipeline resulted in the best assembly, with a total size (1.52 Gb) that is closest to the estimated genome size, the highest percentage of complete metazoan (odb9) Benchmarking Universal Single-Copy Orthologs (BUSCOs) (82.6%), and the smallest percentage of fragmented BUSCOs (8.7%) estimated using BUSCO v3.0.2b under default settings. The scaffolds were extended by incorporating the RNA-Seq data using AGOUTI v0.3.3 (Zhang et al. 2016) and P_RNA_scaffolder (Zhu et al. 2018) following the authors’ instructions. Microbial sequences in the clam genome were removed by search using BLASTn v2.4.0+ against the NCBI bacterial database and the symbiont genome with an E-value threshold of 1e-20.

Hi-C, a chromosome conformation capture method (Lieberman-Aiden et al. 2009), was used to further improve the genome assembly. The foot tissue dissected from the individual Ama_SCS003 was refrozen on ice and resuspended with 37% formaldehyde in serum-free Dulbecco's modified Eagle's medium (DMEM). The fixed tissue was homogenized, digested with a restriction enzyme (MBOI), labeled with a biotinylated residue, and end-repaired (Lieberman-Aiden et al. 2009). DNA was extracted and purified for library preparation with a 350-bp insert size using the NEBNext DNA Library Prep Kit (New England Biolabs, MA, USA), and sequenced on an Illumina NovaSeq sequencer to produce 184.9 Gb paired-end reads with a 150-bp read length. The raw reads were trimmed with Trimmomatic v0.38 (Bolger et al. 2014) (quality score < 20, length < 40 bp), the high quality reads were identified with HiC-Pro v2.10 (Servant et al. 2015) and the duplications were removed with the Juicer pipeline v1.5 (Durand et al. 2016) under default settings. Then, genomic scaffolding was conducted with the 3D *de novo* assembly pipeline (Dudchenko et al. 2017) under default settings for diploid genomes. Pseudo-chromosomal linkage groups were checked, and several corrections were made in Juicebox v1.11.08 to ensure that the scaffolds within the same pseudo-chromosomal linkage groups met the Hi-C linkage characteristics (Durand et al. 2016).

The genome was annotated using MAKER v3.0 (Cantarel et al. 2008) according to Sun et al. (2019). In brief, the genome was “soft-masked” using RepeatMasker v2.1 (http://www.repeatmasker.org/) with the repeat libraries of all model organisms in the RepBase version 20150807 (Bao et al. 2015), and species-specific repeat libraries in RepeatModeler v1.0.8 (Smit and Hubley 2008-2015). To provide transcriptomic evidence, RNAseq data generated from all *Archivesica marissinica* individuals were *de novo* assembled using Trinity v2.5.1 (Haas et al. 2013) under default settings. Genome-guided transcriptome was also assembled using Trinity v2.5.1 by aligning RNAseq data to the genome using histat2 v2.1.0 (Kim et al. 2015) under default settings. These two transcriptomes were merged using PASA pipeline v2.2.0 (Haas et al. 2003) following the authors’ instructions. Metazoan protein sequences from Swiss-Prot database and protein sequences for other mollusc genomes, listed in Supplementary Table S3, were used as protein evidence. Augustus v3.1 (Stanke and Morgenstern 2005) was used to predict genes in the repeat-masked genome sequences. Results from different gene predictors were integrated into a consensus weighted annotation by EVidenceModeler v1.1.1 (EVM; Haas et al. 2008) with “augustus = 5, evmprot = 7” and default settings for other parameters in the software. PASA was used to improve the EVM gene models by modifying gene structures and adding UTR annotations using the *de novo* transcriptome of three individuals (Ama_SCS001 to Ama_SCS003). The predicted genes were functionally annotated using Diamond v0.9.24 BLASTp (Buchfink et al. 2015) under the “more-sensitive” option and an E-value threshold of 1e-5 against NCBI non-redundant (nr) database downloaded on 12 April 2019. Gene functional annotation was conducted using eggNOG-mapper v2 (Huerta-Cepas et al. 2017) for Gene Ontology (GO) and Kyoto Encyclopedia of Genes and Genomes (KEGG) pathways. Signal peptides in the proteins were predicted using SingalP v4.1 (Petersen et al. 2011) under default settings.

**Phylogenetic relationships and divergence times of bivalves.** Orthologous groups (OGs) among 18 bivalve genomes (Supplementary Table S3) and a *Phreagena* *okutanii* transcriptome dataset (TSA accession number GIAT00000000; Lan et al. 2019) were inferred using Diamond v0.9.24 BLASTp implemented in OrthoFinder v2.2.7 (Emms and Kelly 2015), with the “more-sensitive” option and an E-value threshold of 1e-10 selected. An annelid genome and a brachiopod genome were used as outgraoups. Only single-copy genes with at least 70% taxon representation (i.e., at least 16 species) in OGs were used to reconstruct their phylogenetic relationships. The protein sequences were aligned using MUSCLE v3.8.31 (Edgar et al. 2004) under default settings and trimmed using TrimAL v1.3 (Capella-Gutiérrez et al. 2009) with the “-automated1” option. The aligned sequences with 357,850 distinct alignment patterns including missing sequences were concatenated for phylogenetic analysis using a maximum-likelihood method implemented in IQ-TREE v1.6.9 (Nguyen et al. 2014). The LG+F+R8 subtisition model selected by ModelFinder (Kalyaanamoorthy et al. 2017) was applied to each protein partition, and 1000 bootstrap replicates were run. Divergence times were estimated with concatenated protein sequences using MCMCtree implemented in PAML 4.8 (Yang 2007). Seven nodes were constrained by either fossil records or geological events in MCMCtree: A hard min of 47 Ma for the appearance of pliocardiines with fossil record (Amano et al. 2014); hard max of 150 Ma for *L. nyassanus* and *P. canaliculata*, corresponding to the split of South America and Africa (Hayes et al. 2009); hard min of 390 Ma for Caenogastropoda and Heterobranchia; min of 470.2 Ma and soft max of 531.5 Ma for *A. californica* and *L. gigantea* (Benton et al. 2009); and min of 532 Ma and soft max of 549 Ma for the first appearance of molluscs (Benton et al. 2015); hard min of 465.0 Ma for the first appearance of Pteriomorpha (Stöger et al. 2013); and min of 550.25 Ma and soft max of 636.1 Ma for the first appearance of Lophotrochozoa (Benton et al. 2015) (detail settings in Supplementary Fig. S3). The LG model was employed to each partition. The burn-in, sample frequency and number of samples were set as 1 million, 1000 and 10000, respectively, and MCMC for 10 million generations.

**Host gene family analysis.** Protein domains were annotated by searching the Pfam 31.0 entries (Sonnhammer et al. 1997) using the hidden 6 Markov model (HMM) (Finn et al. 2014) with an E-value threshold of 1e-4. Gene family expansion/contraction events in *A. marissinica* were determined with its Pfam domain counts compared against the background average domain counts in six bivalve genomes (i.e., *Gigantidas platifrons*, *Crassostrea gigas*, *Mizuhopecten yessoensis*, *Modiolus philippinarum*; *Ruditapes philippinarum* and *Sinonovacula constricta*) using a two-tailed Fisher’s exact test. The *p*-values were corrected using the Benjamini and Hochberg method (Benjamini and Hochberg 1995) with adjusted *p*-value < 0.05. Phylogenetic analysis of selected genes was performed using IQ-TREE v1.6.9 (Nguyen et al. 2014) with 1000 bootstrap replicates. The substitution model was selected by ModelFinder (Kalyaanamoorthy et al. 2017) implemented in IQ-TREE.

**Insertion times of transposable elements.** To understand the temporal dynamics of transposable element (TE) activities during the evolution of *A. marissinica*, the nucleotide substitution rates of bivalves were estimated using a free-ratio model implemented in the codmel script in PAML v4.8 (Yang 2007). Divergences of TEs from the consensus sequences extracted from RepeatMasker results were adjusted for multiple substitutions using the Jukes-Cantor formula K = −300/ 4× Ln (1 − D × 4/300), where D represents the distance between the fragmented repeat and the consensus sequence. Insertion times of TEs were estimated using the equation T = K/2r (Kimura 1980), where T is the insertion time, and r is the nucleotide substitution rate for each bivalve species.

**Pseudogenes.** To understand how transposable elements may have affected the genome structure of *A. marissinica*, a genome-wide pseudogene analysis was conducted for this deep-sea clam, with the shallow-water clam *Ruditapes philippinarum* included for comparison. The genes and repeat regions in the genomes were masked, and a homologous search for pseudogene candidates in the intergenic regions was conducted using tBLASTn v 2.4.0+ with E-value < 1e-20 and the SEG low-complexity filter. Candidate pseudogenes were identified using the PseudogenePipeline (https://github.com/ShiuLab/PseudogenePipeline) with the following thresholds: identity > 60%, match length > 50 amino acids, and query coverage > 70% of the query sequence (Zou et al. 2009). Putative processed pseudogenes were classified by scanning for insertion of retrotransposons on their 2 Kb flanking regions. The symbiont proteins were also searched against the *A. marissinica* genome, but no homolog of any symbiont sequence was detected. To determine pseudogene expression, RNAseq data were mapped to the genome assembly using histat2 v2.1.0 (Kim et al. 2015) under default settings. Aligned reads (with mapping quality ≥ 10) were sorted and indexed with SAMtools v1.7 (Li et al. 2009) under default settings. The read counts in each tissue were produced by runing the multicov program in BEDTools v2.24.0 (Quinlan and Hall 2010) under default parameters. Pseudogenes with read counts > 5 were considered as expressed.

**Horizontal gene transfer.** To identify genes of bacterial origin in the *A. marissinica* genome, the predicted protein sequences were searched against NCBI non-redundant database and the vesicomyid symbionts using diamond v0.9.24 BLASTp (Buchfink et al. 2015) with the “more-sensitive” option applied. For each sequence, an index of horizontal gene transfer (*h*) was calculated by subtracting the best eukaryote hit score with the best bacteria hit score, and candidate horizontally transferred genes (HTGs) were defined as those with a *h* ≥ 30 and bit score of bacterial origin ≥100 (Chen et al. 2016). To avoid analytic artefact and contamination, we applied three stringent criterions to filter the candidate HTGs (Husnik and McCutcheon 2017): 1), the presence of spliceosomal introns. This was applied because the intron splicing pathway is required in eukaryotic mRNA translation and the acquisition of spliceosomal introns is required for active transcription of HTGs after their horizontal transfer (Da Lage et al. 2013; Koutsovoulos et al. 2016). However, considering that some HTGs might have been inserted recently, we have also listed the intronless putative HTGs. 2), phylogenetic analysis of the top 25 hits for each HTG from the database search to confirm its monophyletic relationship with bacteria (Koutsovoulos et al. 2016) using the maximum likelihood method in IQ-TREE v1.6.9 (Nguyen et al. 2014), with the substitution model selected by ModelFinder (Kalyaanamoorthy et al. 2017) and ultrafast bootstrap for 1000 replications. 3), examination of paired-end Illumina reads to make sure the coverage of an HTG is similar to that of neighboring *bona fide* molluscan genes (Koutsovoulos et al. 2016; Supplementary Fig. S9). The symbiont genome was screened using the same method, but no horizontal gene transfer event from the symbiont was detected in the clam genome. To trace the evolutionary history of horizontal gene transfer in *A. marissinica*, the same HTG identification method was applied to the genomes of the shallow-water non-symbiotic clam *Ruditapes philippinarum* and the deep-sea mussel *Gigantidas platifrons* harboring methane-oxidizing symbionts. As no other vesicomyid genome is available, we searched the transcriptome of *Phreagena okutanii* (GIAT00000000), the only other vesicomyid transcriptome available for candidate HTGs. We could not rule out the possibility that the transcriptome-based HGT analysis might have underestimated the number of HTGs in *P. okutanii.*

***Symbiont population composition, genome sequencing, assembly and analysis***

**High-throughput sequencing of the bacterial 16S rRNA gene in the gill tissue.** Previous studies have found that, in some individuals of vesicomyids there are more than one phylotype of symbionts. For instance, Stewart et al. (2009) found a single symbiont strain in most “*C.” magnifica* individuals, but in some individuals two phylotypes with 93.5% identity co-occurred, with one genotype being numerically dominant (> 99.5%). To determine the symbiotic population in the individual of *A. marissinica* used for genome sequencing (Ama_SCS001), its gill tissue was used for high-throughput 16S rRNA gene sequencing using a method described in Xu et al. (2019). In brief, genomic DNA was extracted from the gill tissue, and used for paired-end metagenomic sequencing of the V3-V4 region of the 16S rRNA gene. The library was prepared using the NEBNext Ultra DNA Library Pre Kit (Illumina, CA, USA) and seuqenced on an Illumina MiSeq to obtain 250-bp sequences. After removing adapters and low-quality reads, operational taxonomic units (OTUs) were assigned using the USEARCH v11.0.667 pipeline (Edgar 2010) with a 97% similarity cut-off. All OTUs were aligned with the 16S rRNA sequences of vesicomyid symbionts using MAFFT v7.427 (Katoh and Standley 2013). The 16S V3-V4 sequences were used to construct a maximum likelihood tree with the using IQTREE v1.6.9 (Nguyen et al. 2014), based on the TN+F+G4 subtisition model selected by ModelFinder (Kalyaanamoorthy et al. 2017) and 1000 bootstrap replicates.

**Symbiont DNA library construction and sequencing.** Genomic DNA extracted from the gill tissue of Ama_SCS001 was examined for quality and quantity as described for the DNA extracted from the foot tissue. One μg of DNA was then used to construct a short-length library with a 500-bp insert size using the NEBNext DNA Library Prep Kit (New England Biolabs, MA, USA), and sequenced using an Illumina NovaSeq sequencer to generate approximate 50 Gb of 150-bp paired-end reads. In addition, 3 μg of genomic DNA was used to construct a long-length DNA library using the Ligation Sequencing Kit 1D (SQK-LSK109, ONT, Oxford, UK) according to the product’s instruction manual, and sequenced using one FLO-MIN106 R9.4 flow cell coupled to a GridION X5 sequencer (ONT, Oxford, UK). The raw reads were real-time basecalled using Guppy v1.6.0 (ONT) under default settings to generate a fastq file.

**Assembly and extraction of the symbiont genome.** The Illumina reads were filtered using Trimmomatic v0.38 (Bolger et al. 2014) to remove adaptors and low-quality reads (quality score < 20, length < 40 bp), and the coverage was normalized using the bbnorm script in bbmap v35.85 (Bushnel 2014). The ONT reads were corrected and trimmed using Canu v1.8 (Koren et al. 2017) under default settings. The clean Illumina (46,764,669 reads) and ONT sequences (20,083 reads; longest read 18.6 kb; N50 3.4 kb) were assembled using SPAdes v3.11.1 (Bankevich et al. 2012) under the “careful” option and *k*-mer sizes of 21, 33, 55, 77, 99 and 127 bp. The symbiont scaffold was determined by BLASTn v2.4.0+ against the symbiont genomes of *P. okutanii* (GenBank accession number AP009247) and “*C.” magnifica* (GenBank accession number CP000488). The completeness and potential contamination of the *A. marissinica* symbiont genome were estimated using CheckM v1.0.12 (Parks et al. 2015) under default settings.

**Symbiont genome annotation and comparison.** Coding and non-coding sequences in the *A. marissinica* symbiont genome were predicted and annotated using Prokka v1.14.4 (Seemann et al. 2014) under default settings. Pseudogenes were identified using PseudoFinder v0.11 (Syberg-Olsen and Husnik 2018) according to authors’ instructions. Functional annotation of the coding sequences was conducted by search against the databases of Clusters of Orthologous Groups (COGs), Gene Ontology (GO) and Kyoto Encyclopedia of Genes and Genomes (KEGG) using eggNOG-mapper v2 (Parks et al. 2015). The published endosymbiont genomes of *P. okutanii* and *“C.” magnifica*, and the free-living sulfur-oxidizing chemoautotroph *Thiomicrospira crunogena* (GenBank accession number CP000109) were also reanalyzed using the above pipeline. A whole genome alignment of the three vesicomyid symbiont genomes was performed using LAGAN v2.0 (Brudno et al. 2003) and Mauve v 2.4.0 (Darling et al. 2004) under default settings. Putative orthologous proteins among the three symbiont genomes were identified using OrthoFinder v2.2.7 (Emms and Kelly 2015) with Diamond v0.9.24 BLASTp (Buchfink et al. 2015) under the “more-sensitive” option and an E-value threshold of 1e-10.

***Tissue-specific host transcriptome and host/symbiont metatranscriptome***

Total RNA was extracted from the adductor muscle, foot, gill (endosymbiont-containing) and mantle tissues of two individuals (Ama_SCS001 and Ama_SCS002) using TRIzol reagent (Thermo Fisher Scientific, MA, USA). The quality of the samples was checked using 1% agarose gel electrophoresis and the quantity of RNA was determined using a NanoDrop 2000c Spectrophotometer (ThermoFisher, MA, USA). The samples were sent to Novogene Bioinformatics Technology (Beijing, China) for transcriptome library preparation and sequencing. A NEBNext Poly(A) mRNA Magnetic Isolation Module Kit (New England Biolabs, MA, USA) was used to enrich the mRNA using an oligo-dT enrichment method, and the cDNA libraries were prepared using the NEBNext Ultra RNA Library Prep Kit (New England Biolabs, MA, USA). The libraries were sequenced on an Illumina NovaSeq sequencer under the paired-end mode to produce 150-bp reads. RNAseq data for four tissues (gill, adductor muscle, mantle, foot) of another individual (Ama_SCS003) were downloaded from NCBI (SRA accession number PRJNA471131) and used in this study. The individual was collected from the same site (Lan et al. 2019). The sequencing data from the adductor muscle, mantle and foot were produced using a method identical to that used in this study. However, in order to capture both bacterial and eukaryotic sequences in the gill tissue, its cDNA library was prepared without using oligo-dT enrichment. In addition, the gene expression data of *P. okutanii* and *R. philippinarum* were obtained from Lan et al. (2019) and Mun et al. (2017), respectively. The adaptors and low-quality reads (> 10% Ns, Phred value Q ≤ 20, < 40 bp in length) were removed using Trimmomatic v0.38. Gene expression levels were normalized as TPM using Salmon v0.9.1 (Patro et al. 2017) under default settings. Genes showing tissue-specific expression pattern were determined using DESeq2 v1.22.2 (Love et al. 2014) with the DEseq normalization method, a minimum read count of 10, and paired test mode. Only genes with >2-fold expressional difference and false discovery rate (FDR; Benjamini and Hochberg method) < 0.05 were considered as having tissue-specific expression.

***MiRNA identification, target gene prediction and functional enrichment***

To determine whether microRNAs (miRNAs) are involved in regulating the symbiosis, total RNA was extracted from the gill and foot tissues of three individuals of *A. marissinica* (Ama_SCS001and Ama_SCS003) using TRIzol reagent (Thermo Fisher Scientific, MA, USA). MiRNA libraries were prepared using the NEBNext Multiplex Small RNA Library Prep Kit (Illumina, CA, USA) and sequenced on an Illumina NovaSeq platform to produce 50-bp single-end reads in Novogene (Beijing, China). The raw reads were filtered to remove low-quality reads using fastp v0.20.0 (Chen et al. 2018) under the following criteria: length_required = 18, max_length = 35, unqualified_percent_limit = 30, n_base_limit = 0. Afterwards, the *A. marissinica* genome sequences were indexed using bowtie v1.2.3 (Langmead et al. 2009). The clean miRNA reads were combined and mapped to the genome using the mapper.pl script in miRDeep2 v2.0.1.2 (Friedländer et al. 2011). MiRNAs were identified using the miRDeep2.pl script in miRDeep2 by comparing the clean reads with the known molluscan mature miRNAs in miRBase v.22.1 (Kozomara et al. 2018). Only predicted miRNAs with a miRDeep2 score ≥ 4, star (complementary) and mature read count ≥ 5, and a significant Randfold *p*-value were considered as candidate novel miRNAs in *A. marissinica*. MiRNA expression levels were quantified as the number of mature read counts in the gill and foot of the sequenced specimens using the quantifier.pl script in miRDeep2 v2.0.1.2.

The 3’ UTR sequences of the *A. marissinica* gene models were used to predict miRNA target genes via miRanda v3.3a (Enright et al. 2003) and PITA v6 (Kertesz et al. 2007) under default settings. In the miRanda analysis, only miRNA:mRNA interaction sites with a miRanda score ≥ 140, a dimer binding free energy < -5 kcalmol^-1^, and strict 5’ seed pairing were retained. In the PITA analysis, only miRNA:mRNA interaction sites with a ΔΔG < -5 kcalmol^-1^ and a seed region of 7-mer or 8-mer with no mismatches and no more than one single G:U wobble were retained. Moreover, only miRNA:mRNA interaction sites detected in both approaches were retained for downstream analysis. The read counts were normalized by DESeq2 v1.22.2 (Love et al. 2014). Tissue-specific miRNAs were determined using DESeq2 v1.22.2 (Love et al. 2014) using the paired test with a minimal DESeq normalized count of 5.

Because the main purpose of our miRNA analysis was to determine the miRNA:mRNA interaction in the gill that hosts symbionts, and in the foot that is directly exposed to the sediment, the interaction sites obtained above were further filtered by retaining only miRNAs that had mature read counts ≥ 10 in the gill and foot of the specimens, and targeted mRNAs that were expressed in either the gill or foot (TPM ≥ 0.5). Afterwards, GO functional enrichment was conducted on the retained mRNAs using the GOseq v1.39.0 under FDR < 0.05 (Young et al. 2012). The enriched GO terms were summarized using REVIGO (Supek et al. 2011) under default parameters, and representative miRNA:mRNA interaction networks of enriched GO terms were visualized using Cytoscape v3.8.0 (Shannon et al. 2003).

**2. Supplementary Figs. S1 to S18**

**
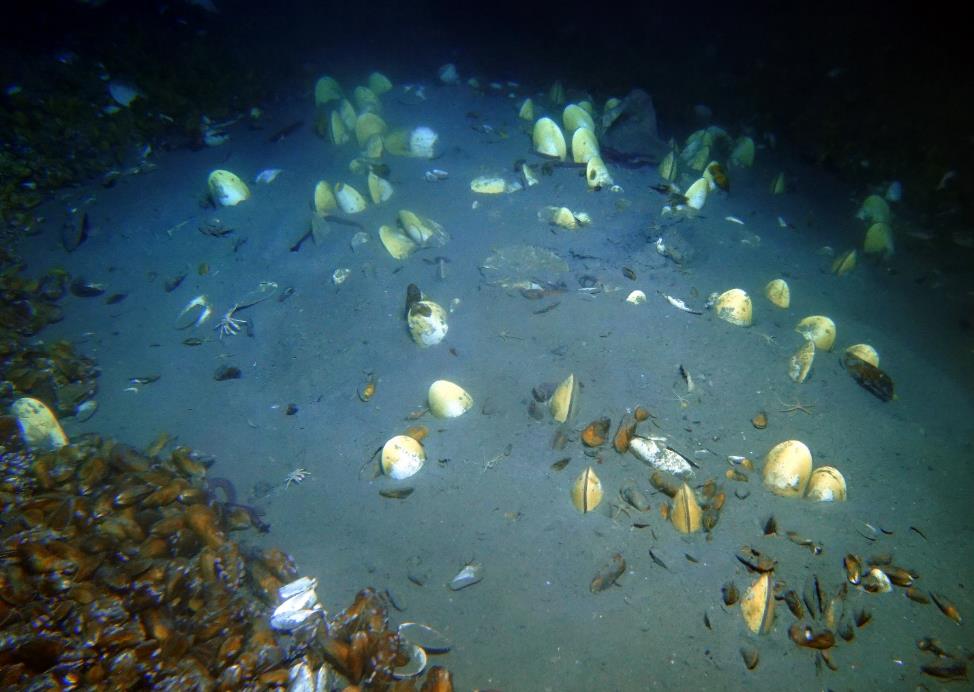
**

**Fig. S1**. A photograph taken from Haima cold seep (1361 m depth), showing individuals of the deep-sea clams *Archivessica marissinica* half buried in sediment. The clams are approximately 12 cm in shell length. Common macrobenthos co-inhabiting with the clams at the site are deep-sea mussels *Gigantidas haimaensis*. Photo credit: Jun Tao


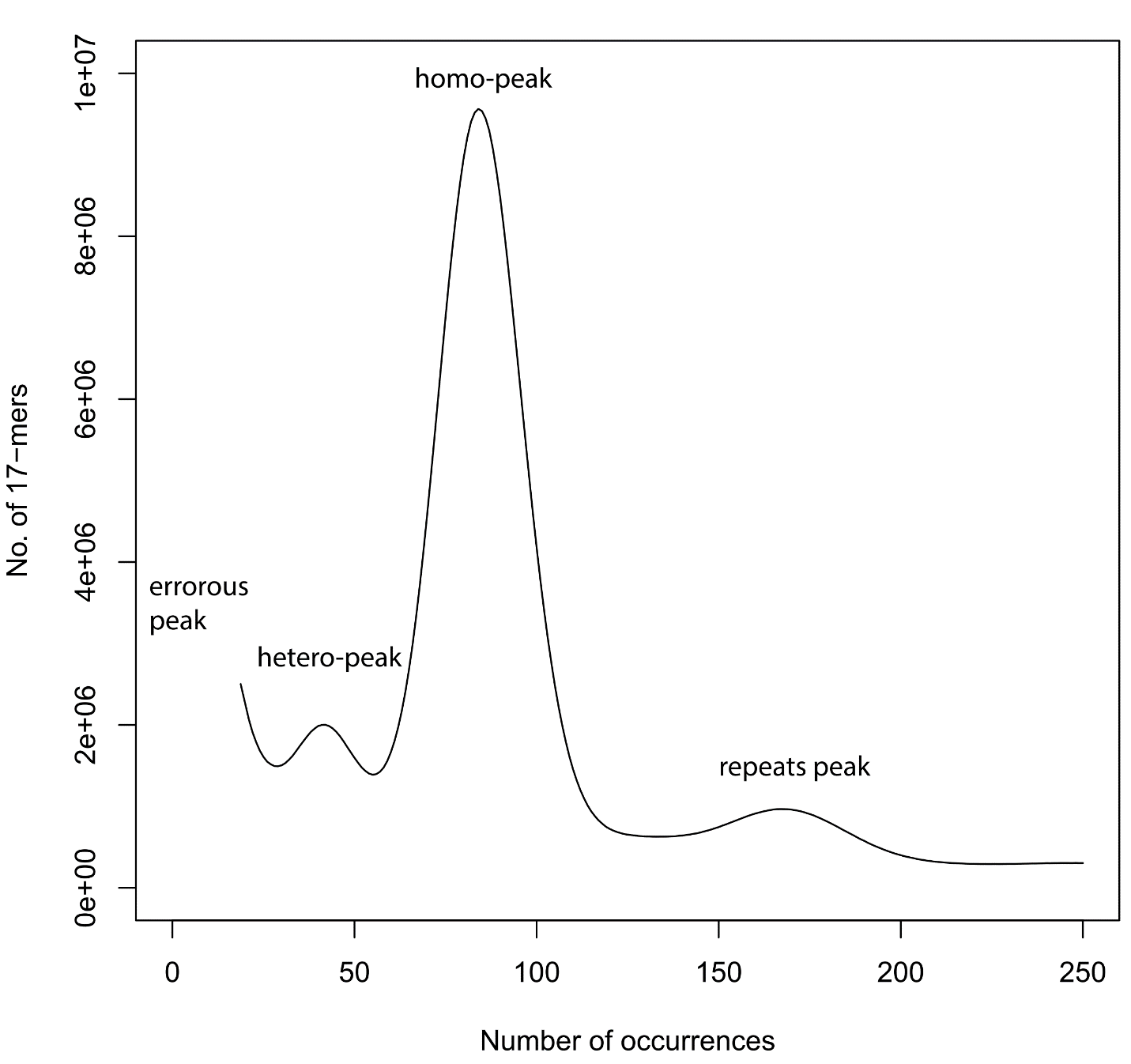


**Fig. S2**. *K*mer histogram generated using Illumina reads of *Archivesica marissinica*. Genome size of 1.6 Gb was estimted by dividing the total number of effective kmer (total number of kmer – total number of errorous kmer) by the number of homo-peak. Genome-wide heterozygosity rate of 0.41% was estimated as (hetero_*k*mers/2*k*) / (hetero_*k*mers/2 + homo_*k*mers), where *k* is the *k*mer size (Sun et al. 2017).


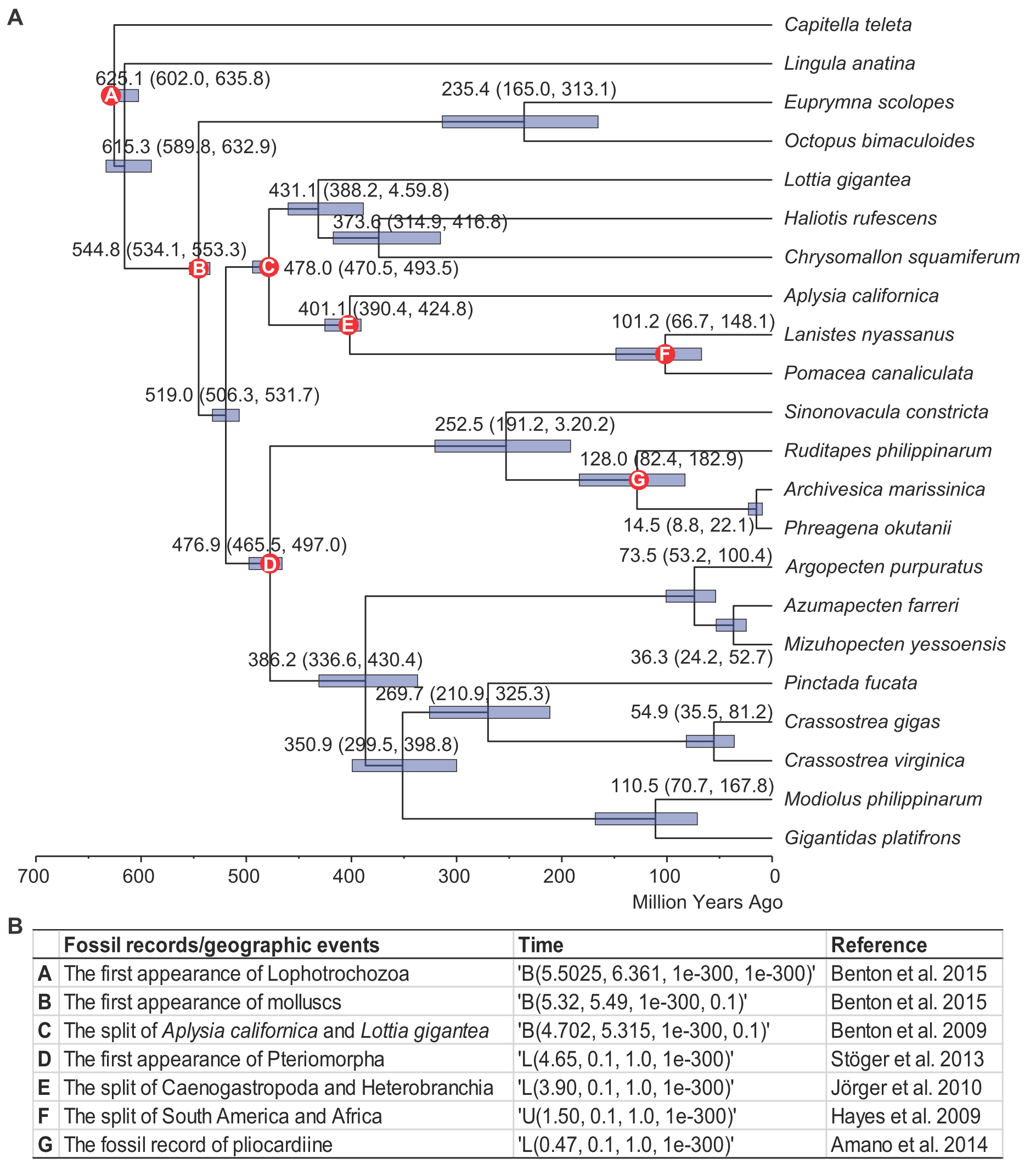


**Fig. S3.** (A) Maximum-likelihood tree constructed based on the LG+F+R8 substitution model of 22 lophotrochozoans with seven calibrated divergence time points indicating by red dots on nodes. At each node the posterior mean divergence time and upper and lower 95% confidence intervals are shown. (B) Details of calibration points based on fossil recods and geological events.


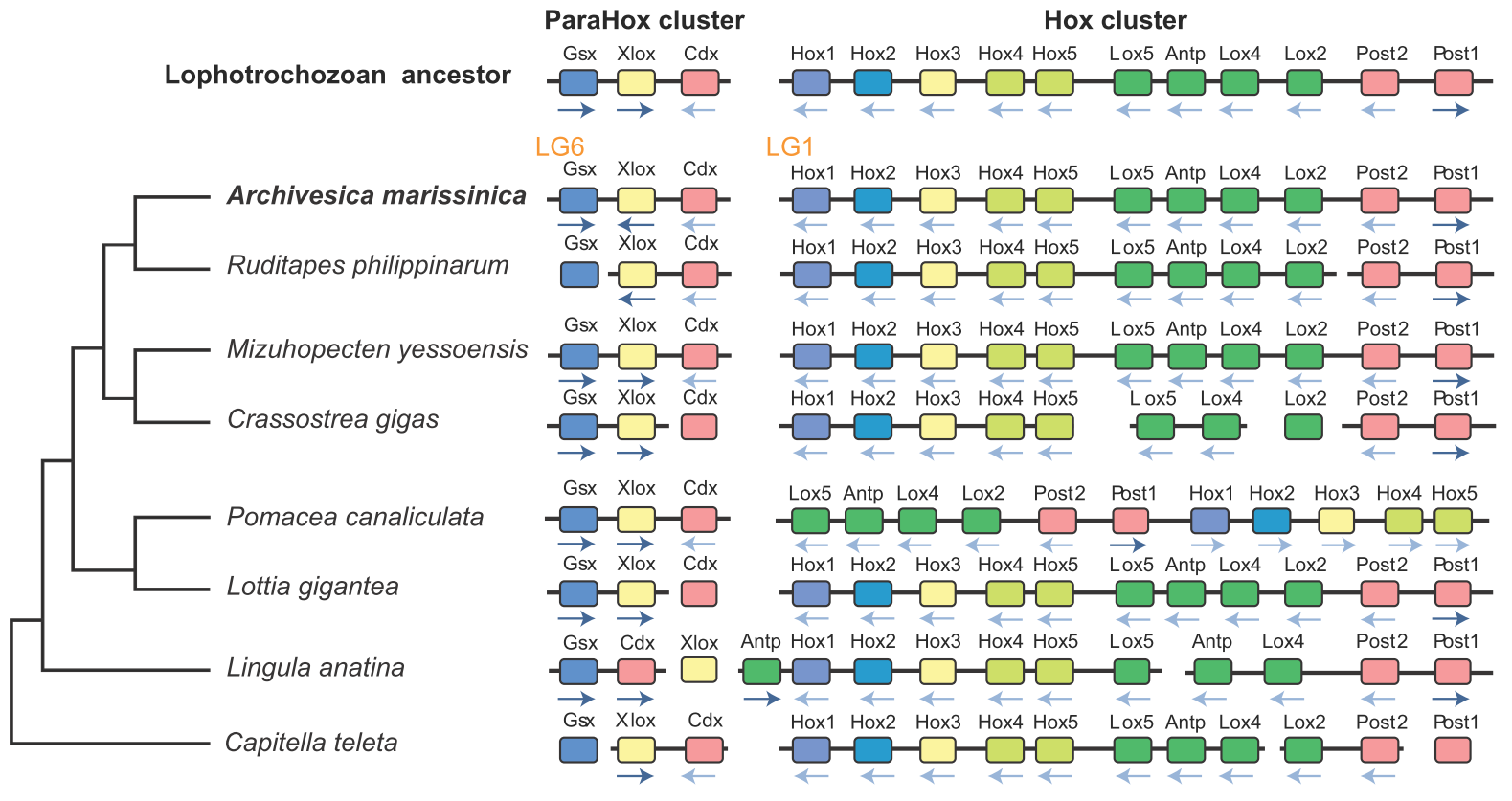


**Fig. S4.** Chromosomal organization of *Hox* and *ParaHox* genes of *Archivesica marissinica* and other lophotrochozoans. Data for non-clam genomes were adopted from Wang et al. (2017) and Sun et al. (2019). Both *Hox* and *ParaHox* genes are present as intact clusters in the deep-sea clam, identical to the ancestral state of these clusters in the presumed lophotrochozoan ancestor (top). Genes located on the same scaffold or chromosome are connected with a line, but the line length is not proportional to their sequence length. Horizontal arrows indicate transcription orientations.


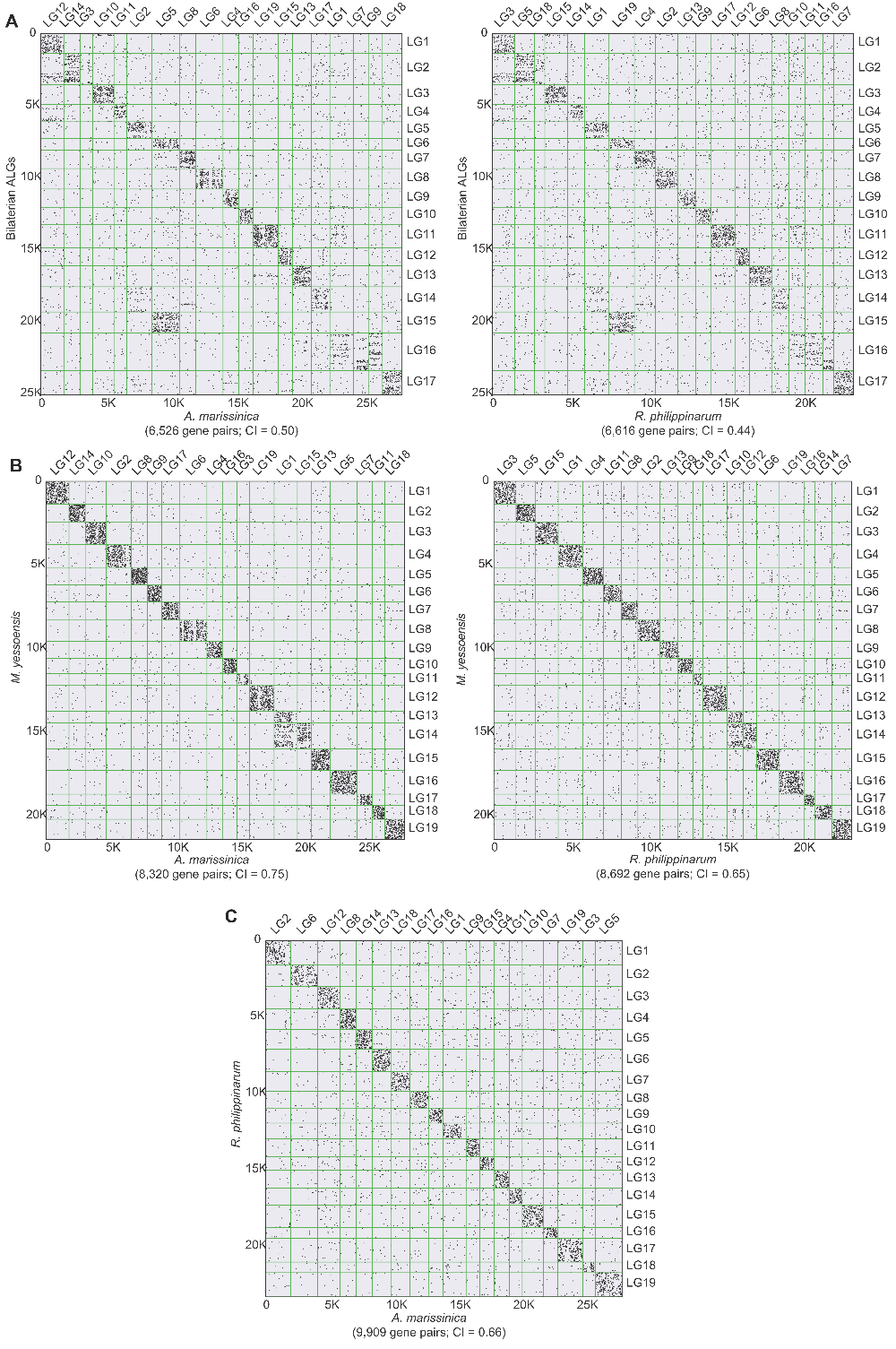


**Fig. S5.** Chromosome-scale macro-synteny comparison dot plots. (A) Between the 17 presumed bilaterian ancient linkage groups (ALGs) and the 19 linkage groups (LGs) of either *Archivesica marissinica* or *Ruditapes philippinarum*. (B) Between the 19 LGs of the scallop *Mizuhopecten yessoensis* and the 19 LGs of *A. marissinica* or *R. philippinarum*. (C) Between the 19 LGs of *A. marissinica* and the 19 LGs of *R. philippinarum*. Each dot represents the mutual protein best match between each pair of species determined by BLASTp. The genes of each species were sorted according to their genomic positions, and used for calculation of chromosome conservation index (CI) according to Simakov et al. (2013).


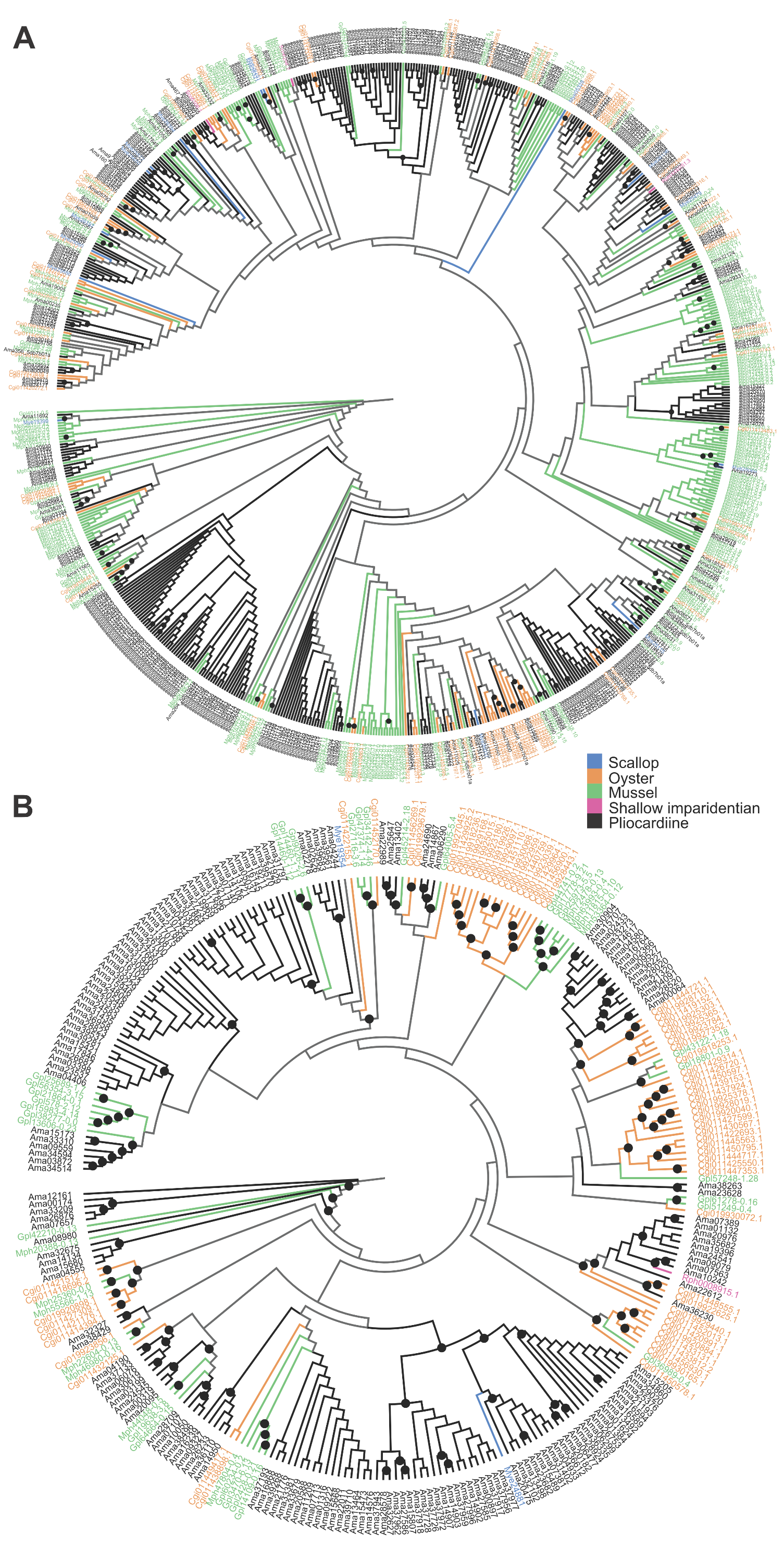


**Fig. S6.** Phylogencitc trees of expanded transposase domains. (A) PF00078 Reverse transcriptase (RNA-dependent DNA polymerase) with 408 in *A. marissinica* compared to 1 to 192 in other bivalves (substitution model: Blosum62+F+G4). (B) PF13613 Helix-turn-helix of DDE superfamily endonuclease with 181 in *A. marissinica* compared to 0 to 66 in other bivalves (substitution model: VT+F+R5). Each sequence prefix represents the bivalve species as Mye, *Mizuhopecten yessoensis* (scallop); Cgi, *Crassostrea gigas* (oyster); Gpl, *Gigantidas platifrons* (mussel); Mph, *Modiolus philippinarum* (shallow imparidentian); Rph, *Ruditapes philippinarum* (shallow imparidentian); Sco, *Sinonovacula constricta* (shallow imparidentian); Ama, *Archivesica marissinica* (pliocardiine). Nodes with 100 bootstrap support are labelled with a black dot. The full size figure is desposited on Figshare.


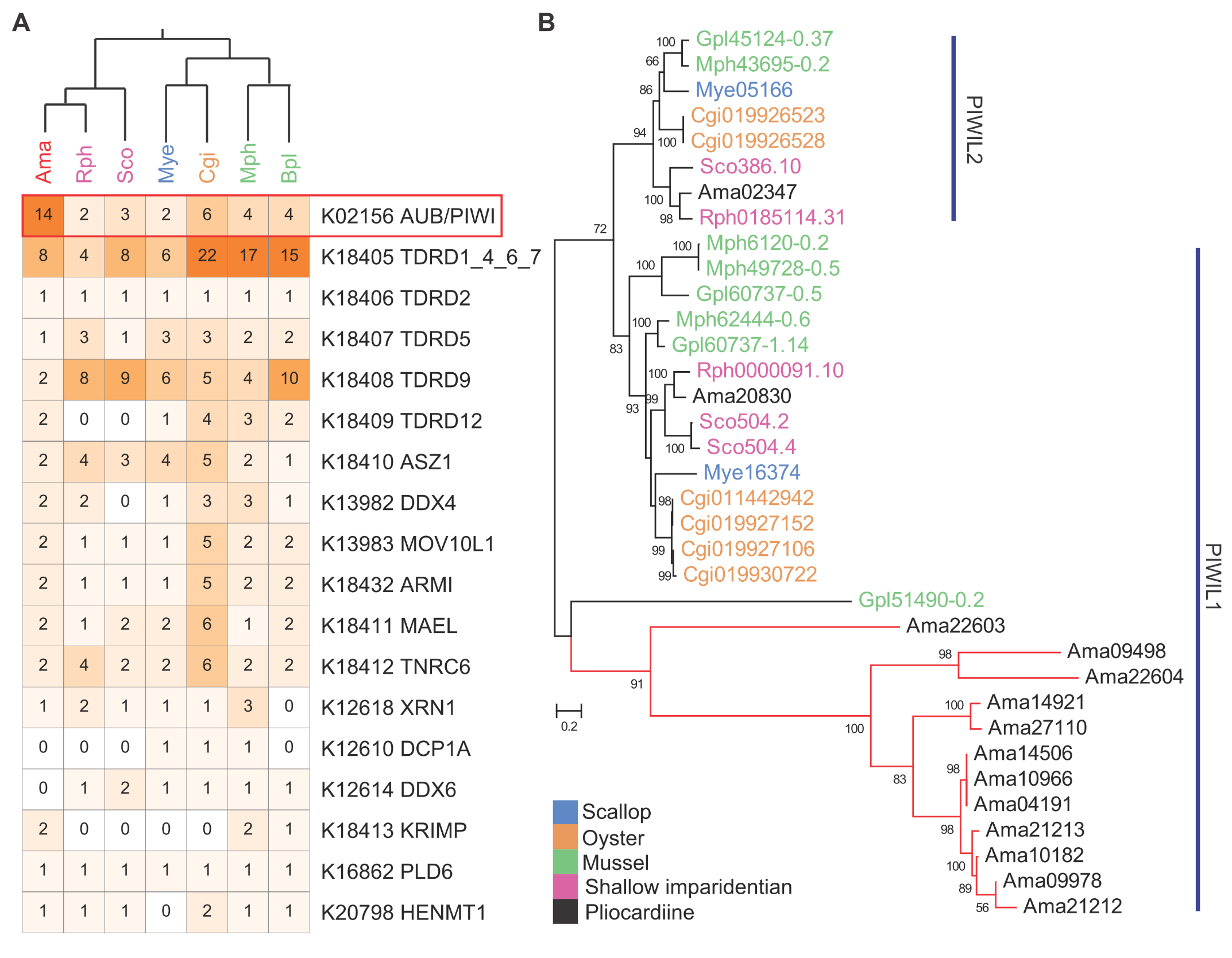


**Fig. S7.** Piwi-interacting RNA (piRNA) in *Archivesica marissinica* and six other bivalves based on KEGG annotation. (A) Number of genes involved in KEGG piRNA pathway. (B) Maximum -likelihood tree of Aubergine (K02156), a PIWI clade of proteins using the substitution model of VT+R2. It is a subfamily of Argonaute proteins that predominantly bind to mature piRNAs and direct them to bind and cleave the complementary TE mRNA (Levin and Moran 2011). The Aubergine (K02156) family, as one of the major Argonaute proteins, is enriched in *A. marissinica* with lineage specific duplication (PIWIL1) that matched with the TE expansion profiles. Mye, *Mizuhopecten yessoensis* (scallop); Cgi, *Crassostrea gigas* (oyster); Gpl, *Gigantidas platifrons* (mussel); Mph, *Modiolus philippinarum* (shallow imparidentian); Rph, *Ruditapes philippinarum* (shallow imparidentian); Sco, *Sinonovacula constricta* (shallow imparidentian); Ama, *Archivesica marissinica* (pliocardiine).


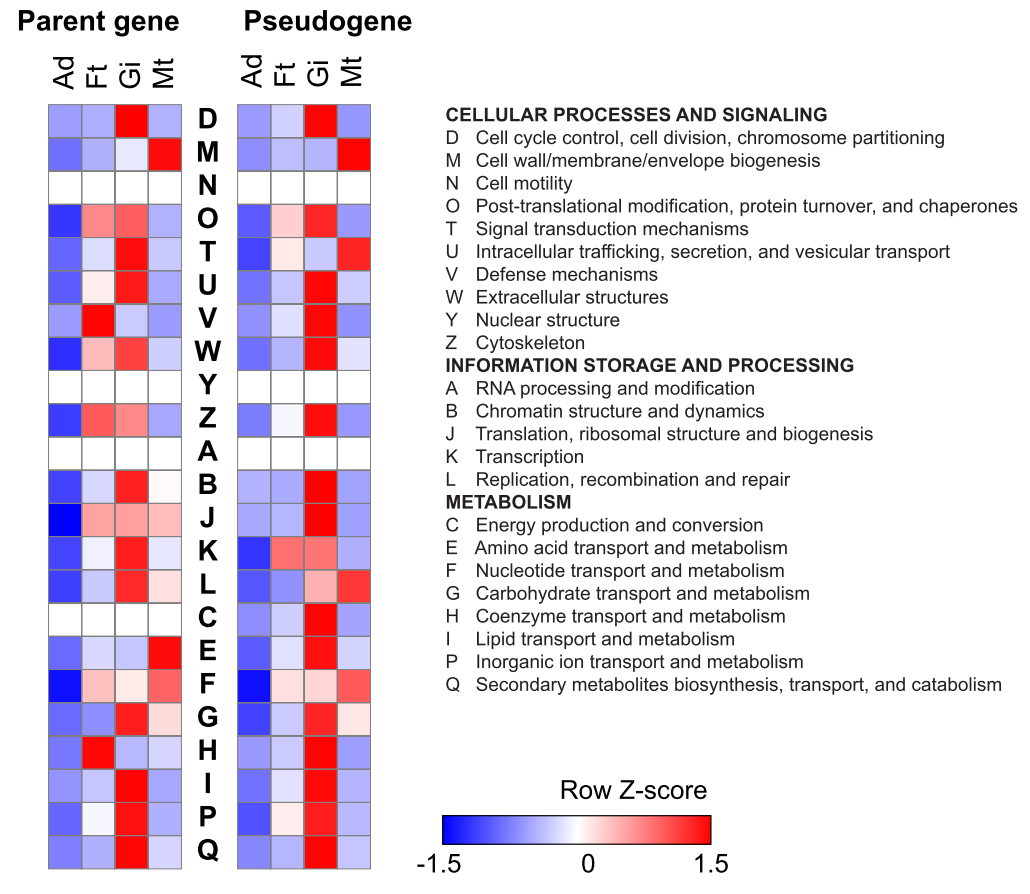


**Fig. S8.** Tissue-specific expression profile of *Archivesica marissinica* pseudogenes classified in Clusters of Orthologous Groups (COGs) functional categories. Data from four tissues (i.e., Ad, adductor muscle; Ft, foot; Gi, gill and Mt, mantle) were available for the analysis.

*h*-index

(*h* ≥ 30, best hit score of bacterial sequence ≥100)

**28,949 Transcripts**

Diamond BLASTp against NR database

**173 HTG candidates**

Intron >1

**56 HTG candidates**

Phylogenetic analysis

(monophyletic with bacterial gene)

Sequencing coverage

(10% Illumina read mapping, ≥ 5X)

Flanking with molluscan gene

**42 HTG candidates**

**28 HTG candidates**

**Ama16840**

hypothetical protein

CGI_10003494

***C. gigas***

Putative RNA-directed

DNA polymerase

from transposon BS

***C. gigas***

**Fig. S9.** A workflow for identification of horizontally transferred genes (HTGs) in *Archivesica marissinica*. Candidate genes were selected by BLASTp search and calculation of *h*-index, followed by filtering using several criteria: they must have at least one intron; they must be grouped with bacteria; they must have sequence coverage support, and they must have adjacent *bona fide* bivalve genes. On the lower right hand side of the graph is an example showing a HTG (Ama16840) flanked by two genes with homology to genes of the oyster *Crassostrea gigas*.


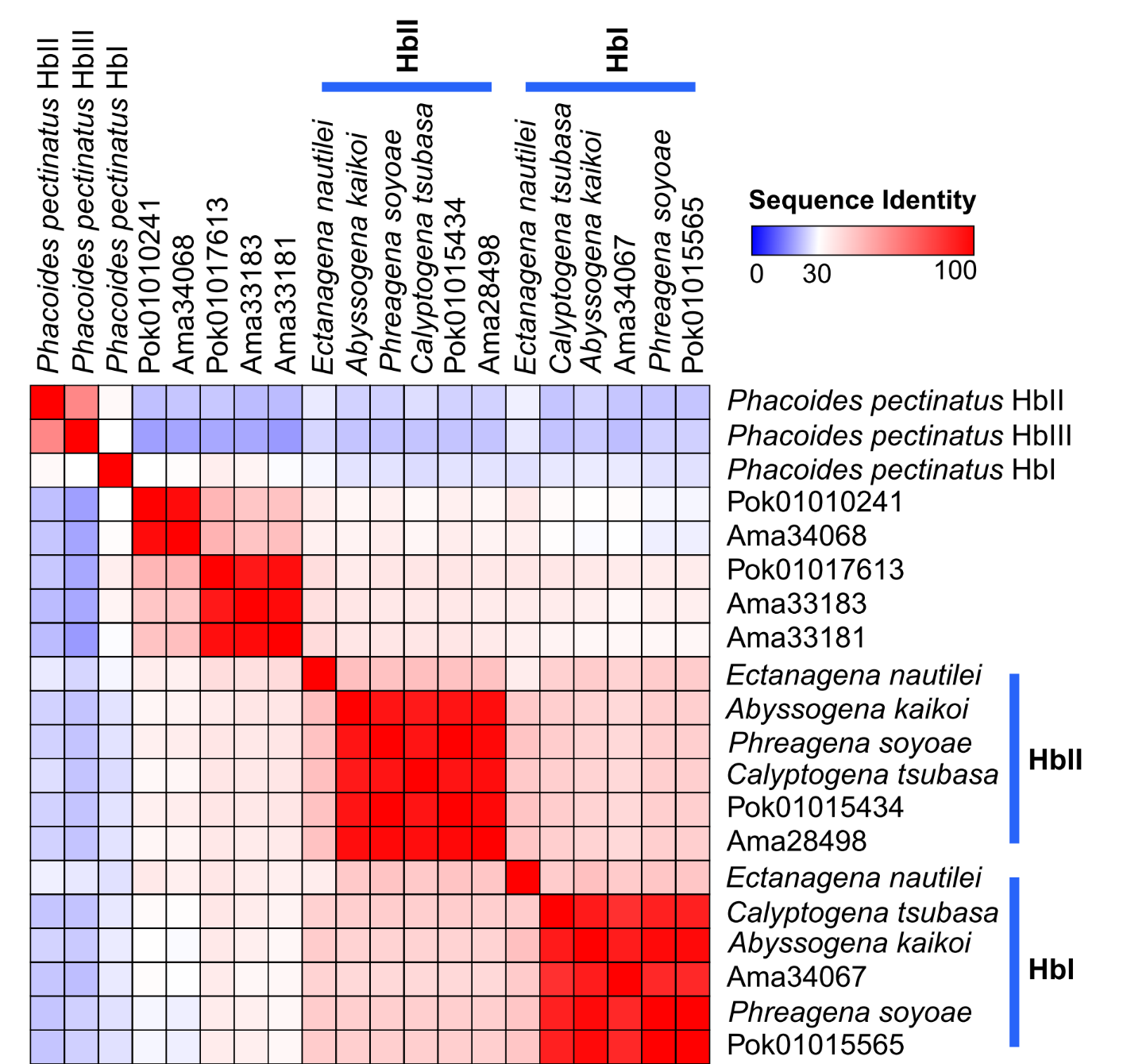


**Fig. S10.** Comparison of sequence idenitity of Group B hemoglobins (Hbs) listed in Fig. 6A. Hbs from the deep-sea vesicomyids exhibit low sequence simiality with those of the mangrove clam *Phacoides pectinatus*.


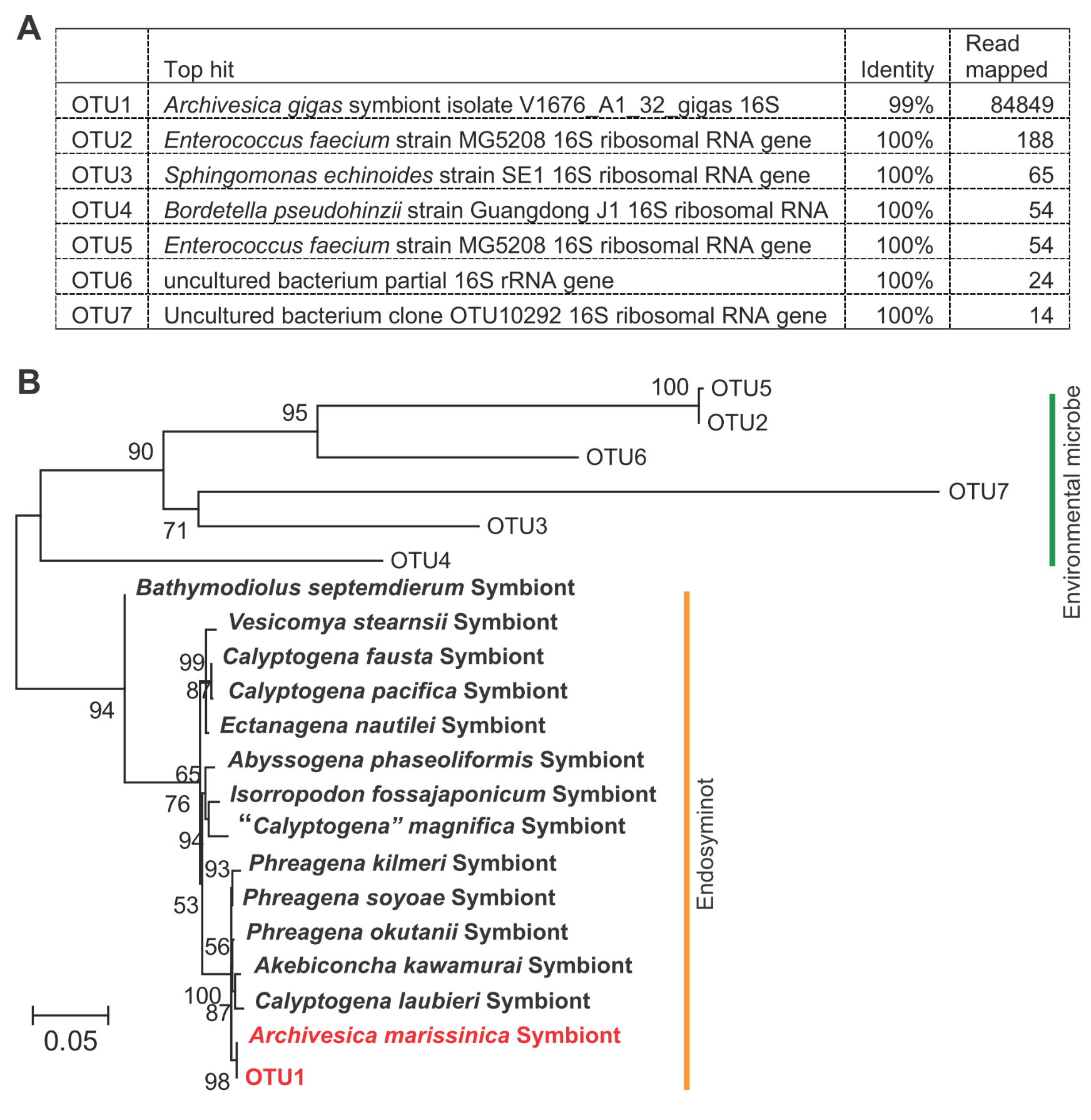


**Fig. S11**. Bacteria population structure in the *Archivesica marissinica* gill. (A) Relative abundance of operational taxonomic units (OTUs) among metagenomic reads. In total, seven OTUs were identified from around 85 thousand clean reads, with > 99.5% reads mapped OTU1. A BLASTn search against NCBI nt suggests that OTU2-OTU6 are likely environmental microbes attached to the gill surface. (B) Phylogeny of 16S rRNA V3-V4 region among the seven *A. marissinica* gill OUTs and other deep-sea vesicomyid symbionts, with the *Bathymodialus septemdierum* gill symbiont served as the outgroup. Maximum-likelihood tree was contructed with the substitution model of TN+F+G4, running with 1000 bootstrap replicates. Among these sequences, the “*Archivesica marissinica* Symbiont” sequence was obtained from the symbiont genome assembled in this study. Since OTU1 is identical to “*Archivesica marissinica* Symbiont”, the result indicated that only a single symbiont phylotype is present in the individual (Ama_SCS001) used for host and symbiont genome sequencing.


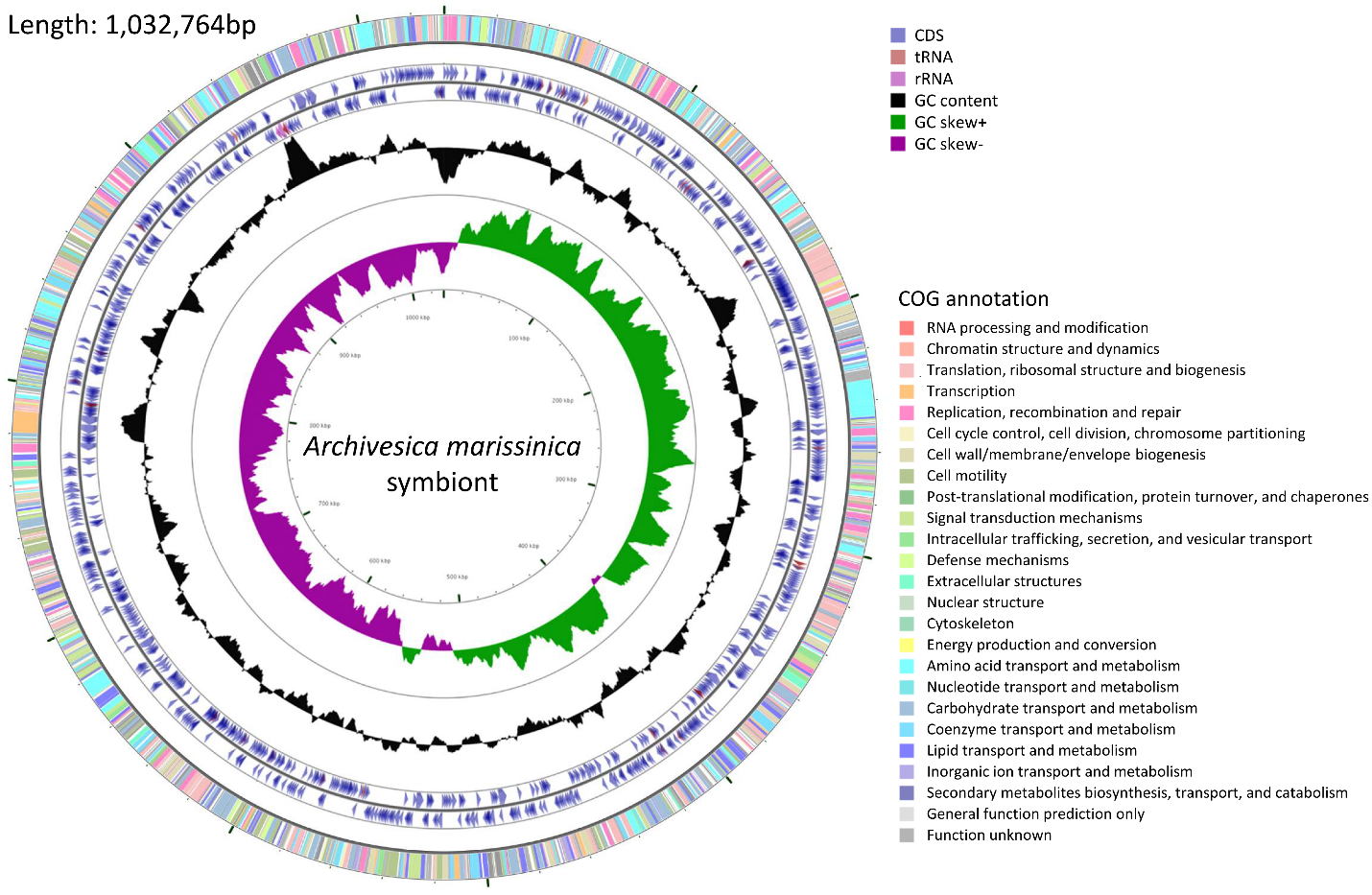


**Fig. S12.** Genome plot of the *Archivesica marissinica* symbiont made using the CGView server (Grant and Stothard 2008). From the most inner to the most outer circle: GC skew, GC content, open reading frames (CDS, tRNA and rRNA), and Clusters of Orthologous Group (COG) categories of protein-coding genes.


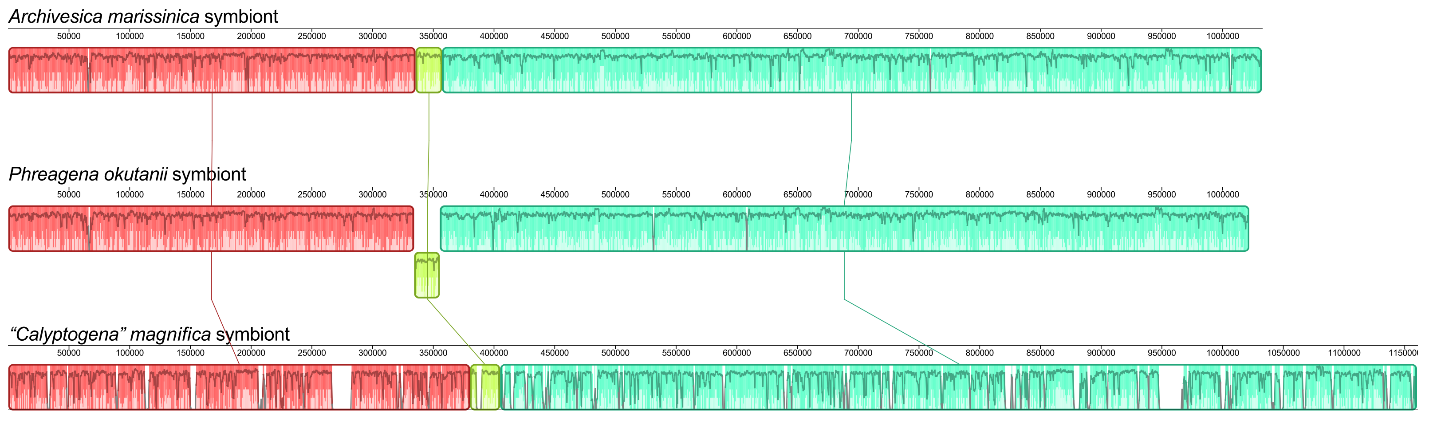


**Fig. S13.** Whole genome alignment between the *Archivesica marissinica* symbiont and other two published vesicomyid symbionts, which shows a single 21.0-Kb inversion in the *Phreagena okutanii* symbiont.


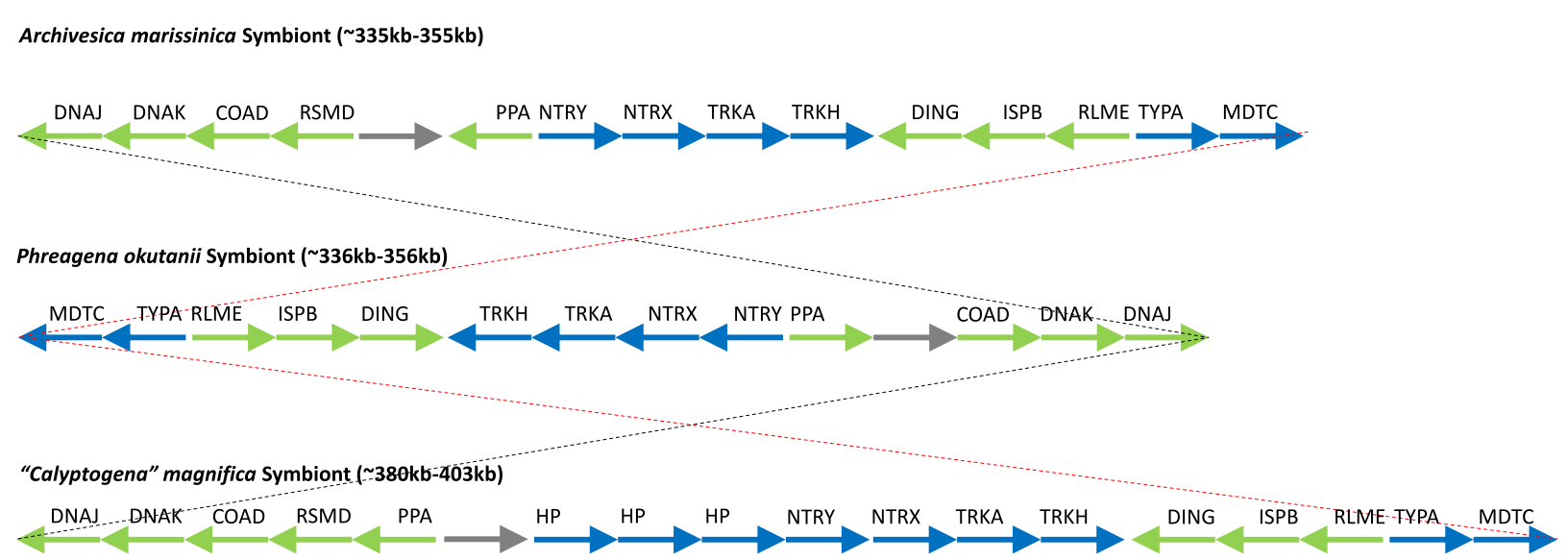


**Fig. S14.** A single inversion found in the genomic comparison between *Phreagena okutanii* symbiont and other two vesicomyid symbionts. Gray arrow indicates pseudogene. Chaperone protein (DNAJ, DNAK), phosphopantetheine adenylyltransferase (COAD), 16S rRNA (guanine(966)-N2)-methyltransferase (RSMD), inorganic pyrophosphatase (PPA), two-component system sensor (NTRX, NTRY), Trk potassium uptake system protein (TRKA, TRKH), ATP-dependent helicase (DING), octaprenyl diphosphate synthase (ISPB), 23S rRNA (uridine(2552)-2)-methyltransferase (RLMD), GTP-binding protein (TYPA), multidrug resistance protein (MDTC), and hypothetical protein (HP).


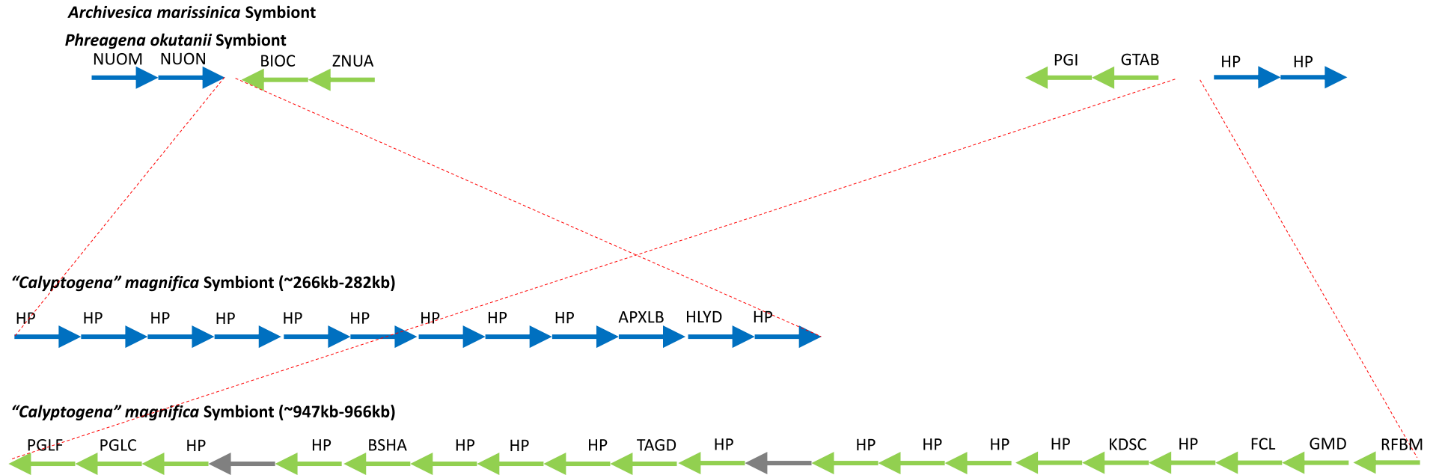


**Fig. S15.** Two unique regions in the “*Calyptogena” magnifica* symbiont. Gray arrow indicates pseudogene. Toxin RTX-I translocation ATP-binding protein (APXIB), HlyD family secretion protein (HLYD), UDP-N-acetylglucosamine 4,6-dehydratase (WBPM), UDP-N-acetyl-alpha-D-glucosamine C6 dehydratase (PGIF), undecaprenyl phosphate N,N'-diacetylbacillosamine 1-phosphate transferase (PGIC), N-acetyl-alpha-D-glucosaminyl L-malate synthase (BSHA), glycerol-3-phosphate cytidylyltransferase (TAGD), 3-deoxy-D-manno-octulosonate 8-phosphate phosphatase (KDSC), GDP-L-fucose synthetase (FCL), GDP-mannose 4,6-dehydratase (GMD), Mannose-1-phosphate guanylyltransferase (RFBM) and hypothetical protein (HP).


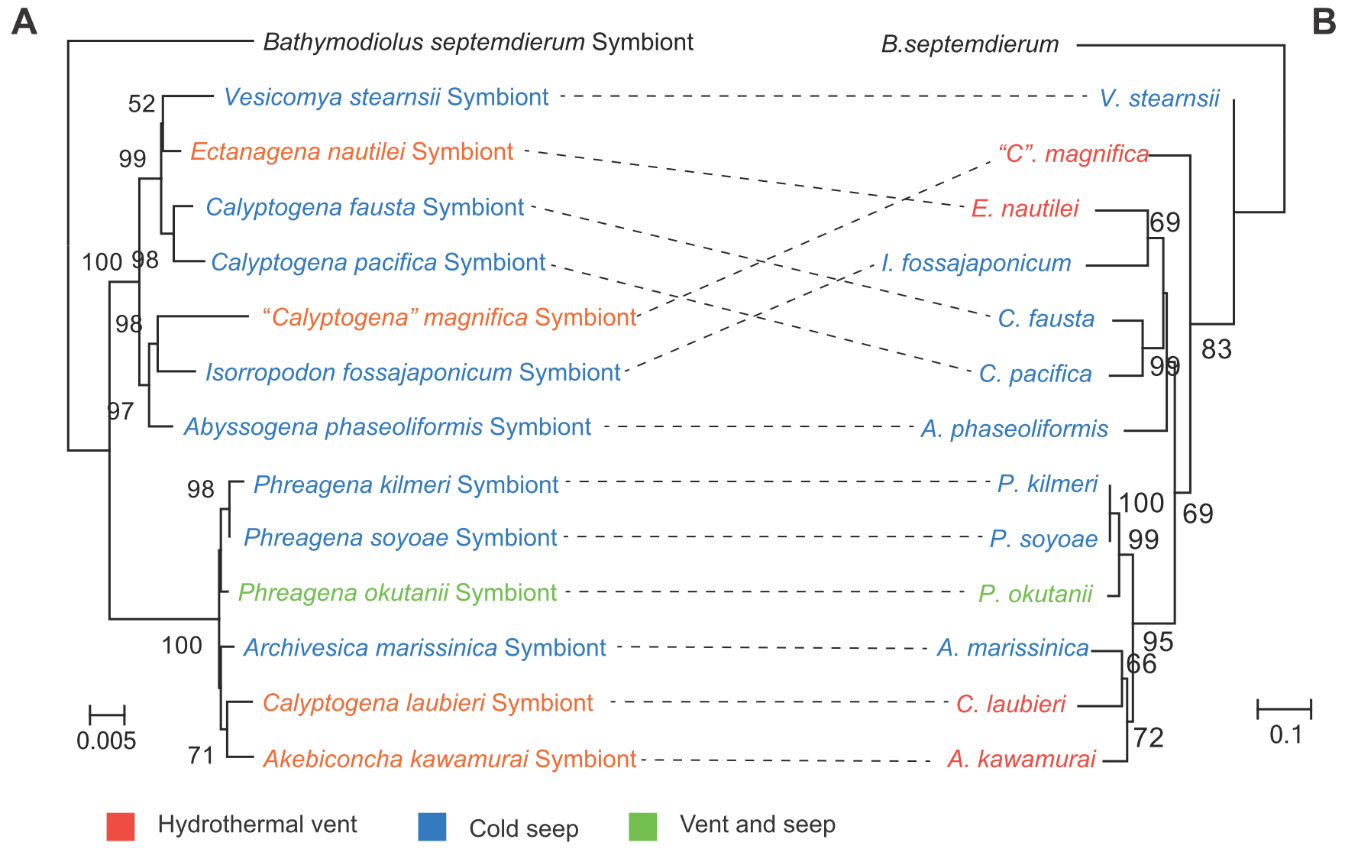


**Fig. S16.** Maximum-likelihood trees of vesicomyid clams and their symbionts. (A) a tree constructed based on concatenated 16S and 23S rRNA sequences of symbionts with the TIM2+F+R2 substitution model. (B) a tree constructed based on the cytochrome *c* oxidase subunit I gene of the clam hosts with the TN+F+G4 substitution model. Both trees were constructed using IQTREE v1.6.9 (Nguyen et al., 2014) and 1000 boostrap replicates. Numbers on nodes are bootstrap support values (> 50). The habitats of the clams are represented by different colors: red for hydrothermal vent, blue for cold seep, and green for both vent and seep. Note that *Phreagena okutaii* has been reported from both vents and seeps. Dotted lines indicate the host-symbiont relationship.


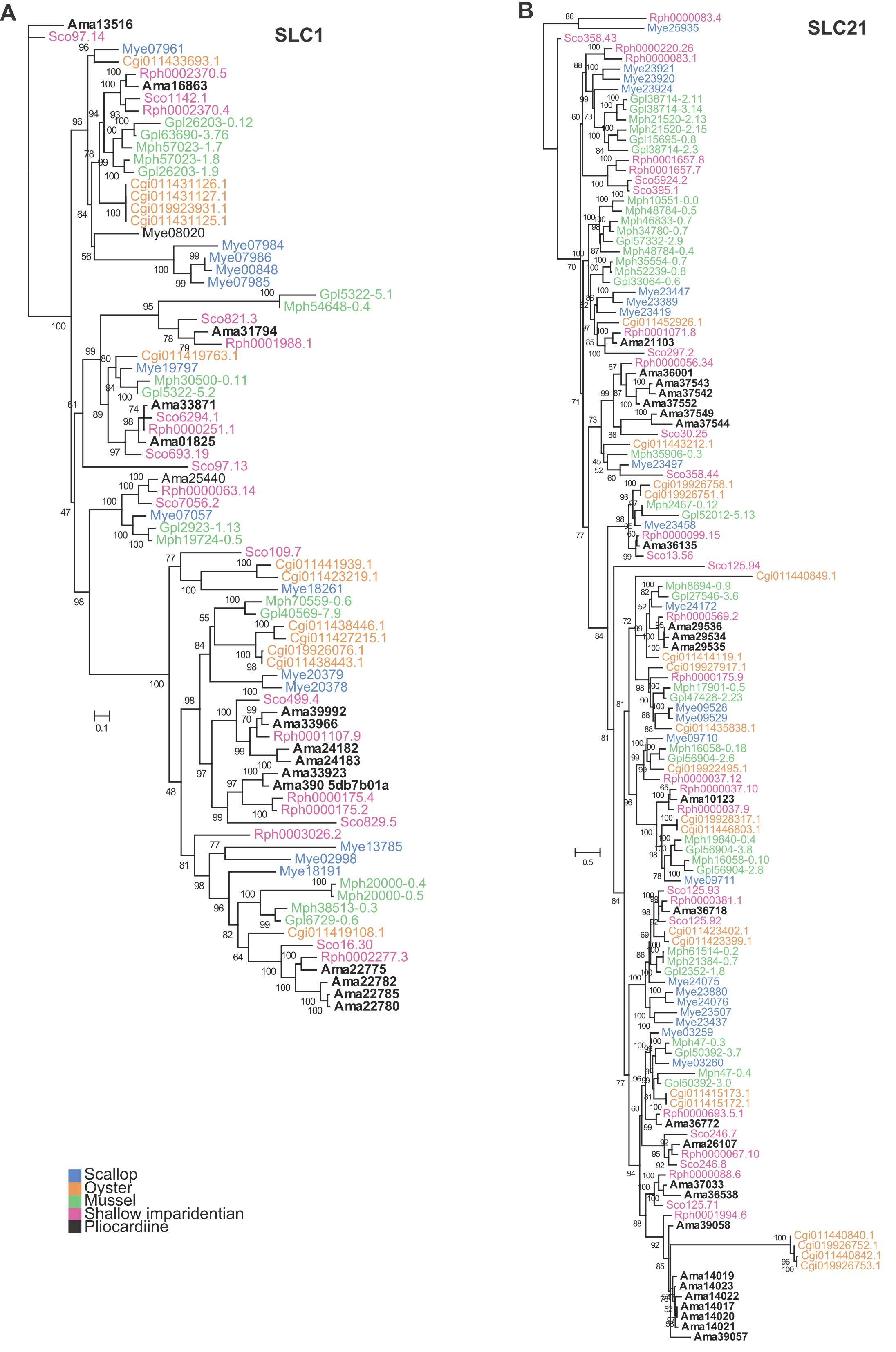


**Fig. S17**. Maximum-likelihoodtrees of (A) solute carrier families SLC1 constructed with the LG+F+R4 substitution model and (B) SLC21 constructed with the LG+F+R7 substitution model from *Archivesica marissinica* and other bivalves, with bootstrap support values >50% shown on the nodes. Sequences from *A. marissinica* are in bold type. Mye, *Mizuhopecten yessoensis* (scallop); Cgi, *Crassostrea gigas* (oyster); Gpl, *Gigantidas platifrons* (mussel); Mph, *Modiolus philippinarum* (shallow imparidentian); Rph, *Ruditapes philippinarum* (shallow imparidentian); Sco, *Sinonovacula constricta* (shallow imparidentian); Ama, *Archivesica marissinica* (pliocardiine).


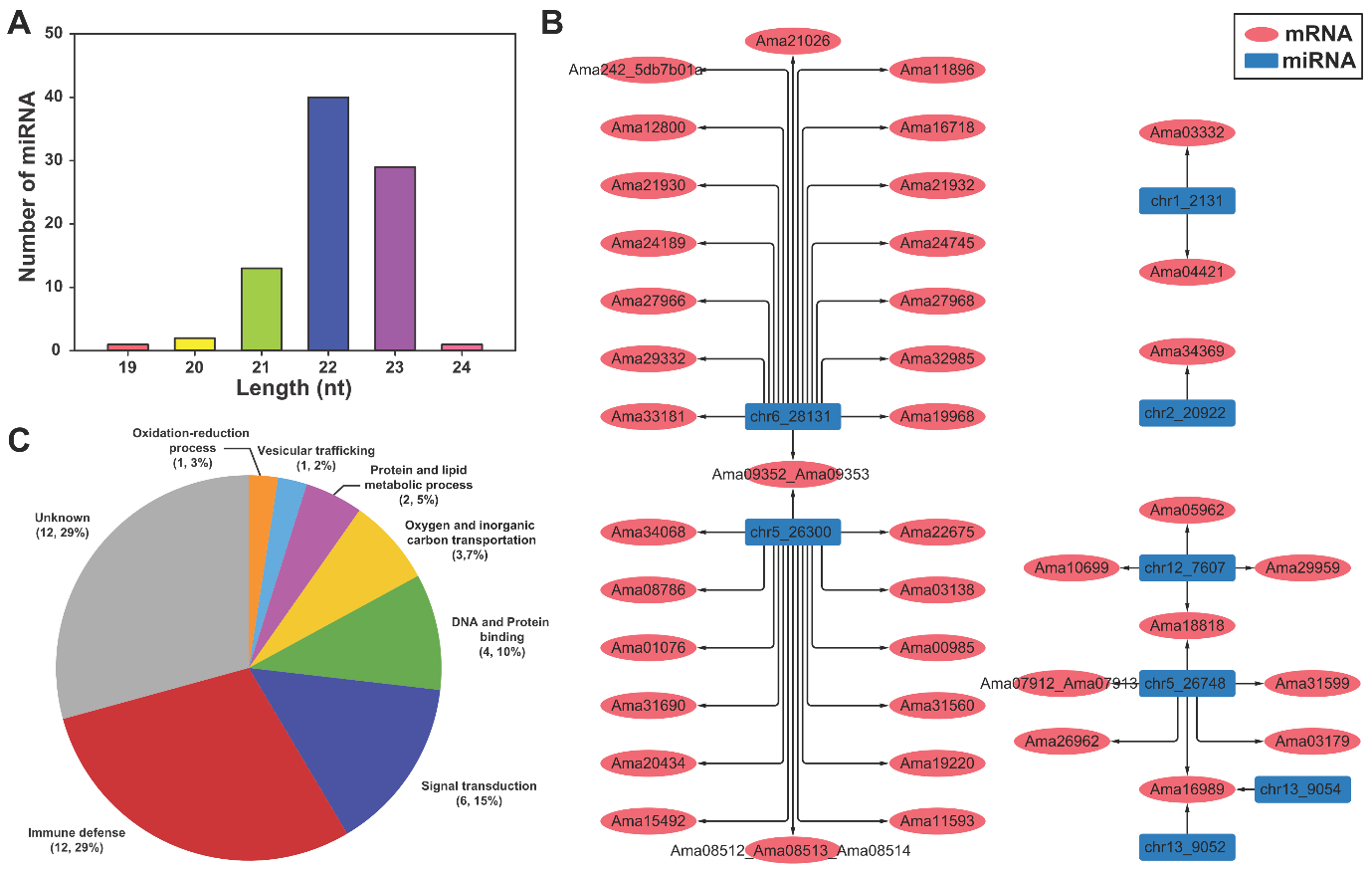


**Fig. S18.** miRNA:mRNA interaction in *Archivesica marissinica*. (A) Length distribution of 86 miRNAs; (B) miRNA:mRNA interaction network of eight highly expressed miRNAs in the gill and 41 differentially expressed mRNA targets between the gill and the foot. Expression levels of these mRNAs are summarized in Supplementary Table S27. (C) Functional characterization of the 41 mRNA targets.

**3.** **Supplementary Tables S1, S2, S4, S5, S8, S9, S11, S13, S15, S25 and S26. Other supplementary tables (i.e., Table S3, S6, S7, S10, S12, S14, S16-S24, S27)** **are included in a** **separate Excel file.**

**Table S1.** A summary of genome, mRNA, miRNA and 16S rRNA sequencing data

| **Sample** | **Library** | | **Sequencing platform** | **No. of clean/corrected reads (million)** | **Read length (bp)** |
| --- | --- | --- | --- | --- | --- |
| **Genome** | |  |  |  |  |
| Ama_SCS001-Foot | | 350 bp | NovaSeq | 1504.4 | PE150 |
|  | | 20 Kb | PacBio Sequal | 5.0 (62.6Gb) | − |
|  | |  |  |  |  |
| **Hi-C** | |  |  |  |  |
| Ama_SCS004-Foot | | Hi-C | NovaSeq | 1251.0 | PE150 |
|  | |  |  |  |  |
| **mRNAseq** | |  |  |  |  |
| Ama_SCS001-Adductor muscle | | 250~300bp | NovaSeq | 37.6 | PE150 |
| Ama_SCS001-Foot | | 250~300bp | NovaSeq | 43.4 | PE150 |
| Ama_SCS001-Gills | | 250~300bp | NovaSeq | 77.2 | PE150 |
| Ama_SCS001-Mantle | | 250~300bp | NovaSeq | 87.2 | PE150 |
| Ama_SCS002-Adductor muscle | | 250~300bp | NovaSeq | 50.2 | PE150 |
| Ama_SCS002-Foot | | 250~300bp | NovaSeq | 86.0 | PE150 |
| Ama_SCS002-Gills | | 250~300bp | NovaSeq | 71.8 | PE150 |
| Ama_SCS002-Mantle | | 250~300bp | NovaSeq | 73.8 | PE150 |
|  | |  |  |  |  |
| **miRNA** | |  |  |  |  |
| Ama_SCS001-Foot | | 18~40bp | Hiseq2500 | 21.0 | SE50 |
| Ama_SCS001-Gills | | 18~40bp | Hiseq2500 | 26.1 | SE50 |
| Ama_SCS002-Foot | | 18~40bp | Hiseq2500 | 21.1 | SE50 |
| Ama_SCS002-Gills | | 18~40bp | Hiseq2500 | 27.3 | SE50 |
| Ama_SCS003-Foot | | 18~40bp | Hiseq2500 | 25.7 | SE50 |
| Ama_SCS003-Gills | | 18~40bp | Hiseq2500 | 22.7 | SE50 |
|  | |  |  |  |  |
| **Symbiont genome** | |  |  |  |  |
| Ama_SCS001-Gills | | 500 bp | NovaSeq | 510.2 | PE150 |
|  | | Nanopore | GridIon | 0.027 (63.9Mb) | − |
|  | |  |  |  |  |
| **16S rRNA sequencing** | |  |  |  |  |
| Ama_SCS001-Gills | | <500 bp | MiSeq | 0.087 | PE250 |

**Table S2.** Assembly statistics generated by using different pipelines and steps, and evaluation of genome completeness using BUSCO analysis of 978 single-copy orthologs in metazoans (odb9). Abbreviations: C, number of complete BUSCOs; S: number of complete single copy BUSCOs; D, number of complete and duplicated BUSCOs; F, number of fragmented BUSCOs; M, number of missing BUSCOs.

| **Assembly** | **Canu+MaSuRCA (flye)** | **Canu+wtdbg2**  **+Polin X2** | **Canu+Platanus2** | **RNA_scaffold** | **Hi-C (final version)** |
| --- | --- | --- | --- | --- | --- |
| Total size (Gb) | 1.52 | 1.24 | 2.64 | 1.52 | 1.52 |
| No of scaffold | 28,581 | 22,255 | 5,141,532 | 19,871 | 4,006 |
| Scaffold N50 | 80.5 kb | 125.9 kb | 9.8 kb | 161.5 kb | 73.3 Mb |
| Scaffold L50 | 5,534 | 2,816 | 27,052 | 2,606 | 9 |
| Longest scaffold | 1.8 Mb | 1.8 Mb | 0.5 Mb | 1.3 Mb | 108.7 Mb |
| Mean size | 53.2 kb | 55.9 kb | 513 bp | 76.6 kb | 356.0 kb |
| BUSCO | C:82.6% [S:80.1%,D:2.5%], F:8.7%,M:8.7% | C:78.8% [S:77.7%,D:1.1%], F:9.4%,M:11.8% | C:77.8% [S:76.8%,D:1.0%], F:12.5%,M:9.7% | C:88.3% [S:86.4%,D:1.9%], F:5.1%,M:6.6% | C:91.8% [S:90.1%,D:1.7%], F:1.7%,M:6.5% |

**Table S4.** Read mapping statistics in *Archivesica marissinica.*

| **Data** | **Clean reads/contigs** | **Mapping rate** |
| --- | --- | --- |
| Illumina read (subsampled) | 151,171,779 | 99.14% |
| PacBio read | 28,988,990 | 99.74% |
|  |  |  |
| *De novo* transcriptome (GIAS00000000; Lan et al. 2019) | 25,089 | 92.93% |
|  |  |  |
| RNAseq |  |  |
| Ama_SCS001-Adductor muscle | 31,327,145 | 77.24% |
| Ama_SCS001-Foot | 33,755,762 | 87.07% |
| Ama_SCS001-Gills | 30,353,800 | 81.81% |
| Ama_SCS001-Mantle | 43,017,892 | 90.26% |
| Ama_SCS002-Adductor muscle | 13,916,566 | 71.75% |
| Ama_SCS002-Foot | 25,143,466 | 81.46% |
| Ama_SCS002-Gills | 4,905,857 | 66.39% |
| Ama_SCS002-Mantle | 10,119,000 | 85.24% |
| Ama_SCS003-Adductor muscle | 10,415,670 | 78.41% |
| Ama_SCS003-Foot | 20,461,766 | 86.50% |
| Ama_SCS003-Gills | 24,460,146 | 90.18% |
| Ama_SCS003-Mantle | 15,887,820 | 79.72% |

**Table S5.** Comparison of genome assembly characteristics, protein coding genes and pseudogenes between *Archivesica marissinica* and *Ruditapes philippinarum.*

| **Items** | ***Archivesica marissinica*** | ***Ruditapes philippinarum*** |
| --- | --- | --- |
| Assembled genome | 1524.8 Mb | 1129.1 Mb |
| Scaffold N50 | 73.3 Mb | 56.5 Mb |
| LG1 | 99.3 Mb | 61.9 Mb |
| LG2 | 97.9 Mb | 55.1 Mb |
| LG3 | 52.9 Mb | 57.6 Mb |
| LG4 | 62 Mb | 58.5 Mb |
| LG5 | 108.7 Mb | 58.3 Mb |
| LG6 | 100.7 Mb | 56.8 Mb |
| LG7 | 60.2 Mb | 49.2 Mb |
| LG8 | 70.1 Mb | 47.6 Mb |
| LG9 | 60.2 Mb | 39.6 Mb |
| LG10 | 75.2 Mb | 42.1 Mb |
| LG11 | 46.6 Mb | 46.1 Mb |
| LG12 | 91.9 Mb | 36.2 Mb |
| LG13 | 72.1 Mb | 47.3 Mb |
| LG14 | 71.8 Mb | 39.8 Mb |
| LG15 | 56.1 Mb | 51 Mb |
| LG16 | 61.6 Mb | 32.6 Mb |
| LG17 | 81.5 Mb | 56.5 Mb |
| LG18 | 73.3 Mb | 26 Mb |
| LG19 | 96.2 Mb | 62.1 Mb |
| Unplaced scaffolds | 86.7Mb | 204.6 Mb |
|  |  |  |
| **Protein-coding genes** | 28,949 | 27,652 |
| With annotation | 25,426 | -- |
| NCBI Nr | 25,111 | -- |
| Pfam domain | 17,064 | -- |
| GO | 9,927 | -- |
| KEGG | 6,581 | -- |
| SignalP4.0 | 1,535 | -- |
|  |  |  |
| **Pseudogene** | 10,211 | 2,015 |
| Parent gene | 2,528 | 1,078 |
| Parent gene with COGs | 824 (32.6%) | 392 (36.4%) |
| maximum pseudogene per parent gene | 190 | 38 |
| Pseudogene with COGs (annotated by parent gene) | 2,599 (25.5%) | 744 (36.9%) |
| Pseudogene with expression (read counts >5) | 4,616 (45.3%) | -- |
|  |  |  |
| Putative processed pseudogene (%) | 7,509 (73.5%) | 763 (37.9%) |
| Top three retrotransposon (involved pseudogene; median insertion time) | SINE/tRNA-Deu-L2  (3,719; 50Ma) | LINE/RTE-X  (153; >150Ma) |
|  | LTR/Ngaro  (1,935; 65Ma) | LTR/Gypsy  (116; >150Ma) |
|  | LINE/RTE-X  (1,527; 135Ma) | SINE/tRNA  (114; 110Ma) |

**Table S8**. Genome properties of three vesicomyid symbionts and a free-living sulfur-oxidizing bacteria.

|  | ***Ca.* V. marissinica** | ***Ca.* V. okutanii** | ***Ca.* R. magnifica** | ***Thiomicrospira crunogena*** |
| --- | --- | --- | --- | --- |
| GenBank No. | CP054877 | AP009247 | CP000488 | CP000109 |
| Lifestyle | Endosymbiont | Endosymbiont | Endosymbiont | Free-living |
| Genome size (bp) | 1,032,764 | 1,022,154 | 1,160,782 | 2,427,734 |
| GC content (%) | 31.2 | 31.6 | 34.0 | 43.1 |
| CheckM completeness (%) | 94.84 | 93.54 | 94.16 | 99.39 |
| Genes | 1,021 | 1,033 | 1,262 | 2,338 |
| Average gene length (bp) | 876.7 | 869.8 | 816.1 | 979.7 |
| Protein coding genes | 935 | 946 | 1,052 | 2,246 |
| Coding regions (%) | 91.6 | 91.6 | 83.4 | 96.1 |
| Pseudogene | 47 | 48 | 171 | 40 |
| rRNA | 3 | 3 | 3 | 9 |
| tRNA | 36 | 36 | 36 | 43 |
| Mobile element | 0 | 0 | 0 | 10 |
| Conserved regions to *Ca.* V. marissinica (%)^#^ | *--* | 96.5 | 82.2 | 1.50 |

^#^Pair-wise whole genome alignment between *Ca.* V. marissinica (query genome) and other related bacterial genomes using LAGAN.

**Table S9.** Clusters of Orthologous Groups (COGs) categories of three vesicomyid symbionts and a free-living sulfur-oxidizing bacteria. The value in each category indicates the number of genes in the category with the percentage among the total protein coding genes in brackets.

| **COGs** | **Description** | ***Ca.* V. marissinica** | ***Ca.* V. okutanii** | ***Ca.* R. magnifica** | ***Thiomicrospira crunogena*** |
| --- | --- | --- | --- | --- | --- |
| **Cellular processes and signaling** | |  |  |  |  |
| D | Cell cycle control, cell division, chromosome partitioning | 10 (1.0) | 10 (1.0) | 12 (1.0) | 24 (1.0) |
| M | Cell wall/membrane/envelope biogenesis | 53 (5.3) | 54 (5.4) | 76 (6.1) | 140 (6.0) |
| N | Cell motility | 0 (0) | 0 (0) | 0 (0) | 56 (2.4) |
| O | Post-translational modification, protein turnover, and chaperones | 67 (6.8) | 66 (6.6) | 73 (5.9) | 116 (5) |
| T | Signal transduction mechanisms | 13 (1.3) | 13 (1.3) | 14 (1.1) | 144 (6.2) |
| U | Intracellular trafficking, secretion, and vesicular transport | 17 (1.7) | 19 (1.9) | 20 (1.6) | 52 (2.2) |
| V | Defense mechanisms | 7 (0.7) | 7 (0.7) | 8 (0.6) | 23 (1.0) |
| **Information storage and processing** | |  |  |  |  |
| A | RNA processing and modification | 1 (0.1) | 1 (0.1) | 1 (0.1) | 1 (0.0) |
| B | Chromatin structure and dynamics | 0 (0) | 0 (0) | 0 (0) | 2 (0.1) |
| J | Translation, ribosomal structure and biogenesis | 129 (13.0) | 129 (13.0) | 130 (10.5) | 155 (6.7) |
| K | Transcription | 22 (2.2) | 22 (2.2) | 25 (2.0) | 70 (3.0) |
| L | Replication, recombination and repair | 50 (5.0) | 48 (4.8) | 53 (4.3) | 111 (4.8) |
| **Metabolism** | |  |  |  |  |
| C | Energy production and conversion | 85 (8.6) | 85 (8.6) | 85 (6.8) | 105 (4.5) |
| E | Amino acid transport and metabolism | 91 (9.2) | 91 (9.2) | 96 (7.7) | 146 (6.3) |
| F | Nucleotide transport and metabolism | 45 (4.5) | 45 (4.5) | 46 (3.7) | 57 (2.5) |
| G | Carbohydrate transport and metabolism | 22 (2.2) | 22 (2.2) | 28 (2.3) | 66 (2.8) |
| H | Coenzyme transport and metabolism | 84 (8.5) | 84 (8.5) | 81 (6.5) | 94 (4.1) |
| I | Lipid transport and metabolism | 32 (3.2) | 32 (3.2) | 32 (2.6) | 41 (1.8) |
| P | Inorganic ion transport and metabolism | 43 (4.3) | 43 (4.3) | 43 (3.5) | 149 (6.4) |
| Q | Secondary metabolites biosynthesis, transport, and catabolism | 4 (0.4) | 4 (0.4) | 6 (0.5) | 24 (1.0) |
| **Poorly characterized** | |  |  |  |  |
| S | Function unknown | 125 (12.6) | 122 (12.3) | 124 (10.0) | 514 (22.2) |
| **Not in COGs** | | 92 (9.3) | 96 (9.7) | 289 (23.3) | 229 (9.9) |

**Table S11.** A list of the sulfur oxidation-related genes found in three vesicomyid symbiont genomes. Gene expression data are presented for *Ca.* V. marissinica. Gene ID prediction was based on Harada et al. (2009).

| **Gene ID#** | **Gene function** | **Expression in *Ca.* V. marissinica (TPM)** | ***Ca.* V. marissinica** | ***Ca.* V. okutanii** | ***Ca.* R. magnifica** |
| --- | --- | --- | --- | --- | --- |
| *aprA* | Adenylylsulfate reductase alpha subunit | 12186.93 | Vma00092 | Vok00096 | Rma00102 |
| *aprB* | Adenylylsulfate reductase beta subunit | 15798.33 | Vma00091 | Vok00095 | Rma00101 |
| *aprM* | Adenylylsulfate reductase membrane anchor | 5846.94 | Vma00090 | Vok00094 | Rma00100 |
| *dsrA* | dissimilatory sulfite reductase alpha subunit | 23725.31 | Vma00818 | Vok00826 | Rma00940 |
| *dsrB* | dissimilatory sulfite reductase beta subunit | 11875.49 | Vma00817 | Vok00825 | Rma00939 |
| *dsrC1* | DsrC-like protein | 4311.07 | Vma00813 | Vok00821 | Rma00935 |
| *dsrC3* | DsrC-like protein | 2479.18 | Vma00584 | Vok00596 | Rma00681 |
| *dsrE* | Intracellular sulfur oxidation protein DsrE | 4357.23 | Vma00816 | Vok00824 | Rma00938 |
| *dsrF* | Intracellular sulfur oxidation protein DsrF | 3424.78 | Vma00815 | Vok00823 | Rma00937 |
| *dsrJ* | Intracellular sulfur oxidation protein DsrJ | 611.49 | Vma00809 | Vok00817 | Rma00931 |
| *dsrK* | Intracellular sulfur oxidation protein DsrK | 953.97 | Vma00811 | Vok00819 | Rma00933 |
| *dsrL* | Intracellular sulfur oxidation protein DsrL | 3702.88 | Vma00810 | Vok00818 | Rma00932 |
| *dsrM* | Intracellular sulfur oxidation protein DsrM | 426.31 | Vma00812 | Vok00820 | Rma00934 |
| *dsrN* | Intracellular sulfur oxidation protein DsrN | 156.66 | Vma00806 | Vok00814 | Rma00928 |
| *dsrO* | Intracellular sulfur oxidation protein DsrO | 1224.81 | Vma00808 | Vok00816 | Rma00930 |
| *dsrP* | Intracellular sulfur oxidation protein DsrP | 272.18 | Vma00807 | Vok00815 | Rma00929 |
| *dsrR* | Intracellular sulfur oxidation protein DsrR | 390.80 | Vma00805 | Vok00813 | Rma00354 |
| *Rho3* | Rhodanese-related sulfurtransferase putative | 385.94 | Vma00861 | Vok00872 | Rma01006 |
| *Rho4* | Rhodanese-related sulfurtransferase putative | 826.06 | Vma00928 | Vok00940 | Rma01088 |
| *Rho5* | Rhodanese-related sulfurtransferase putative | 78.84 | Vma00936 | Vok00948 | Rma01095 |
| *sat* | ATP sulfurylase | 3747.82 | Vma00089 | Vok00093 | Rma00099 |
| *soxA* | Sulfur oxidation protein SoxA | 707.70 | Vma00752 | Vok00760 | Rma00864 |
| *soxB* | Sulfur oxidation protein SoxB | 2520.60 | Vma00162 | Vok00167 | Rma00184 |
| *soxX* | Sulfur oxidation protein SoxX | 2006.29 | Vma00755 | Vok00763 | Rma00867 |
| *soxY* | Sulfur oxidation protein SoxY | 1066.81 | Vma00754 | Vok00762 | Rma00866 |
| *soxZ* | Sulfur oxidation protein SoxZ | 793.99 | Vma00753 | Vok00761 | Rma00865 |
| *sqr1* | Sulfide-quinone reductase | 647.84 | Vma00771 | Vok00779 | Rma00887 |
| *sqr2* | Sulfide-quinone reductase | 4903.07 | Vma00977, Vma00978 | Vok00989 | Rma01143 |

**Table S13.** A list of the Calvin cycle-related genes found in three vesicomyid symbiont genomes, with gene expression levels shown for *Ca.* V. marissinica.

| **Gene ID** | **Gene function** | **Expression in *Ca*. V. marissinica (TPM)** | ***Ca*. V. marissinica** | ***Ca*. V. okutanii** | ***Ca.* R. magnifica** |
| --- | --- | --- | --- | --- | --- |
| *cbbM* | ribulose-bisphosphate carboxylase | 8044.49 | Vma00667 | Vok00678 | Rma00762 |
| *pgk* | phosphoglycerate kinase | 3352.107 | Vma00072 | Vok00076 | Rma00078 |
| *gapA* | glyceraldehyde 3-phosphate dehydrogenase | 7902.632 | Vma00071 | Vok00075 | Rma00076 |
| *fba* | fructose-bisphosphate aldolase | 12536.14 | Vma00074 | Vok00078 | Rma00080 |
| *tkt* | Transketolase | 2905.372 | Vma00070 | Vok00074 | Rma00075 |
| *rpiA* | ribose 5-phosphate isomerase A | 449.2981 | Vma00221 | Vok00226 | Rma00254 |
| *prk* | Phosphoribulokinase | 2338.274 | Vma00004 | Vok00004 | Rma00004 |
| *tpi* | triosephosphate isomerase | 2306.762 | Vma01002 | Vok01013 | Rma01170 |
| *rpe* | ribulose-phosphate 3-epimerase | 1304.942 | Vma00995 | Vok01006 | Rma01163 |

**Table S15**. Presence/absence of the orthologues of 16 regulatory genes for essential amino acid biosynthetic pathways in *Escherichia coli* (based on Moran et al. 2005) in three vesicomyid symbiont genomes and their free living relative *Thiomicrospira crunogena*. Candidate genes were identified by BLASTp search against the *E. coli* genome followed by manual correction.

| **Amino acid** | **Regulatory gene** | **Type of regulation** | ***Ca.* V. marissinica** | ***Ca.* V. okutanii** | ***Ca.* R. magnifica** | ***Thiomicrospira crunogena*** |
| --- | --- | --- | --- | --- | --- | --- |
| Arg | *argR* | Repressor | − | − | − | − |
|  | *himA* | Activator/repressor | Vma00598 | Vok00610 | Rma00699 | + |
| Lys | *lysR* | Activator | pseudogene | pseudogene | pseudogene | + |
| Thr | *thrL* | Attenuation | − | − | − | − |
| Val/Ile/Leu | *ilvY* | Activator | − | − | − | + |
|  | *lrp* | Activator | − | − | − | − |
|  | *himA* | Activator/repressor | − | − | − | − |
|  | *ilvL* | Attenuation | − | − | − | − |
| Leu | *leuO* | Activator | − | − | − | + |
|  | *lrp* | Activator | − | − | − | + |
|  | *leuL* | Attenuation | − | − | − | − |
| Trp | *trpR* | Repressor | − | − | − | − |
|  | *trpL* | Attenuation | − | − | − | − |
|  | *lrp* | Activator | − | − | − | − |
| Phe | *pheL* | Attenuation | − | − | − | − |
| His | *hisL* | Attenuation | − | − | − | − |
| Met | *metJ* | Repressor | − | − | − | − |
|  | *metR* | Activator | − | − | Rma01061 | + |
| Gly | *metR* | Activator | − | − | Rma01061 | + |
| Cys | *cysB* | Activator | − | − | − | + |

**Table S25.** Expression level of differentially expressed miRNAs between the gill and foot tissue of *Archivesica marissinica*. The eight miRNAs with higher expression in the gill than in the foot were highlighted in bold. They were predicted to interact with 41 mRNAs that were differentially expressed between the gill and the foot tissues.

| **MiRNA** | **Expression level** | | **log_2_(fold change)** | **Adjusted *p*-value** |
| --- | --- | --- | --- | --- |
|  | **Gill** | **Foot** |  |  |
| chr16_12431 | 15.9 | 0.0 | 5.5 | 3.4E-03 |
| **chr12_7607** | 15.1 | 1.4 | 4.1 | 2.5E-02 |
| **chr13_9052** | 46.7 | 1.7 | 4.0 | 1.5E-03 |
| **chr1_2131** | 1945.4 | 408.4 | 3.8 | 1.5E-03 |
| **chr6_28131** | 190.9 | 21.9 | 3.1 | 6.1E-07 |
| **chr5_26748** | 1682.9 | 361.2 | 2.0 | 4.6E-03 |
| **chr2_20922** | 26.1 | 6.9 | 1.9 | 1.7E-02 |
| **chr13_9054** | 55.9 | 21.7 | 1.3 | 3.7E-02 |
| **chr5_26300** | 93.4 | 37.8 | 1.3 | 3.5E-02 |
| chr17_15033 | 462.9 | 108827.2 | -7.9 | 1.3E-11 |
| chr17_13963 | 0.0 | 71.3 | -5.7 | 8.7E-05 |
| chr9_32836 | 0.0 | 19.7 | -5.0 | 4.6E-03 |
| chr16_12278 | 0.9 | 208.0 | -4.7 | 3.5E-03 |
| chr1_1236 | 23.1 | 258.9 | -4.3 | 1.5E-03 |
| chr14_10577 | 21.9 | 505.0 | -3.8 | 6.3E-06 |
| chr1_1171 | 433.0 | 1563.0 | -3.5 | 2.0E-03 |
| chr18_16482 | 5.8 | 34.0 | -2.9 | 4.6E-03 |
| chr3_22782 | 5.8 | 34.0 | -2.9 | 4.6E-03 |
| chr12_5967 | 30.9 | 485.6 | -2.7 | 3.4E-03 |
| chr18_17662 | 337.4 | 2692.2 | -2.7 | 1.5E-03 |
| chr13_7910 | 6.7 | 41.2 | -2.7 | 3.5E-03 |
| chr10_2816 | 154.1 | 412.8 | -1.3 | 3.7E-02 |

**Table S26.** MiRNA:mRNA interaction sites of the eight miRNAs that were highly expressed in the gill of *Archivesica marissinica*, which included 41 differentially expressed mRNAs between the gill and the foot (The mRNA expression level is shown in Table S27 in a separate Excel file).

| **No.** | **miRNA:mRNA interaction site** |
| --- | --- |
| 1 | chr1_2131:Ama03332 |
| 2 | chr1_2131:Ama04421 |
| 3 | chr12_7607:Ama29959 |
| 4 | chr13_9052:Ama16989 |
| 5 | chr13_9054:Ama16989 |
| 6 | chr2_20922:Ama34369 |
| 7 | chr5_26300:Ama00985 |
| 8 | chr5_26300:Ama01076 |
| 9 | chr5_26300:Ama03138 |
| 10 | chr5_26300:Ama08786 |
| 11 | chr5_26300:Ama09352 |
| 12 | chr5_26300:Ama22675 |
| 13 | chr5_26300:Ama34068 |
| 14 | chr5_26748:Ama07912 |
| 15 | chr5_26748:Ama16989 |
| 16 | chr6_28131:Ama09352 |
| 17 | chr6_28131:Ama19968 |
| 18 | chr6_28131:Ama21026 |
| 19 | chr6_28131:Ama242_5db7b01a |
| 20 | chr12_7607:Ama05962 |
| 21 | chr12_7607:Ama10699 |
| 22 | chr12_7607:Ama18818 |
| 23 | chr5_26300:Ama08512 |
| 24 | chr5_26300:Ama11593 |
| 25 | chr5_26300:Ama15492 |
| 26 | chr5_26300:Ama19220 |
| 27 | chr5_26300:Ama20434 |
| 28 | chr5_26300:Ama31560 |
| 29 | chr5_26300:Ama31690 |
| 30 | chr5_26748:Ama03179 |
| 31 | chr5_26748:Ama18818 |
| 32 | chr5_26748:Ama26962 |
| 33 | chr5_26748:Ama31599 |
| 34 | chr6_28131:Ama11896 |
| 35 | chr6_28131:Ama12800 |
| 36 | chr6_28131:Ama16718 |
| 37 | chr6_28131:Ama21930 |
| 38 | chr6_28131:Ama21932 |
| 39 | chr6_28131:Ama24189 |
| 40 | chr6_28131:Ama24745 |
| 41 | chr6_28131:Ama27966 |
| 42 | chr6_28131:Ama27968 |
| 43 | chr6_28131:Ama29332 |
| 44 | chr6_28131:Ama32985 |
| 45 | chr6_28131:Ama33181 |

**4. References**

Albertin CB, Simakov O, Mitros T, Wang ZY, Pungor JR, Edsinger-Gonzales E, Brenner S, Ragsdale CW, Rokhsar DS, et al. 2015. The octopus genome and the evolution of cephalopod neural and morphological novelties. *Nature*. 524:220-224.

Amano K, Jenkins RG, Ohara M, Kiel S. 2014. Miocene vesicomyid species (Bivalvia) from Wakayama in southern Honshu, Japan. *Nautilus.* 128:9-17.

Bankevich A, Nurk S, Antipov D, Gurevich AA, Dvorkin M, Kulikov AS, Lesin VM, Nikolenko SI, Pham S, Prjibelski AD. 2012. SPAdes: a new genome assembly algorithm and its applications to single-cell sequencing. *J Comp Biol*. 19:455-477.

Bao W, Kojima KK, Kohany O. 2015. Repbase Update, a database of repetitive elements in eukaryotic genomes. *Mobile DNA.* 6:11.

Belcaid M, Casaburi G, McAnulty SJ, Schmidbaur H, Suria AM, Moriano-Gutierrez S, Pankey MS, Oakley TH, Kremer N, Koch EJ, et al. 2019. Symbiotic organs shaped by distinct modes of genome evolution in cephalopods. *Proc Natl Acad Sci USA*. 116:3030-3035.

Benjamini Y, Hochberg Y. 1995. Controlling the false discovery rate: a practical and powerful approach to multiple testing. *J R Stat Soc B*. 289-300.

Benton MJ, Donoghue PCJ, Asher RJ. 2009. Calibrating and constraining molecular clocks. In: Hedges SB, Kumar S, editors. The Timetree of Life. England (Oxford): Oxford University Press. p.35-86.

Benton MJ, Donoghue PC, Asher RJ, Friedman M, Near TJ, Vinther J. 2015. Constraints on the timescale of animal evolutionary history. *Palaeontol Electron.* 18:1-106.

Bolger AM, Lohse M, Usadel B. 2014. Trimmomatic: a flexible trimmer for Illumina sequence data. *Bioinformatics*. 170.

Brudno M, Do CB, Cooper GM, Kim MF, Davydov E, Green ED, Sidow A, Batzoglou S, Program NCS. 2003. LAGAN and Multi-LAGAN: efficient tools for large-scale multiple alignment of genomic DNA. *Genome Res*.13:721-731.

Buchfink B, Xie C, Huson DH. 2015. Fast and sensitive protein alignment using DIAMOND. *Nat Methods.* 12:59-60.

Bushnell B. 2014. BBMap: a fast, accurate, splice-aware aligner. In: Lawrence Berkeley National Lab.(LBNL), Berkeley, CA (United States).

Cantarel BL, Korf I, Robb SM, Parra G, Ross E, Moore B, Holt C, Alvarado AS, Yandell M. 2008. MAKER: an easy-to-use annotation pipeline designed for emerging model organism genomes. *Genome Res.* 18:188-196.

Capella-Gutiérrez S, Silla-Martínez JM, Gabaldón T. 2009. trimAl: a tool for automated alignment trimming in large-scale phylogenetic analyses. *Bioinformatics*. 25:1972-1973.

Chen W, Hasegawa DK, Kaur N, Kliot A, Pinheiro PV, Luan J, Stensmyr MC, Zheng Y, Liu W, Sun H. 2016. The draft genome of whitefly *Bemisia tabaci* MEAM1, a global crop pest, provides novel insights into virus transmission, host adaptation, and insecticide resistance. *BMC Biol*. 14:110.

Chen S, Zhou Y, Chen Y, Gu J. 2018. fastp: an ultra-fast all-in-one FASTQ preprocessor. *Bioinformatics.* 34:i884-i890.

Da Lage J-L, Binder M, Hua-Van A, Janeček Š, Casane D. 2013. Gene make-up: rapid and massive intron gains after horizontal transfer of a bacterial α-amylase gene to Basidiomycetes. *BMC Evol Biol.* 13:40.

Darling AC, Mau B, Blattner FR, Perna NT. 2004. Mauve: multiple alignment of conserved genomic sequence with rearrangements. *Genome Res.* 14:1394-1403.

Dong Y, Zeng Q, Ren J, Yao H, Ruan W, Lv L, He L, Xue Q, Bao Z, Wang S. 2019. The chromosomal-level genome assembly and comprehensive transcriptomes of Chinese razor clam (*Sinonovacula constricta*) with deep-burrowing life style and broad-range salinity adaptation. *bioRxiv*. 735142.

Dudchenko O, Batra SS, Omer AD, Nyquist SK, Hoeger M, Durand NC, Shamim MS, Machol I, Lander ES, Aiden AP. 2017. *De novo* assembly of the Aedes aegypti genome using Hi-C yields chromosome-length scaffolds. *Science.* 356:92-95.

Durand NC, Shamim MS, Machol I, Rao SS, Huntley MH, Lander ES, Aiden EL. 2016. Juicer provides a one-click system for analyzing loop-resolution Hi-C experiments. *Cell Syst.* 3:95-98.

Edgar RC. 2004. MUSCLE: multiple sequence alignment with high accuracy and high throughput. *Nucleic Acids Res.* 32:1792-1797.

Edgar RC. 2010. Search and clustering orders of magnitude faster than BLAST. *Bioinformatics.* 26:2460-2461.

Elbourne LD, Tetu SG, Hassan KA, Paulsen IT. 2017. TransportDB 2.0: a database for exploring membrane transporters in sequenced genomes from all domains of life. *Nucleic Acids Res*. 45:D320-D324.

Emms DM, Kelly S. 2015. OrthoFinder: solving fundamental biases in whole genome comparisons dramatically improves orthogroup inference accuracy. *Genome Biol*. 16:157.

Enright AJ, John B, Gaul U, Tuschl T, Sander C, Marks DS. 2003. MicroRNA targets in Drosophila. *Genome Biol*. 5:R1.

Fichant G, Basse M-J, Quentin Y. 2006. ABCdb: an online resource for ABC transporter repertories from sequenced archaeal and bacterial genomes. *FEMS Microbiol Lett.* 256:333-339.

Finn RD, Coggill P, Eberhardt RY, Eddy SR, Mistry J, Mitchell AL, Potter SC, Punta M, Qureshi M, Sangrador-Vegas A. 2016. The Pfam protein families database: towards a more sustainable future. *Nucleic Acids Res*. 44:D279-D285.

Friedländer MR, Mackowiak SD, Li N, Chen W, Rajewsky N. 2011. miRDeep2 accurately identifies known and hundreds of novel microRNA genes in seven animal clades. *Nucleic Acids Res.* 40:37-52.

Gómez-Chiarri M, Warren WC, Guo X, Proestou D. 2015. Developing tools for the study of molluscan immunity: the sequencing of the genome of the eastern oyster, Crassostrea virginica. *Fish Shellfish Immun.* 46:2-4.

Grant JR, Stothard P. 2008. The CGView Server: a comparative genomics tool for circular genomes. *Nucleic Acids Res.* 36:W181-W184.

Haas BJ, Delcher AL, Mount SM, Wortman JR, Smith Jr RK, Hannick LI, Maiti R, Ronning CM, Rusch DB, Town CD, et al. 2003. Improving the *Arabidopsis* genome annotation using maximal transcript alignment assemblies. *Nucleic Acids Res.* 31:5654-5666.

Haas BJ, Salzberg SL, Zhu W, Pertea M, Allen JE, Orvis J, White O, Buell CR, Wortman JR. 2008. Automated eukaryotic gene structure annotation using EVidenceModeler and the Program to Assemble Spliced Alignments. *Genome Biol*. 9:R7.

Haas BJ, Papanicolaou A, Yassour M, Grabherr M, Blood PD, Bowden J, Couger MB, Eccles D, Li B, Lieber M, et al. 2013. *De novo* transcript sequence reconstruction from RNA-seq using the Trinity platform for reference generation and analysis. *Nat Protoc.* 8:1494-1512.

Harada M, Yoshida T, Kuwahara H, Shimamura S, Takaki Y, Kato C, Miwa T, Miyake H, Maruyama T. 2009. Expression of genes for sulfur oxidation in the intracellular chemoautotrophic symbiont of the deep-sea bivalve *Calyptogena okutanii*. *Extremophiles*. 13:895.

Hayes KA, Cowie RH, Jørgensen A, Schultheiß R, Albrecht C, Thiengo SC. 2009. Molluscan models in evolutionary biology: apple snails (Gastropoda: Ampullariidae) as a system for addressing fundamental questions. *Am Malacol Bull*. 27:47-58.

Huerta-Cepas J, Forslund K, Coelho LP, Szklarczyk D, Jensen LJ, von Mering C, Bork P. 2017. Fast genome-wide functional annotation through orthology assignment by eggNOG-mapper. *Mol Biol Evol*. 34:2115-2122.

Husnik F, McCutcheon JP. 2017. Functional horizontal gene transfer from bacteria to eukaryotes. Nat Rev Microbiol. 16(2):67–79.

Jörger KM, Stöger I, Kano Y, Fukuda H, Knebelsberger T, Schrödl M. 2010. On the origin of Acochlidia and other enigmatic euthyneuran gastropods, with implications for the systematics of Heterobranchia. *BMC Evol Biol*. 10: 323.

Kajitani R, Yoshimura D, Okuno M, Minakuchi Y, Kagoshima H, Fujiyama A, Kubokawa K, Kohara Y, Toyoda A, Itoh T. 2019. Platanus-allee is a *de novo* haplotype assembler enabling a comprehensive access to divergent heterozygous regions. *Nat Commun.* 10:1-15.

Kalyaanamoorthy S, Minh BQ, Wong TK, von Haeseler A, Jermiin LS. 2017. ModelFinder: fast model selection for accurate phylogenetic estimates. *Nat Methods.* 14:587.

Katoh K, Standley DM. 2013. MAFFT multiple sequence alignment software version 7: improvements in performance and usability. *Mol Biol Evol.* 30:772-780.

Kertesz M, Iovino N, Unnerstall U, Gaul U, Segal E. 2007. The role of site accessibility in microRNA target recognition. *Nat Genet*. 39:1278-1284.

Kim D, Langmead B, Salzberg SL. 2015. HISAT: a fast spliced aligner with low memory requirements. *Nat Methods*. 12:357.

Kimura M. 1980. A simple method for estimating evolutionary rates of base substitutions through comparative studies of nucleotide sequences. *J Mol Evol*. 16:111-120.

Koren S, Walenz BP, Berlin K, Miller JR, Bergman NH, Phillippy AM. 2017. Canu: scalable and accurate long-read assembly via adaptive k-mer weighting and repeat separation. *Genome Res* 27:722-736.

Koutsovoulos G, Kumar S, Laetsch DR, Stevens L, Daub J, Conlon C, Maroon H, Thomas F, Aboobaker AA, Blaxter M. 2016. No evidence for extensive horizontal gene transfer in the genome of the tardigrade *Hypsibius dujardini*. *Proc Natl Acad Sci USA*. 113:5053-5058.

Kozomara A, Birgaoanu M, Griffiths-Jones S. 2018. miRBase: from microRNA sequences to function. *Nucleic Acids Res*. 47:D155-D162.

Lan Y, Sun J, Zhang W, Xu T, Zhang Y, Chen C, Feng D, Wang H, Tao J, Qiu JW, et al. 2019. Host-symbiont interactions in deep-sea chemosymbiotic vesicomyid clams: insights from transcriptome sequencing. *Front Mar Sci*. 6:680.

Langmead B, Trapnell C, Pop M, Salzberg SL. 2009. Ultrafast and memory-efficient alignment of short DNA sequences to the human genome. *Genome Biol*. 10:R25.

Levin HL, Moran JV. 2011. Dynamic interactions between transposable elements and their hosts. *Nat Rev Genet*. 12:615–627.

Li H, Handsaker B, Wysoker A, Fennell T, Ruan J, Homer N, Marth G, Abecasis G, Durbin R, Genome Project Data Processing S. 2009. The Sequence Alignment/Map format and SAMtools. *Bioinformatics*. 25:2078-2079.

Li C, Liu X, Liu B, Ma B, Liu F, Liu G, Shi Q, Wang C. 2018. Draft genome of the Peruvian scallop *Argopecten purpuratus*. *GigaScience* 7:giy031.

Li Y, Sun X, Hu X, Xun X, Zhang J, Guo X, Jiao W, Zhang L, Liu W, Wang J. 2017. Scallop genome reveals molecular adaptations to semi-sessile life and neurotoxins. *Nat Commun*. 8:1721.

Lieberman-Aiden E, Van Berkum NL, Williams L, Imakaev M, Ragoczy T, Telling A, Amit I, Lajoie BR, Sabo PJ, Dorschner MO, et al. 2009. Comprehensive mapping of long-range interactions reveals folding principles of the human genome. *Science*. 326:289-293.

Luo Y-J, Takeuchi T, Koyanagi R, Yamada L, Kanda M, Khalturina M, Fujie M, Yamasaki S-i, Endo K, Satoh N. 2015. The Lingula genome provides insights into brachiopod evolution and the origin of phosphate biomineralization. *Nat Commun*. 6:8301.

Love MI, Huber W, Anders S. 2014. Moderated estimation of fold change and dispersion for RNA-seq data with DESeq2. *Genome Biol*. 15:550.

Masonbrink RE, Purcell CM, Boles SE, Whitehead A, Hyde JR, Seetharam AS, Severin AJ. 2019. An annotated genome for Haliotis rufescens (red abalone) and resequenced green, pink, pinto, black, and white abalone species. *Genome Biol Evol*. 11:431-438

Mun S, Kim Y-J, Markkandan K, Shin W, Oh S, Woo J, Yoo J, An H, Han K. 2017. The whole-genome and transcriptome of the Manila clam (*Ruditapes philippinarum*). *Genome Biol Evol*. 9:1487-1498.

Nguyen L-T, Schmidt HA, von Haeseler A, Minh BQ. 2014. IQ-TREE: a fast and effective stochastic algorithm for estimating maximum-likelihood phylogenies. *Mol Biol Evol.* 32:268-274.

Parks DH, Imelfort M, Skennerton CT, Hugenholtz P, Tyson GW. 2015. CheckM: assessing the quality of microbial genomes recovered from isolates, single cells, and metagenomes. *Genome Res*. 25:1043-1055.

Patro R, Duggal G, Love MI, Irizarry RA, Kingsford C. 2017. Salmon provides fast and bias-aware quantification of transcript expression. *Nat Methods*. 14:417.

Petersen TN, Brunak S, von Heijne G, Nielsen H. 2011. SignalP 4.0: discriminating signal peptides from transmembrane regions. *Nat Methods.* 8:785-786.

Porebski S, Bailey LG, Baum BR. 1997. Modification of a CTAB DNA extraction protocol for plants containing high polysaccharide and polyphenol components. *Plant Mol Biol Rep*. 15:8-15.

Quinlan AR, Hall IM. 2010. BEDTools: a flexible suite of utilities for comparing genomic features. *Bioinformatics.* 26:841-842.

Ran Z, Li Z, Yan X, Liao K, Kong F, Zhang L, Cao J, Zhou C, Zhu P, He S, et al. 2019. Chromosome‐level genome assembly of the razor clam Sinonovacula constricta (Lamarck, 1818). *Mol Ecol Resour.* 19:1647-1658.

Ruan J, Li H. 2020. Fast and accurate long-read assembly with wtdbg2. *Nat Methods.* 17:155-158.

Seemann T. 2014. Prokka: rapid prokaryotic genome annotation. *Bioinformatics*. 30:2068-2069.

Servant N, Varoquaux N, Lajoie BR, Viara E, Chen C-J, Vert J-P, Heard E, Dekker J, Barillot E. 2015. HiC-Pro: an optimized and flexible pipeline for Hi-C data processing. *Genome Biol*. 16:259.

Shannon P, Markiel A, Ozier O, Baliga NS, Wang JT, Ramage D, Amin N, Schwikowski B, Ideker T. 2003. Cytoscape: a software environment for integrated models of biomolecular interaction networks. *Genome Res*. 13:2498-2504.

Simakov O, Marletaz F, Cho S-J, Edsinger-Gonzales E, Havlak P, Hellsten U, Kuo D-H, Larsson T, Lv J, Arendt D, et al. 2013. Insights into bilaterian evolution from three spiralian genomes. *Nature.* 493:526-531.

Smit AF, Hubley R. 2008-2015. RepeatModeler Open-1.0. Available fom <http://www>. repeatmasker. org.

Sonnhammer EL, Eddy SR, Durbin R. 1997. Pfam: a comprehensive database of protein domain families based on seed alignments. *Proteins* 28:405-420.

Stanke M, Morgenstern B. 2005. AUGUSTUS: a web server for gene prediction in eukaryotes that allows user-defined constraints. *Nucleic Acids Res*. 33:W465-W467.

Stewart FJ, Cavanaugh CM. 2009. Pyrosequencing analysis of endosymbiont population structure: co‐occurrence of divergent symbiont lineages in a single vesicomyid host clam. *Environ Microbiol*. 11:2136-2147.

Stöger I, Sigwart J, Kano Y, Knebelsberger T, Marshall B, Schwabe E, Schrödl M. 2013. The Continuing debate on deep molluscan phylogeny: evidence for Serialia (Mollusca, Monoplacophora + Polyplacophora). *BioMed Res Intl*. 2013:407072.

Sun J, Mu H, Ip JC, Li R, Xu T, Accorsi A, Sánchez Alvarado A, Ross E, Lan Y, Sun Y, et al. 2019. Signatures of Divergence, Invasiveness, and Terrestrialization Revealed by Four Apple Snail Genomes. *Mol Biol Evol*. 36:1507-1520.

Sun J, Chen C, Miyamoto N, Li R, Sigwart JD, Xu T, Sun Y, Wong WC, Ip JC, Zhang W, et al. 2020. The Scaly-foot Snail genome and implications for the origins of biomineralised armour. *Nat Commun.* 11:1-12.

Syberg-Olsen M, F. Husnik F. 2018. Pseudofinder. GitHub repository: https://github.com/filip-husnik/pseudo-finder/.

Takeuchi T, Kawashima T, Koyanagi R, Gyoja F, Tanaka M, Ikuta T, Shoguchi E, Fujiwara M, Shinzato C, Hisata K. 2012, et al. Draft genome of the pearl oyster *Pinctada fucata*: a platform for understanding bivalve biology. *DNA Res*. 19:117-130.

Xu T, Feng D, Tao J, Qiu JW. 2019. A new species of deep-sea mussel (Bivalvia: Mytilidae: Gigantidas) from the South China Sea: Morphology, phylogenetic position, and gill-associated microbes. *Deep Sea Res Pt I*.146:79-90.

Wang S, Zhang J, Jiao W, Li J, Xun X, Sun Y, Guo X, Huan P, Dong B, Zhang L, et al. 2017. Scallop genome provides insights into evolution of bilaterian karyotype and development. *Nat Ecol Evol*. 1(5):120.

Yan X, Nie H, Huo Z, Ding J, Li Z, Yan L, Jiang L, Mu Z, Wang H, Meng X, et al. 2019. Clam genome sequence clarifies the molecular basis of its benthic adaptation and extraordinary shell color diversity. *iScience*. 19:1225-1237.

Yang Z. 2007. PAML 4: phylogenetic analysis by maximum likelihood. *Mol Biol Evol*. 24:1586-1591.

Yang M, Gong L, Sui J, Li X. 2019. The complete mitochondrial genome of *Calyptogena marissinica* (Heterodonta: Veneroida: Vesicomyidae): Insight into the deep-sea adaptive evolution of vesicomyids. PloS ONE. 14(9), e0217952.

Young MD, Wakefield MJ, Smyth GK, Oshlack A. 2010. Gene ontology analysis for RNA-seq: accounting for selection bias. *Genome Biol*. 11:R14.

Zhang G, Fang X, Guo X, Li L, Luo R, Xu F, Yang P, Zhang L, Wang X, Qi H, et al. 2012. The oyster genome reveals stress adaptation and complexity of shell formation. *Nature*. 490:49-54.

Zhang SV, Zhuo L, Hahn MW. 2016. AGOUTI: improving genome assembly and annotation using transcriptome data. *GigaScience*. 5:s13742-13016-10136-13743.

Zhu B-H, Xiao J, Xue W, Xu G-C, Sun M-Y, Li J-T. 2018. P_RNA_scaffolder: a fast and accurate genome scaffolder using paired-end RNA-sequencing reads. *BMC Genomics.* 19:175.

Zimin AV, Marçais G, Puiu D, Roberts M, Salzberg SL, Yorke JA. 2013. The MaSuRCA genome assembler. *Bioinformatics*. 29:2669-2677.

Zou C, Lehti-Shiu MD, Thibaud-Nissen F, Prakash T, Buell CR, Shiu S-H. 2009. Evolutionary and expression signatures of pseudogenes in *Arabidopsis* and rice. *Plant Physiol*. 151:3-15.
